# Phosphinotripeptidic Inhibitors of Leucylaminopeptidases

**DOI:** 10.3390/ijms22105090

**Published:** 2021-05-11

**Authors:** Michał Jewgiński, Kinga Haremza, Jesús M. de los Santos, Zouhair Es Sbai, Bartosz Oszywa, Małgorzata Pawełczak, Francisco Palacios, Rafał Latajka

**Affiliations:** 1 Department of Bioorganic Chemistry, Wrocław University of Science and Technology, Wybrzeże Wyspiańskiego 27, 50-370 Wrocław, Poland; kinga.haremza@gmail.com (K.H.); rafal.latajka@pwr.edu.pl (R.L.); 2 Department of Organic Chemistry I, Faculty of Pharmacy, Lascaray Research Center, University of the Basque Country (UPV/EHU), Paseo de la Universidad, 7, 01006 Vitoria, Spain; jesus.delossantos@ehu.es (J.M.d.l.S.); zouhair.essbai@ehu.eus (Z.E.S.); francisco.palacios@ehu.es (F.P.); 3 Institute of Chemistry, University of Opole, Oleska 48, 45-052 Opole, Poland; bartoszoszywa@interia.pl (B.O.); pamal@uni.opole.pl (M.P.)

**Keywords:** molecular modeling, LAP inhibitors, barley aminopeptidase inhibitor, phosphinate pseudopeptide, ligand-enzyme interaction, organophosphorus compound

## Abstract

Phosphinate pseudopeptide are analogs of peptides containing phosphinate moiety in a place of the amide bond. Due to this, the organophosphorus fragment resembles the tetrahedral transition state of the amide bond hydrolysis. Additionally, it is also capable of coordinating metal ions, for example, zinc or magnesium ions. These two properties of phosphinate pseudopeptides make them an ideal candidate for metal-related protease inhibitors. This research investigates the influence of additional residue in the P2 position on the inhibitory properties of phosphinopeptides. The synthetic strategy is proposed, based on retrosynthetic analysis. The N-C-P bond formation in the desired compounds is conveniently available from the three-component condensation of appropriate amino components, aldehydes, and hypophosphorous acid. One of the crucial synthetic steps is the careful selection of the protecting groups for all the functionals. Determination of the inhibitor activity of the obtained compounds has been done using UV-Vis spectroscopy and standard substrate _L_-Leu-*p*-nitroanilide toward the enzymes isolated from the porcine kidney (SsLAP, *Sus scrofa* Leucine aminopeptidase) and barley seeds (HvLAP, *Hordeum vulgare* Leucine aminopeptidase). An efficient procedure for the preparation of phosphinotripeptides has been performed. Activity test shown that introduction of additional residue into P2 position obtains the micromolar range inhibitors of SsLAP and HvLAP. Moreover, careful selection of the residue in the P2 position should improve its selectivity toward mammalian and plant leucyl aminopeptidases.

## 1. Introduction

The tetrahedral geometry of the phosphinate group introduced into the peptide chain makes these analogs one of the most potent, reversible, and competitive inhibitors of metalloproteases [1,2,3]. This phosphinate moiety mimics the structural and electronic features of the tetrahedral transition state intermediate occurring during the enzymatic hydrolysis of the amide bond. Additionally, this group is also capable of coordinating the metal ion in the active site, which could also block the catalytic activity.

Leucyl aminopeptidases (E.C. 3.4.11.1) are the enzymes from the metallo-dependent hydrolase family, responsible for the cleavage of N-terminal amino acids from the polypeptide substrates—due to this, they play an important role in the physiological processing and degradation of protein and peptide. In mammals, the activity of LAP is involved in the physiological metabolism of regulatory and bioactive peptides, as well as in antigen presentation [4,5]. The disturbed activity of LAP has been connected with various pathological disorders. The therapeutic application of the enzyme is connected with its role during metastasis and tumorigenesis [4]. It has also been shown the LAP is a key enzyme in the process of glutathione turnover in the liver [6]. This enzyme is found in human pathogens, e.g., *Staphylococcus aureus* or *Plasmodium falciparum*, and is required for virulence or digestive proteolysis and nutrition delivery [7]. The elevated level of LAP has also been connected with certain pathological stress states, including inflammation, cataracts, cancer, or HIV infection [8,9,10,11,12]. Potential therapeutic significance, resulting from the biological role of LAP, constantly stimulates research in the area of the methods of regulating its activity.

Although bacterial, parasitic, and animal aminopeptidase are well investigated, their plant counterparts still required further research. The determination of their substrate specificity, as well as the design of selective inhibitors, requires continuous studies. The presence of aminopeptidases has been observed in plant parts characterized by intensive processes of synthesis, conversion, or degradation of the protein [5,13,14,15,16,17].

**Figure 1:**
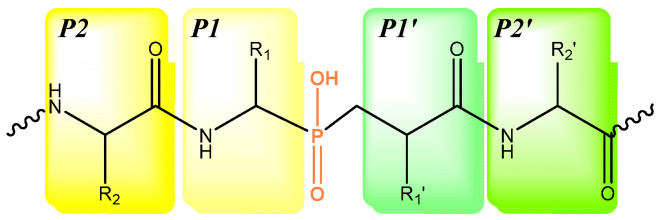
Schematic structure of phosphinic peptide with marked position P2-P2’.

Within this publication, the application of the phosphinic analogs of tripeptide (see Figure 1) as inhibitors of leucine aminopeptidase and plant aminopeptidase will be discussed. It is known that the selectivity of such kinds of molecules is primarily dependent on the structural feature of residues in positions Pn and Pn’ (see Figure 1). The structure and character of these residues are crucial for the matching process of the inhibitor to the enzyme binding pockets. The optimization of the character of residues in positions P1 and P1’ in phosphinic dipeptides obtain the first most potent unnatural inhibitors of LAP [18]. The further investigation, focused on modification of side chains in the positions P1 and P1′, lead to numerous phosphinate and phosphinic dipeptidic inhibitors of LAP [19,20,21,22,23,24].

Because both porcine LAP and barley aminopeptidase belong to exopeptidases, which relieve N-terminal residue, structural modification of inhibitors was usually limited to residues in positions P1 and/or P1′. Grembecka et al. showed that the application of the molecular modeling allowed to optimize the character of the residues side chains in positions P1 and P1′ in results giving the most potent inhibitors of LAP: hPheP[CH_2_]Phe and hPheP[CH_2_]Tyr. However, within this study, we have investigated the influence of residues in the P2 on inhibitory activity. It would be worth checking whether the introduction of additional residue in the P2 position keeps at least moderate inhibitor activity and changes the selectivity toward mammalian and plant aminopeptidase.

## 2. Results and Discussions

### 2.1. Chemistry

**Figure 2:**
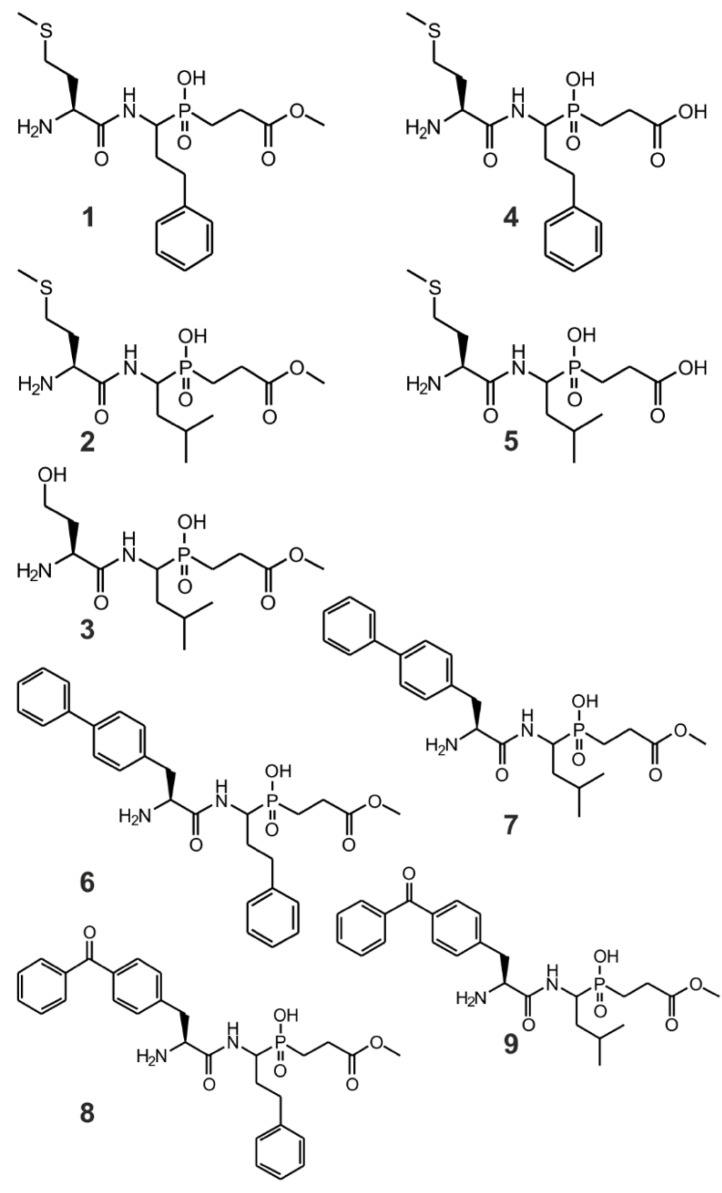
Putative phosphinic pseudotripeptides derived from leucine and homophenylalanine containing modified P2 substituents as inhibitors of leucylaminopeptidase.

**Scheme 1:**
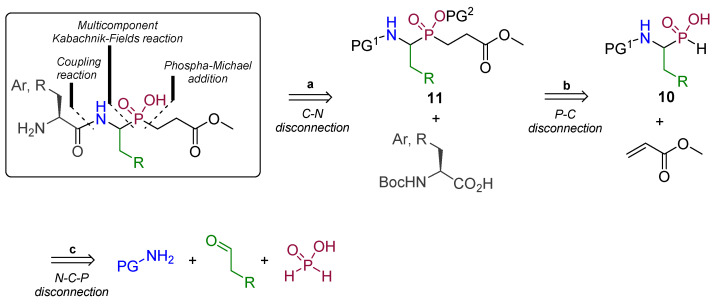
Retrosynthetic analysis of phosphinic pseudotripeptides derived from leucine and homophenylalanine.

The synthetic targets of this work were phosphinic pseudotripeptide analogs **1**–**9** of the structure, shown in Figure 2. The designed compounds possess several functional groups of various characters, and thus, their synthesis is not trivial. To confront this challenge, a retrosynthetic analysis has been performed for these phosphinopeptides inhibitors (Scheme 1). The N-C-P bond formation in α-amino-H-phosphinic substrates **10** is conveniently available from the three-component condensation of appropriate amino components, aldehydes, and hypophosphorous acid (Scheme 1, pathway c). With the suitable protected phosphinic amino acid analogs **10** in hand, the Michael addition of these phosphorus nucleophiles to appropriate carbon electrophiles (typically acrylic esters) is the most commonly applied method for the phosphinate pseudopeptidic bond formation (P-C bond formation) (Scheme 1, pathway b). The availability of the corresponding C-terminal ester **11** would allow multidirectional diversification of the P1 position at the final step of the synthesis through coupling reaction at the N-terminus with different N-protected α-amino acids (C-N bond formation) (pathway a, Scheme 1).

**Scheme 2:**
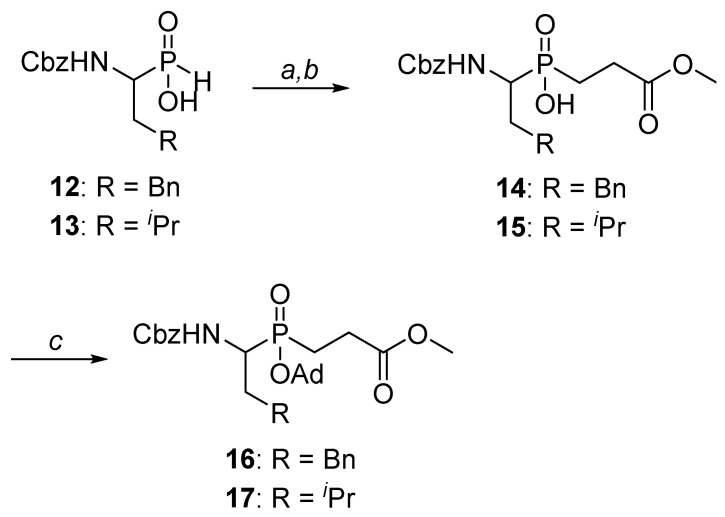
Synthesis of the key building blocks **16** and **17** bearing the side chains of pseudo-HPhe and -Leu, respectively, and required for preparing the target phosphinopeptides **1**–**9**. Reagents and conditions: (a) HMDS, 110 °C; (b) H_2_C=CHCO_2_Me, 90 °C, 66–74%; (c) 1-AdBr, Ag_2_O, CHCl_3_, 65 °C, 87–90%.

Scheme 2 outlined the synthesis of the designed synthons **16** and **17** to prepare the target phosphinic pseudotripeptides **1**–**9**. Careful choice of the protecting groups for three functions present in the molecules of phosphinopeptides precursors is crucial, since their selective removal at the desired synthetic step is an absolute requirement. Different approaches to prepare P-H substrates have been reported in the literature. Early work by Schmidt [25] demonstrated the addition of hypophosphorous acid to imines to give N-substituted α-aminoalkylphosphinic acids. The condensation of an aldehyde, hypophosphorous acid, and an amine as the nitrogen source can also be used to prepare these derivatives [2]. In our case, we began with the use of the three-component reaction. Even though the preparative yields are not high, the main advantages of this approach are low cost and simplicity. Thus, the starting aminophosphinic acid analogs derived from homophenylalanine **12** (R = Bn) and leucine **13** (R = iPr) (Scheme 2), were synthesized in the reaction of an appropriate aldehyde (RCH_2_CHO), diphenylmethylamine hydrochloride (Ph_2_CNH_2_·HCl), and hypophosphorous acid (H_2_P(O)OH), using a variant of the multicomponent Kabachnik-Fields reaction. The diphenylmethyl group can be hydrolyzed from adducts under acidic conditions to obtain free α-aminophosphoric acids. The free amino group in the former α-aminophosphoric acids can subsequently be protected by the benzyloxycarbonyl group (Cbz) in a water solution [26] to give the starting aminophosphinic acid analogs **12** and **13** (Scheme 2). Phosphinic pseudodipeptides **14** and **15**, containing a homophenylalanyl or leucyl aminophosphinic acid, respectively, were synthesized using an approach based on a phospha-Michael addition, which relies on a well-established procedure developed previously [27,28]. Hence, N-benzyloxycarbonylaminophosphinic acids **12** and **13** were activated to the more nucleophilic tervalent ester form by heating with hexamethyldisilazane, followed by adding methyl acrylate as the Michael acceptor. After work-up and purification, the protected pseudopeptides **14** and **15** were obtained in moderate yields (Scheme 2). After a thorough overview of the literature, numerous contradictory reports concerning the protection of the hydroxyphosphinyl moiety have been found. We envisaged that proper protection of the hydroxyphosphinyl moiety is essential for the efficient preparation of phosphinopeptides. Some researchers claim that protection of the hydroxyphosphinyl function is not necessary during peptide coupling reactions, either on the solid-phase [29] or in solution [30,31,32], while others strongly emphasize the serious limitations of coupling methods when phosphinopeptides are not properly protected at the phosphorus center [33,34,35]. Such last evidence is strongly supported by this work, since all attempts to use deprotection products coming from **14** and **15** in the peptide coupling reaction were unsuccessful. This also proves the potential reactivity of the phosphinic acid during the decoupling reaction. The adamantyl group was chosen as a protecting group [1,20,28,36,37], since it can be removed easily under the classical deprotection conditions required for Boc-protecting amino acids coupled in the last step of the synthesis. The introduction of the adamantyl group was easily achieved by refluxing phosphinic acids **14** and **15** with adamantly bromide (1-AdBr) and silver oxide in chloroform. Fully protected pseudodipeptidic blocks derived from homophenylalanine **16** (R = Bn) and leucine **17** (R = iPr) are obtained in excellent yields (Scheme 2) and used as building blocks for incorporation into peptides in solution.

**Scheme 3:**
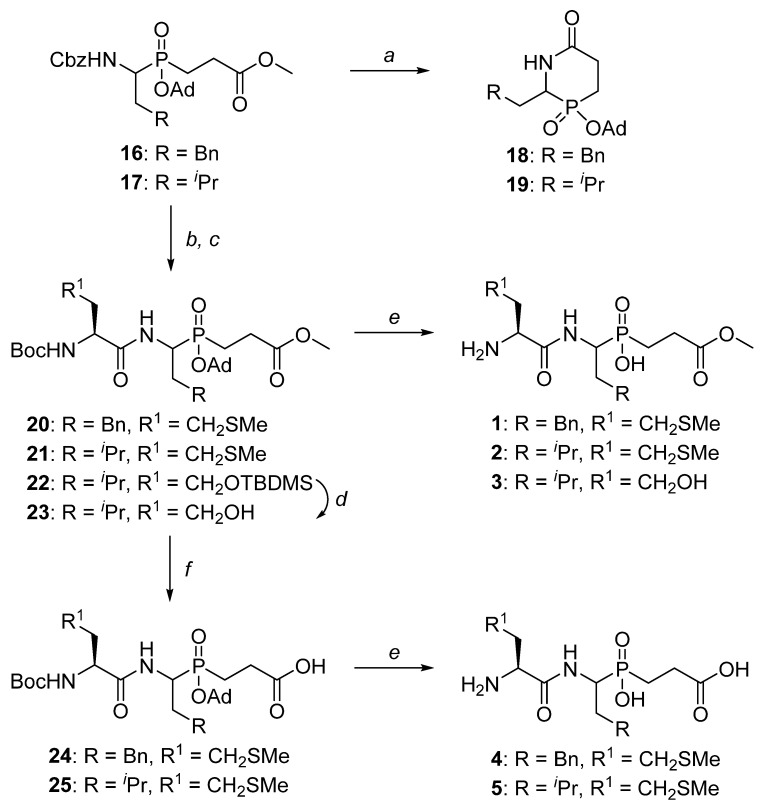
Synthesis of phosphinic pseudotripeptide analogs **1**–**3** and their free acids **4** and **5**. Reagents and conditions: (a) HCO_2_NH_4_/Pd-C, MeOH, reflux or rt; (b) H_2_/Pd-C, MeOH; (c) Boc-_L_-Met-OH (R^1^ = CH_2_SMe) or Boc-_L_-Hse(TBDMS)-OH (R^1^ = CH_2_OTBDMS), HOBt, EDC, TEA, DMF, 51–70%; (d) TBAF, THF, 0 °C to rt, 70%; (e) TFA/CH_2_Cl_2_ (1:1), 85–99%; (f) NaOH/MeOH (0.4 M) them 0.5 M HCl, pH 1, 98–99%.

Phosphinic pseudodipeptides blocks **16** and **17** were subjected to coupling reaction at the N-terminus with different N-protected _L_-amino acids, leading to various **P2** diversified phosphinic tripeptides **1**–**9**. Prior to to the peptide coupling reaction, the benzyloxycarbonyl group might be removed by hydrogenolysis or by the action of HBr in AcOH. When deblocking the Cbz group of **16** and **17** using the method described by Anwer and Spatola [38], which utilizes ammonium formate as a hydrogen donor, in the presence of palladium/carbon catalyst, we found the formation of the pseudodiketopiperazines **18** and **19** as the main product of the reaction (Scheme 3), similarly as described in the literature [28]. Satisfactory results for the decarbobenzyloxylation of compounds **16** and **17** were obtained by means of catalytic hydrogenation using H_2_ in the presence of Pd/C catalyst. The N-terminus elongation approach was adopted to prepare the final phosphinic pseudotripeptides, and in all instances, coupling reactions were achieved with N-Boc-methionine (Boc-_L_-Met-OH) or TBDMS-protected N-Boc-homoserine (Boc-_L_-Hse(TBDMS)-OH—was prepared, starting from _L_-Hse-OH through protection first the hydroxyl group as OTBDMS [39] followed by protection of the N-terminus as N-Boc protecting group [40]) mediated by peptide coupling agents EDC-HOBT in DMF [41,42]. This procedure appeared to be a simple and satisfactory method for the N-terminus elongation of the individual phosphinic dipeptides in solution since, after standard work-up and purification on flash-column chromatography, tripeptide analogs **20**–**22** were obtained in satisfactory yields (51–70%). Finally, deprotection of both N-terminus and hydroxyphosphinyl [43] groups in **20**–**21** under acidic conditions enabled us to obtain the desired fully deprotected phosphinotripeptides **1**–**2**. Formation of compound **3** entails O-TBDMS deprotection of pseudotripeptide **22** by using standard conditions [44], previously to the removal of the N-Boc and P-Ad protecting groups as before (Scheme 3).

Phosphinotripeptides methyl esters **20** and **21** were subjected to hydrolysis by the use of NaOH in MeOH followed by careful adjustment of pH by dilute hydrochloric acid. The saponification of the methyl ester **20** and **21**, in spite of the presence of the adamantyl ester, is completely selective and allows for preparing phosphinotripeptides **24** and **25**, respectively [28]. Consecutive removal of N-Boc and P-adamantyl protecting groups in acidic conditions gave the target compounds as free acids **4** and **5**. Both steps proceeded practically quantitatively (with yields exceeding 98%).

**Scheme 4:**
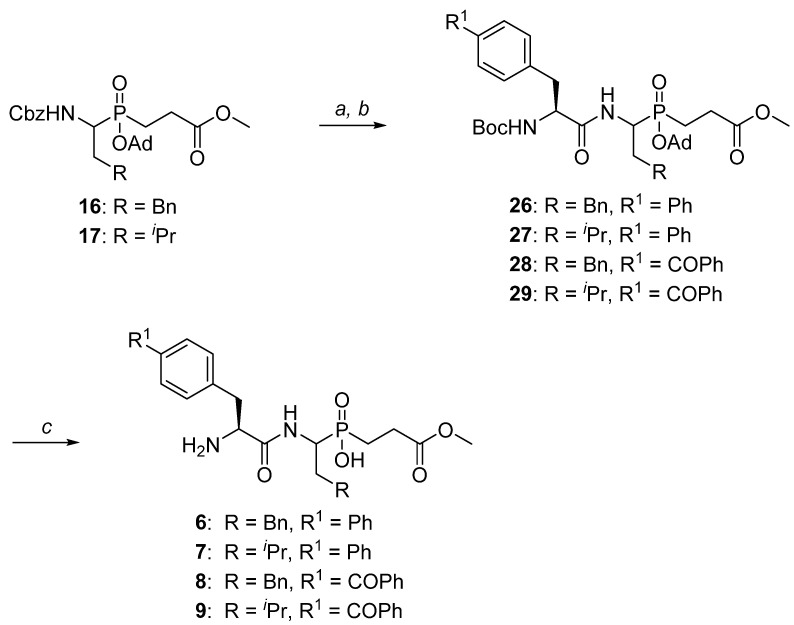
Synthesis of phosphinic pseudotripeptide analogs **6**–**9**. Reagents and conditions: (a) H_2_/Pd-C, MeOH; (b) Boc-_L_-Bip-OH (R^1^ = Ph) or Boc_-L_-Bpa-OH (R^1^ = COPh), HOBt, EDC, TEA, DMF, 50–76%; (c) TFA/CH_2_Cl_2_ (1:1), 78–99%.

When a similar methodology was used, tripeptide analogs **6**–**9** were synthesized (Scheme 4). Debenzylation of the of phosphinic pseudodipeptides blocks **16** and **17** at the N-terminus, followed by the assembly with the suitable N-Boc protected derivative of Bip (N-Boc-_L_-Bip-OH, N-Boc-_L_-4,4′-biphenylalanine) and Bpa (N-Boc-_L_-Bpa-OH, N-Boc-p-benzoyl-_L_-phenylalanine) by using peptide coupling agents EDC-HOBT in DMF [41,42], afforded pseudotripeptide derivatives **26**–**29** in satisfactory yields (50–76%). Removal of the N-Boc and P-adamantyl protecting groups was performed in acidic conditions (TFA/CH_2_Cl_2_, 1:1), providing inhibitors **6**–**9**.

### 2.2. Enzyme Inhibition

The obtained phosphinopeptides were tested as inhibitors of mammalian leucine aminopeptidase and plant aminopeptidase. As the model of mammalian enzyme, the porcine leucine aminopeptidase (SsLAP) has been selected, while as the plant enzyme model, the barley aminopeptidase has been chosen. LAP (leucine aminopeptidase) is the multifunctional broad-band specific zinc-dependent aminopeptidase. The structure, mechanism of the molecular action, as well as therapeutic significance of these enzymes, are well known [5,45]. Barley aminopeptidase HvLAP is an example of plant aminopeptidase, catalyzing the removal of the N-terminal amino acids. As shown in our previous studies, HvLAP preferably removes hydrophobic residue in the P1 position [46]. However, Kawaguchi and Doi showed that HvLAP has also got endopeptidase properties [47]. HvLAP is the metalloprotease which activity is modulated by several metal ions. As shown in our previous studies, HvLAP activity is increased by Ca^2+^, Mg^2+^, and Mn^2+^ ions, and is decreased by Co^2+^, Cd^2+^, Zn^2+^, and Fe^2+^ ions.

**Figure 3:**
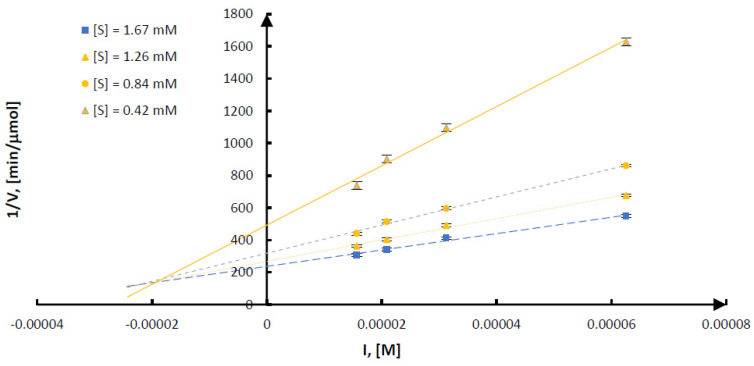
The designation of type inhibition and the inhibition constant K_I_ by Dixon method for compound **3** in the presence of SsLAP.

**Figure 4:**
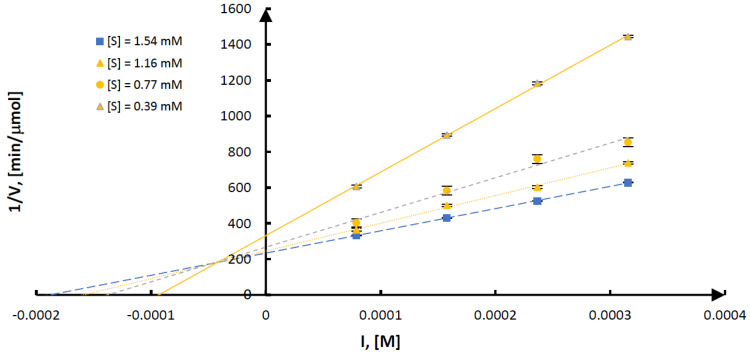
The designation of type inhibition and the inhibition constant K_I_ by Dixon method for compound **1** in the presence of HvLAP.

The inhibition type, mechanism, and inhibition constants (Ki) were determined using graphical methods (see Figure 3 and Figure 4 and Tables S1 and S2). The inhibition type was determined by the Lineweaver-Burk plot based on the results of the inhibitory effect of the compounds on the hydrolytic activity of _L_-Leu-p-nitroanilide. The synthesized molecules, as it is shown in Table 1, exhibited moderate micromolar inhibitory activity toward SsLAP and HvLAP and appeared to be competitive inhibitors. It should be remembered that inhibitor activity was appointed for the racemic mixtures. All investigated compound **1**–**9** contains only *S* isomer in the P2 position, whereas in the P1 position, both *R* and *S* isomers were possible. Comparing the obtained activities of the investigated compounds **1**–**9** toward mammalian and plant aminopeptidases allows us to make some general observations. First of all, although there are no published results that suggested to not elongate the phosphonic inhibitors chain into the N-terminus site, the obtained compounds have presented the biological activity. Additionally, SsLAP has better tolerance for the residue in the P1 position, whereas HvLAP prefers hPhe residue in this position. The compounds containing hPhe residue have activity at least two times higher than analogs that possess Leu residue. It is in good agreement with the previous research, which showed that hPhePO_3_H_2_ is more than two times more active than LeuPO_2_H_3_ [48].

The best biological activity has been found for compound **3** toward SsLAP and for compound **1** toward HvLAP. It should be noticed that **3** is only 2.5 times weaker inhibitor of HvLAP than **1**. What is interesting, compound **3** has got relatively high activity despite that it contains Leu residue at the P1 position and not preferable hPhe. It can be suggested that the hydrophilic side chain of Ser residue in the P2 position is responsible for some additional intermolecular interactions, which improve the inhibitor activity. Additionally, a comparison of the inhibitory activity of compound **1** and phosphonic acid analog of hPhe toward barley aminopeptidase [48] shown that introduction of additional residues in position P1′ and P2 to a phosphonic acid analog of hPhe decreases the activity less than two times. Additionally, sequence **1** provides greater selectivity of HvLAP inhibition over SsLAP compared to the phosphine analog of homophenylleucine.

The introduction of the bulky residues, like biphenylalanine (Bip) or p-benzoyl _L-_phenylalanine (Bpa) in the position P2 instead of _L_-Met or _L_-Ser, have been showing that mammalian aminopeptidase has a much higher tolerance for the bigger side chain in this position than plant aminopeptidase. What is also interesting, it seems that these bulky residues improve the inhibitor activity towards SsLAP in comparison to the activity of inhibitors with _L_-Met in the P2 position. Comparing the activity of this group of inhibitors allows selecting compound **8** as the most potent inhibitor toward both enzymes. Whereas, the most selective is inhibitor **7**, which inhibits SsLAP almost 10 times stronger than HvLAP. This result is in full agreement with the structural preferences of HvLAP*,* which stronger interacts with the inhibitors containing hPhe than Leu in the P1 position [48].

### 2.3. Molecular Docking

Rational design and development of biologically active compounds require understanding the nature of inhibitor-enzyme interactions. Simulation of the docking process is one of the methods which might contribute to understanding the physical nature of those interactions, and thus, to understand the binding mode of the investigated compounds to the structure of selected aminopeptidases. Because there are no known crystal structures of porcine LAP and barley LAP, for simulation of docking process, the bovine lens LAP (BtLAP, *Bos Taurus* Lecine aminopeptidase) and tomato AP (SlLAP, *Solanum lycopersicum* Leucine aminopeptidase) have been selected, respectively, as a model of mammalian and plant enzymes. Taylor et al. showed that bovine LAP and porcine LAP share over 90% amino acid sequence homology [49,50]. Moreover, Grembecka et al. showed that the crystal structure of bovine lens LAP can be applicable to the design of new inhibitors of LAP from other organism tissues [4]. Furthermore, Wanat et al., as well as Janiszewska et al. showed that the known structure of tomato LAP is a good model of the barley seeds LAP [22,24]. All the above-mentioned observations make the use of enzymes from two different organisms for biological testing and molecular modeling highly justified. Because for the biological test, racemic mixture *SR* and *SS* isomers of **1**–**9** have been used, also during the simulations of the docking process, both types of isomers have been considered. Optimized structures of the investigated phosphinopeptides were obtained by using the Gaussian09 software at the B3LYP/6-311g(d,p) level with the PCM solvent model and the water selected as a solvent. Subsequently, obtained structures were docked to the active pocket of bovine lens leucyl aminopeptidase and tomato aminopeptidase available from Protein Data Bank (1LCP, 4KSI [51,52]), by using the Gold v2020.1 algorithm [53]. The structure of the enzymes was protonated at experimental pH using an H++ web server [54]. As the results of the docking simulation, for each inhibitor, three possible orientations of the docked molecule were obtained.

To validate chosen docking methodology redocking of LeuPO_3_H_2_ to the *BtLAP* has been performed. As shown with the obtained results, the used methodology led to the arrangement of the inhibitor in the enzyme active site similar to those from the X-ray structure (PDB: 1LCP) (see Figure S26). Applied molecular docking protocol allows to obtain the complexes of all investigated inhibitors **1**–**9** with both considered enzymes, and the best alignment of all investigated inhibitors in the active site of BtLAP and SlLAP are shown on the figures in the Supporting Information (see Figures S27–S30 and Table S3).

**Figure 5:**
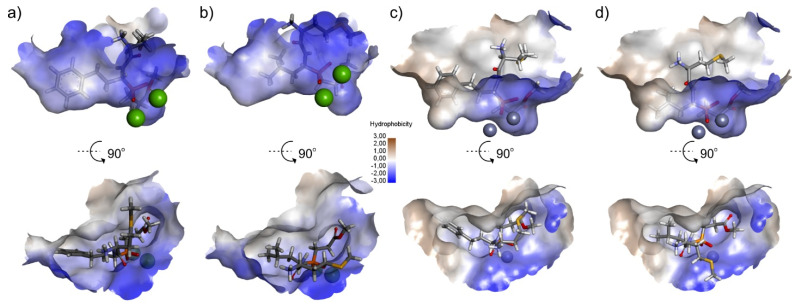
Alignment of (**a**) **1**-*SR* isomer and (**b**) **2**-*SR* isomer in the active site of SlLAP (PDB: 4KSI)*,* and (**c**) **1**-*SR* isomer and (**d**) **2**-*SR* isomer in the active site of BtLAP (PDB: 1LCP). Magnesium and zinc ions are shown, respectively, as the green and gray spheres. The surface of the active site is colored according to hydrophobicity.

To understand the influence of the P1 residue onto inhibitory activity toward mammalian leucyl aminopeptidase and plant aminopeptidase, the alignment of inhibitors **1** and **2** in the active sites of BtLAP and SlLAP have been compared. The importance of the inhibitory P1 residue is due to the interaction of its side chain with a cavity on the enzyme surface (called S1 pocket) located adjacent to the catalytic residues. As it is shown in Figure 5 in the case of the plant’s enzyme, if the designed inhibitor contains in the P1 position hPhe residue, the S1 pocket of SlLAP is completely occupied by its side chain. Whereas, if in the P1 position of the inhibitor is located Leu residue, the S1 pocket is not fulfilled completely, and bonded molecule still has some flexibility, which could result in lower affinity. In the case of alignment of **1** and **2** in the active site of BtLAP, no significant difference in the fulfilling of the S1 binding pocket could be observed. Such observation let to explains the higher tolerance of mammalian leucyl aminopeptidase for variation of the size of the side chain in the P1 position.

**Figure 6:**
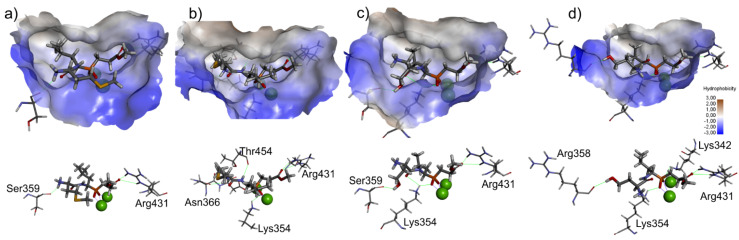
Alignment of (**a**) **2**-*SR* isomer, (**b**) **2**-*SS* isomer, (**c**) **3**-*SR* isomer, (**d**) **3**-*SS* isomer in the active site of *SlAP* (PDB: 4KSI). Magnesium ions are shown as a green sphere. Green lines represent intermolecular hydrogen bonds; blue lines represent ligand–metal interactions. The surface of the active site is colored according to hydrophobicity.

The results of the molecular modeling help to understand the phenomena of relatively high activity of **3** toward HvLAP. As it has already been noticed, the biological activity of the investigated compounds **1**–**9**, strongly depend on the type of the amino acid residue in position P1, the higher affinity has been found for compounds containing aromatic residue in this position—**1**, **4**, **6**, **8**. However, inhibitor **3,** although it contains in this critical position leucine residue, inhibits the HvLAP only three times weaker than **1**. We believe that this activity is the results of the introduction of _L_-Ser into position P2. Presence of the additional hydroxylic group in the serine side chain is probably responsible for the generation of additional intermolecular hydrogen bond, which could increase intermolecular interaction and stabilize the alignment of the inhibitor in enzyme active site. In fact, performed docking simulation, showed the presence of the additional hydrogen bond for the complex of **3** with plant aminopeptidase SlLAP comparison to complex of **2**-SlLAP. As it is shown in Figure 6, except for hydrogen bonds between: Side chain amine group of Lys354 and phosphine group of **3**; guanidine moiety of Arg431 and C-terminal carboxylic group of **3** and interaction between phosphinic moiety and magnesium ions, which could also be found in SlLAP*-***2** complex, the arrangement of **3** in the SlLAP active site is stabilized by one additional hydrogen bond between a hydroxylic group of serine and carboxylic group of Ser359 or carboxylic group of Arg358 for *SR*-isomer and *SS*-isomer, respectively.

**Figure 7:**
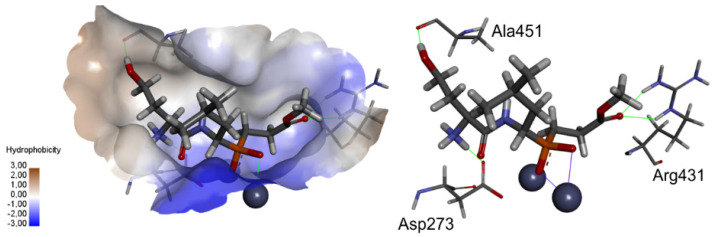
Modeled binding mode of **3**-SS isomer in the active center of bovine leucyl aminopeptidase (PDB: 1LCP). Amino acid residues of the inhibitor and enzyme are shown as sticks, while the zinc ions are shown as a gray sphere. Hydrogen bonds are shown as thin green lines, metal ion—phosphinic moiety interaction is shown as a thin blue line, while the surface of the active site is colored according to hydrophobicity.

Molecular modeling of the most active phosphinic compound **3** with di-zinc mammalian leucine aminopeptidase BtLAP revealed a typical mode of binding. The phosphinate group is involved in coordination with the catalytic metal ions (see Figure 7). C-terminal carboxylate is involved in an intermolecular hydrogen bond with the guanidine moiety of Arg431. The position of **3** is further stabilized by a salt bridge between the N-terminal amine group of **3** and the side chain carboxylic moiety of Asp273, as well as hydrogen bond between the side chain hydroxyl group of serine residue of **3** and carboxylic group of Ala451.

**Figure 8:**
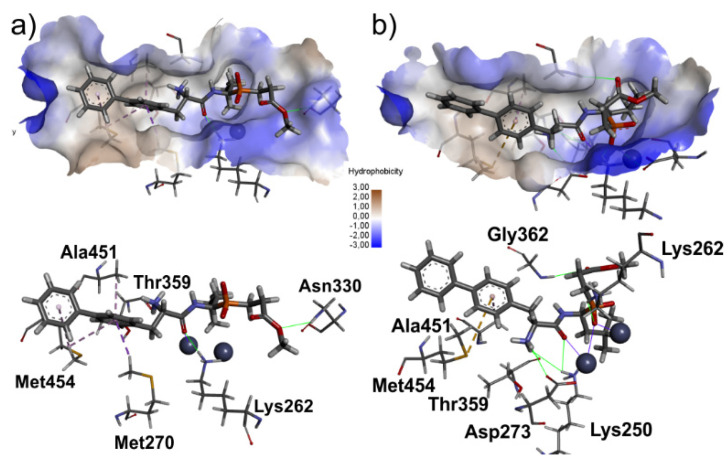
Modeled binding mode of (**a**) **7**-S*R* isomer and (**b**) **7**-*SS* isomer in the active center of bovine leucyl aminopeptidase (PDB: 1LCP). Amino acid residues of the inhibitor and enzyme are shown as sticks, while the zinc ions are shown as a gray sphere. Hydrogen bonds are shown as thin green lines, metal ion—coordinating moieties interaction shown as a thin blue line, aromatic π-electrons—aliphatic group, and sulfur atom shown as dashed lines. The surface of the active site is colored according to hydrophobicity.

In the case of inhibitors with aliphatic amino acids in position P2 (**1**–**5**), the typical binding of inhibitors in the active site of the enzyme was observed—coordination of catalytic metal ions by phosphinate and binding of the C-terminus of the inhibitor by hydrogen bonding to the guanidine moiety of Arg431 for SlLAP or Arg336 for BtLAP and interaction with the enzyme’s S1 cavity. For inhibitors **6**–**9,** containing large aromatic substituents in the P2 position, the situation is a bit more complicated. The conduced molecular modeling for compounds containing both types of isomer in position P1 show the different interaction of these inhibitors with the enzymatic active site, depending on the stereochemistry of P1 residues. This situation is presented in detail for **7** and BtLAP enzyme. The best pose found for the **7**-*SS* isomer showed almost typical binding mode for di-zinc leucine aminopeptidase (see Figure 8b), which means that zinc ions are coordinated by phosphinate. One of the oxygen atoms of the phosphinate moiety is involved in ionic interaction with NH_3_^+^ of Lys250 (2.45 Å). However, due to the presence of bulky biphenyl side chain at the P2 position, the inhibitor is shifted in the active site into S1′ direction. It results in the interaction of Leu residue from the P1 position with the S1′ binding pocket. The N-terminal amine group forms a salt bridge with Asp273 (2.42 Å) and a hydrogen bond with CO of Thr359 (2.43 Å). Moreover, the position of **7**-*SS* in the active pocket is stabilized by the interaction of π-electrons of biphenyl moiety with the aliphatic and sulfuric groups of Ala451 and Met454, respectively.

While the **7**-*SS* isomer shows an almost canonical binding to the BtLAP active site, the **7**-*SR* isomer shows an unusual way of binding (see Figure 8a). The most important difference is the coordination of the zinc ion by the amide CO of Bip residue instead of phosphinate. The orientation of this group is additionally stabilized by the hydrogen bonding to εNH_3_^+^ of Lys262 (2.60 Å). Moreover, in this case, the inhibitor molecule is shifted into S1′ direction, and leucine residue from the P1 position interacts with the enzyme’s binding pocket S1′. Inhibitor alignment in the active site is further stabilized by the series of π-alkyl interactions involving the biphenyl moiety of Bip and aliphatic side chains of Met270, Ala451, and Met454.

## 3. Materials and Methods

### 3.1. Chemistry

General information: Et_3_N was distilled and then dried over molecular sieves 70 Å. Hydrocynamaldehyde, isovaleraldehyde, and methyl acrylate were freshly distilled before use. EtOH, MeOH, CH_2_Cl_2_, CHCl_3_, THF, and DMF were freshly distilled and dried over molecular sieves 70 Å. All other solvents and reagents were obtained from commercial sources (Sigma-Aldrich, Madrid, Spain) and used without further purification. All reactions were performed under an atmosphere of dry nitrogen. Melting points were obtained using a Büchi Melting Point B-540 apparatus and are uncorrected. High-resolution mass spectra (HRMS) were measured on a Agilent 6530 Accurate-Mass QTOF LC/MS (Santa Clara, CA, USA) by the CI method using a mass spectrometer Q-TOF. ^1^H (400 MHz), ^13^C (100 MHz), and ^31^P NMR (160 MHz) spectra were recorded on a Bruker Avance 400 (Bruker BioSpin GmbH, Rheinstetten, Germany) spectrometer (see Figures S1–S25), respectively, in CDCl_3_, DMSO-*d6*, CD_3_OD, or D_2_O. Chemical shifts (*δ*_H_) are reported in parts per million (ppm). All coupling constants (*J*) values are given in Hz. Chemical shifts (*δ*_C_) are reported in parts per million (ppm) in a broadband decoupled mode. Flash-column chromatography was performed using commercial grades of silica gel finer than 230 mesh. Analytical thin layer chromatography was performed on precoated Merck silica gel 60 F_254_ plates, and spot visualization was accomplished by UV light (254 nm) or KMnO_4_ solution.

#### 3.1.1. [1-(N-Benzyloxycarbonylamino)-3-phenylpropyl]phosphinic Acid (**12**)

The phosphinic analog of homophenylalanine was synthesized in the reaction of hydrocynnamaldehyde with diphenylmethylamine and hypophosphorous acid, followed by hydrolysis of the adduct and *N*-terminal protection with benzyl chloroformate, similarly as described in the literature [2,55]. Global yield from three steps: 43%; mp 137–141 °C (lit. mp 142–143 °C [55]); ^1^H NMR (400 MHz, DMSO-*d6*) *δ* 7.72 (d, ^2^*J*_PH_ = 8.8 Hz, 1H, O*H*), 7.39–7.16 (m, 10H), 6.77 (d, ^1^*J*_PH_ = 531.6 Hz, 1H, P*H*), 5.07 (s, 2H, PhC*H_2_*O), 3.56–3.51 (m, 1H, C*H*-P), 2.76–2.53 (m, 2H, CH_2_C*H_2_*Ph), 1.92–1.77 (m, 2H, C*H_2_*CH_2_Ph); ^13^C NMR (100 MHz, DMSO-*d6*) *δ* 156.2 (d, ^3^*J*_PC_ = 3.2 Hz), 141.1, 137.0, 128.4, 128.4, 128.3, 127.9, 127.7, 125.9, 65.6, 49.9 (d, ^1^*J*_PC_ = 105.1 Hz), 31.4 (d, ^2^*J*_PC_ = 12.4 Hz), 28.3 (d, ^3^*J*_PC_ = 3.9 Hz); ^31^P NMR (160 MHz, DMSO-*d6*) *δ* 28.4; ESI-HRMS *m*/*z* calcd. for C_17_H_20_NNaO_4_P ([M+Na]^+^) 356.1028, found 356.1030.

#### 3.1.2. [1-(N-Benzyloxycarbonylamino)-3-methylbutyl]phosphinic Acid (**13**)

The phosphinic analog of leucine was synthesized in the reaction of isovaleraldehyde with diphenylmethylamine and hypophosphorous acid, followed by hydrolysis of the adduct and *N*-terminal protection with benzyl chloroformate, similarly as described in the literature [55]. Global yield from three steps: 46%; mp 149–151 °C (lit. mp 150–152 °C [2]); ^1^H NMR (400 MHz, DMSO-*d6*) *δ* 7.58 (d, ^2^*J*_PH_ = 9.0 Hz, 1H, O*H*), 7.39–7.15 (m, 5H), 6.74 (d, ^1^*J*_PH_ = 529.1 Hz, 1H, P*H*), 5.04 (s, 2H, PhC*H_2_*O), 3.69–3.60 (m, 1H, C*H*-P), 1.69–1.61 (m, 1H, CH_2_C*H* of *i*Bu group), 1.56–1.32 (m, 2H, C*H_2_*CH of *i*Bu group), 0.89 (d, ^3^*J*_HH_ = 6.7 Hz, 3H), 0.83 (d, ^3^*J*_HH_ = 6.6 Hz, 3H); ^13^C NMR (100 MHz, DMSO-*d6*) *δ* 156.2 (d, ^3^*J*_PC_ = 3.4 Hz), 137.0, 128.3, 127.8, 127.6, 65.6, 48.7 (d, ^1^*J*_PC_ = 105.5 Hz), 34.9 (d, ^2^*J*_PC_ = 3.6 Hz), 23.9 (d, ^3^*J*_PC_ = 12.2 Hz), 23.1, 21.0; ^31^P NMR (160 MHz, DMSO-*d6*) *δ* 29.1; ESI-HRMS *m*/*z* calcd. for C_13_H_20_NNaO_4_P ([M+Na]^+^) 308.1028, found 308.1025.

#### 3.1.3. General Procedure for the Synthesis of **14** and **15**

To a refluxing solution of compound **14** or **15** and 1-adamantyl bromide (1.5 equiv) in CHCl_3_ (10 mL/mmol) was added silver(I) oxide (1.2 equiv) portionwise over 1 h. After the solution was refluxed for 3 h, the solvent was removed in vacuo, and the residue was treated with Et_2_O (5 mL/mmol). The resulting mixture was filtered through a sintered glass vacuum filtration funnel with Celite, and the filtrates were evaporated. The residue was purified by column chromatography (SiO_2_, AcOEt/hexanes 1:1) to yield the desired compounds.

##### Methyl 3-[1-N-(benzyloxycarbonylamino)-3-phenylpropyl]hydroxyphosphinyl propanoate (**14**)

(9.0 g, 74%) obtained as a white solid from phosphinic acid **12** (9.66 g, 29 mmol), hexamethyldisilazane (30.85 mL, 145 mmol) and methyl acrylate (3.96 mL, 43.5 mmol) as described in the general procedure. The crude product was worked up with Et2O (120 mL/mmol) and left overnight for crystallization to give the title compound. mp: 163–166 °C; ^1^H NMR (400 MHz, DMSO-d6) δ 7.67 (d, ^2^*J*_PH_ = 9.4 Hz, 1H, OH), 7.58–7.14 (m, 10H), 5.08 (AB system, *J* = 12.6 Hz, 2H, PhCH_2_O), 4.08 ^(bs, 1H, N*H*), 3.74–3.54 (m, 1H,^ CH-P^), 3.59 (s, 3H), 2.76–2.36 (m, 4H, PCH_2_C*H_2_*, CH_2_C*H_2_*Ph), 2.01–1.60 (m, 4H, PC*H_2_*CH_2_, C*H_2_*CH_2_Ph)^; ^13^C NMR (100 MHz, DMSO-d6) δ 172.6 (d, ^3^*J*_PC_ = 15.8 Hz), 156.3 (d, ^3^*J*_PC_ = 3.6 Hz), 141.2, 137.2, 128.4, 128.4, 128.3, 127.8, 127.6, 125.9, 65.6, 51.6, 49.4 (d, ^1^*J*_PC_ = 106.2 Hz), 31.6 (d, ^2^*J*_PC_ = 11.8 Hz), 29.1, 26.1, 21.7 (d, ^1^*J*_PC_ = 89.5 Hz); ^31^P NMR (160 MHz, DMSO-d6) δ 46.2; ESI-HRMS *m*/*z* calcd. for C_21_H_27_NO_6_P ([M+H]^+^) 420.1576, found 420.1579.

##### Methyl 3-[1-N-(benzyloxycarbonylamino)-3-methylbutyl]hydroxyphosphinyl propanoate (**15**)

The phosphinic dipeptide was obtained according to the procedures previously described in the literature [18], and similar as described in the general procedure above. Yield: 66%; mp 150–155 °C (lit. mp 146–153 °C [18]); ^1^H NMR (400 MHz, DMSO-*d6*) *δ* 7.54 (d, ^2^*J*_PH_ = 9.4 Hz, 1H), 7.37–7.29 (m, 5H), 5.05 (AB system, *J* = 12.7 Hz, 2H, PhC*H_2_*O), 3.77–3.70 (m, 1H, C*H*-P), 3.59 (s, 3H), 2.56–2.40 (m, 2H, PCH_2_C*H_2_*), 1.85–1.78 (m, 2H, PC*H_2_*CH_2_), 1.63–1.38 (m, 3H, C*H*C*H_2_* of *^i^*Bu group), 0.89 (d, ^3^*J*_HH_ = 6.5 Hz, 3H), 0.81 (d, ^3^*J*_HH_ = 6.4 Hz, 3H); ^13^C NMR (100 MHz, DMSO-*d6*) *δ* 172.6 (d, ^3^*J*_PC_ = 15.7 Hz), 156.3 (d, ^3^*J*_PC_ = 3.8 Hz), 137.2, 128.3, 127.8, 127.5, 65.5, 51.6, 48.2 (d, ^1^*J*_PC_ = 106.9 Hz), 35.6, 26.2 (d, ^2^*J*_PC_ = 2.3 Hz), 24.0 (d, ^3^*J*_PC_ = 11.3 Hz), 23.3, 21.5 (d, ^1^*J*_PC_ = 89.5 Hz), 20.8; ^31^P NMR (160 MHz, DMSO-*d6*) *δ* 46.6; ESI-HRMS *m*/*z* calcd. for C_17_H_27_NO_6_P ([M+H]^+^) 372.1576, found 372.1585.

#### 3.1.4. General Procedure for the Protection of Hydroxyphosphinyl Group in **14** and **15**

To a refluxing solution of compound **14** or **15** and 1-adamantyl bromide (1.5 equiv) in CHCl_3_ (10 mL/mmol) was added silver(I) oxide (1.2 equiv) portionwise over 1 h. After the solution was refluxed for 3 h, the solvent was removed in vacuo, and the residue was treated with Et_2_O (5 mL/mmol). The resulting mixture was filtered through a sintered glass vacuum filtration funnel with Celite, and the filtrates were evaporated. The residue was purified by column chromatography (SiO_2_, AcOEt/hexanes 1:1) to yield the desired compounds.

##### Methyl 3-[(1-N-(benzyloxycarbonylamino)-3-phenylpropyl)adamantyloxyphoshinyl]propanoate (**16**)

(9.60 g, 87%) obtained as a white solid from compound **14** (8.38 g, 20 mmol), 1-adamantyl bromide (6.52 g, 30 mmol) and silver(I) oxide (5.56 g, 24 mmol) as described in the general procedure. The residue was purified by column chromatography eluting from (SiO_2_, hexanes) to (SiO_2_, AcOEt/hexanes 1:1) to afford the title compound. mp: 114–116 °C; ^1^H NMR (400 MHz, CDCl_3_) *δ* 7.36–7.07 (m, 10H), 5.45 (d, ^3^*J*_PH_ = 10.3 Hz, 1H, N*H*)_major_, 5.10 (AB system, *J* = 12.3 Hz, 2H, PhC*H_2_*O), 4.96 (d, ^3^*J*_PH_ = 10.3 Hz, 1H, N*H*)_minor_, 3.95–3.90 (m, 1H, C*H*-P), 3.64 (s, 3H), 2.83–2.45 (m, 4H, PCH_2_C*H_2_*, CH_2_C*H_2_*Ph), 2.18–1.88 (m, 13H, CC*H_2_* and C*H*CH_2_ of Ad group, C*H_2_*CH_2_Ph, PC*H_2_*CH_2_), 1.63–1.57 (m, 6H, CHC*H_2_*CH of Ad group); ^13^C NMR (100 MHz, CDCl_3_) *δ* 172.9 (d, ^3^*J*_PC_ = 16.9 Hz), 172.8 (d, ^3^*J*_PC_ = 17.7 Hz), 156.4 (d, ^3^*J*_PC_ = 5.6 Hz)_major_, 156.1 (d, ^3^*J*_PC_ = 4.8 Hz)_minor_, 141.1_major_, 140.9_minor_, 136.4_major_, 136.1_minor_, 129.3, 128.5, 128.5, 128.4, 128.3, 128.2, 128.1, 128.0, 127.8, 126.2, 126.0, 125.9, 83.3 (d, ^2^*J*_PC_ = 9.9 Hz)_major_, 83.0 (d, ^2^*J*_PC_ = 10.0 Hz)_minor_, 67.2_minor_, 67.0_major_, 51.9_minor_, 51.8_major_, 49.5 (d, ^1^*J*_PC_ = 110.8 Hz), 44.4 (d, ^3^*J*_PC_ = 3.3 Hz)_minor_, 44.3 (d, ^3^*J*_PC_ = 3.3 Hz)_major_, 35.6, 32.1 (d, ^2^*J*_PC_ = 11.2 Hz), 31.1, 29.6, 27.2, 23.6 (d, ^1^*J*_PC_ = 90.0 Hz)_minor_, 23.5 (d, ^1^*J*_PC_ = 89.9 Hz)_major_; ^31^P NMR (160 MHz, CDCl_3_) *δ* 48.4_minor_, 47.7_major_; ESI-HRMS *m*/*z* calcd. for C_31_H_40_NNaO_6_P ([M+Na]^+^) 576.2491, found 576.2496.

##### Methyl 3-[(1-N-(benzyloxycarbonylamino)-3-methylbutyl)adamantyloxyphoshinyl]propanoate (**17**)

(8.19 g, 90%) obtained as a white solid from compound **15** (6.68 g, 18 mmol), 1-adamantyl bromide (5.87 g, 27 mmol) and silver(I) oxide (5.01 g, 21.6 mmol) as described in the general procedure. The residue was purified by column chromatography eluting from (SiO_2_, hexanes) to (SiO_2_, AcOEt/hexanes 1:1) to afford the title compound. mp: 111–115 °C; ^1^H NMR (400 MHz, CDCl_3_) *δ* 7.35–7.26 (m, 5H), 5.29 (bs, 1H, N*H*), 5.08 (AB system, *J* = 12.4 Hz, 2H, PhC*H_2_*O)_major_, 5.08 (AB system, *J* = 12.1 Hz, 2H, PhC*H_2_*O)_minor_, 4.84 (bs, 1H, N*H*), 3.99–3.90 (m, 1H, C*H*-P), 3.66 (s, 3H)_minor_, 3.64 (s, 3H)_major_, 2.70–2.44 (m, 2H, PCH_2_C*H_2_*), 2.14–1.93 (m, 9H, CC*H_2_* and C*H*CH_2_ of Ad group), 1.73–1.37 (m, 11H, CHC*H_2_*CH of Ad group, C*H*C*H_2_* of *^i^*Bu group, PC*H_2_*CH_2_), 0.91 (d, ^3^*J*_HH_ = 6.4 Hz, 3H), 0.89 (d, ^3^*J*_HH_ = 6.8 Hz, 3H); ^13^C NMR (100 MHz, CDCl_3_) *δ* 172.9 (d, ^3^*J*_PC_ = 16.8 Hz)_major_, 172.8 (d, ^3^*J*_PC_ = 17.8 Hz)_minor_, 156.2 (d, ^3^*J*_PC_ = 5.1 Hz)_major_, 156.0 (d, ^3^*J*_PC_ = 4.5 Hz)_minor_, 136.4_major_, 136.2_minor_, 128.5_major_, 128.4_minor_, 128.2_minor_, 128.1_major_, 128.0_minor_, 127.9_major_, 83.0 (d, ^2^*J*_PC_ = 10.0 Hz)_major_, 82.7 (d, ^2^*J*_PC_ = 10.0 Hz)_minor_, 67.1_minor_, 67.0_major_, 51.9_minor_, 51.8_major_, 48.1 (d, ^1^*J*_PC_ = 110.9 Hz)_major_, 48.0 (d, ^1^*J*_PC_ = 105.6 Hz)_minor_, 44.5 (d, ^3^*J*_PC_ = 3.3 Hz)_minor_, 44.3 (d, ^3^*J*_PC_ = 3.2 Hz)_major_, 37.1_minor_, 36.7_major_, 35.6_major_, 35.6_minor_, 31.1, 27.2 (d, ^2^*J*_PC_ = 1.1 Hz), 24.5 (d, ^3^*J*_PC_ = 11.7 Hz)_minor_, 24.4 (d, ^3^*J*_PC_ = 10.9 Hz)_major_, 23.7 (d, ^1^*J*_PC_ = 89.5 Hz)_minor_, 23.5, 23.4, 23.4 (d, ^1^*J*_PC_ = 89.9 Hz)_major_, 21.1; ^31^P NMR (160 MHz, CDCl_3_) *δ* 49.1, 48.5; ESI-HRMS *m*/*z* calcd. for C_27_H_40_NNaO_6_P ([M+Na]^+^) 528.2491, found 528.2497.

#### 3.1.5. General Procedure for the Coupling Reaction of Building Blocks **16** and **17** Involving HOBt and EDC

The N-(benzyloxycarbonyl) compounds **16** or **17** were N-deprotected by dissolving in MeOH (15 mL/mmol) and stirring with 10% Pd/C catalyst (100 mg/mmol) under an atmosphere of hydrogen 20 psi overnight at room temperature. The mixture was filtered through a sintered glass vacuum filtration funnel with Celite, washed several times with MeOH, and filtrate concentrated under reduced pressure. The residue and the corresponding N-Boc-protected amino acid (1 equiv) were dissolved in DMF (5 mL/mmol) and stirred at room temperature for 20 min with N-hydroxybenzotriazole (HOBt) (1.1 equiv) and N-(dimethylaminopropyl)-N’-ethylcarbodiimide hydrochloride (EDC·HCl) (1.1 equiv). After adding Et_3_N (1.5 equiv), the suspension was stirred for 20 h at room temperature. Then 10% aqueous solution of citric acid (50 mL/mmol) was added, and the mixture was extracted with AcOEt (3 × 20 mL/mmol). The combined organic layer was washed with 10% aqueous solution of citric acid (2 × 20 mL/mmol), 0.5 M NaHCO_3_ (2 × 20 mL/mmol), and saturated brine (1 × 20 mL/mmol). The organic layer was dried over MgSO_4_, filtrated, and concentrated to dryness in a vacuum.

##### Methyl 3-[(adamantan-1-yloxy)-(1-((S)-2-(tert-butoxycarbonylamino)-4-(methylthio)butan-amido)-3-phenylpropyl)phosphinyl]propanoate (**20**)

(2.41 g, 60%) obtained as a foamy white solid from *N*-benzyloxycarbonyl compound **16** (3.42 g, 6.18 mmol) and 10% Pd/C catalyst (618 mg) for the deprotection step, followed by coupling reaction using *N*-Boc-methionine (1.54 g, 6.18 mmol), as described in the general procedure. The crude product was purified by flash-chromatography eluting from (SiO_2_, AcOEt/hexanes 1:6) to (SiO_2_, AcOEt/hexanes 1:1) to afford phosphinotripeptide **20**. ^1^H NMR (400 MHz, CDCl_3_) *δ* 7.50–7.44 (m, 1H, N*H*), 7.24–7.08 (m, 5H), 5.73 and 5.40 (bs, 1H, N*H*), 4.31–4.19 (m, 2H, NHC*H*CO, C*H*-P), 3.64 (s, 3H)_major_, 3.63 (s, 3H)_minor_, 2.77–2.32 (m, 6H, PCH_2_C*H_2_*, CH_2_C*H_2_*Ph, CH_2_C*H_2_*S), 2.12–1.81 (m, 18H, CC*H_2_* and C*H*CH_2_ of Ad group, C*H_2_*CH_2_Ph, PC*H_2_*CH_2_, C*H_2_*CH_2_SC*H_3_*), 1.67–1.51 (m, 6H, CHC*H_2_*CH of Ad group), 1.37 (s, 9H)_major_, 1.36 (s, 9H)_minor_; ^13^C NMR (100 MHz, CDCl_3_) *δ* 172.7 (d, ^3^*J*_PC_ = 16.5 Hz)_minor_, 172.6 (d, ^3^*J*_PC_ = 17.3 Hz)_major_, 172.3 (d, ^3^*J*_PC_ = 11.2 Hz)_minor_, 172.3 (d, ^3^*J*_PC_ = 11.7 Hz)_major_, 155.4_minor_, 155.3_major_, 141.0_minor_, 140.8_major_, 128.4, 128.4, 128.3, 128.3, 126.0_major_, 125.9_minor_, 83.5 (d, ^2^*J*_PC_ = 10.2 Hz)_minor_, 83.4 (d, ^2^*J*_PC_ = 10.3 Hz)_major_, 79.8, 54.4_minor_, 54.0_major_, 51.9_major_, 51.8_minor_, 47.1 (d, ^1^*J*_PC_ = 111.1 Hz)_minor_, 47.0 (d, ^1^*J*_PC_ = 111.2 Hz)_major_, 44.3 (d, ^3^*J*_PC_ = 3.5 Hz), 35.5, 32.2, 32.0 (d, ^2^*J*_PC_ = 11.4 Hz), 31.1, 30.1, 29.4, 28.2, 27.3, 23.6 (d, ^1^*J*_PC_ = 90.4 Hz), 15.3_minor_, 15.2_major_; ^31^P NMR (160 MHz, CDCl_3_) *δ* 48.5, 48.4; ESI-HRMS *m*/*z* calcd. for C_33_H_51_N_2_NaO_7_PS ([M+Na]^+^) 673.3052, found 673.3045.

##### Methyl 3-[(adamantan-1-yloxy)(1-((S)-2-(tert-butoxycarbonylamino)-4-(methylthio)butan-amido)-3-methylbutyl)phosphinyl]propanoate (**21**)

(907 mg, 70%) obtained as a foamy white solid from N-benzyloxycarbonyl compound **17** (1.09 g, 2.15 mmol) and 10% Pd/C catalyst (215 mg) for the deprotection step, followed by coupling reaction using N-Boc-methionine (0.54 g, 2.15 mmol), as described in the general procedure. The crude product was purified by flash-chromatography eluting from (SiO_2_, AcOEt/hexanes 1:5) to (SiO_2_, AcOEt/hexanes 4:1) to afford phosphinotripeptide **21**. ^1^H NMR (400 MHz, CDCl_3_) δ 4.30–4.25 (m, 2H, CH-P, NHCHCO), 3.65 (s, 3H)_major_, 3.64 (s, 3H)_minor_, 2.66–2.46 (m, 4H, PCH_2_CH_2_, CH_2_CH_2_S), 2.15–1.98 (m, 14H, CCH_2_ and CHCH_2_ of Ad group, CH_2_CH_2_SCH_3_), 1.87–1.84 (m, 2H, PCH_2_CH_2_), 1.60–1.40 (m, 9H, CHCH_2_CH of Ad group, CHCH_2_ of ^i^Bu group), 1.38 (s, 9H), 0.90 (d, ^3^*J*_HH_ = 6.1 Hz, 3H), 0.84 (d, ^3^*J*_HH_ = 6.2 Hz, 3H); ^13^C NMR (100 MHz, CDCl_3_) δ 172.7 (d, ^3^*J*_PC_ = 17.4 Hz), 171.9, 155.3, 83.3 (d, ^2^*J*_PC_ = 10.1 Hz), 79.8, 53.9, 51.9, 45.6 (d, ^1^*J*_PC_ = 111.5 Hz), 44.3, (d, ^3^*J*_PC_ = 3.6 Hz), 36.3, 35.6, 32.1, 31.1, 30.1, 28.2, 27.3, (d, ^2^*J*_PC_ = 4.1 Hz), 24.4 (d, ^3^*J*_PC_ = 10.9 Hz), 23.4, 23.3 (d, ^1^*J*_PC_ = 90.5 Hz), 21.1, 15.3; ^31^P NMR (160 MHz, CDCl_3_) δ 49.3_minor_, 49.0_major_; ESI-HRMS *m*/*z* calcd. for C_29_H_51_N_2_NaO_7_PS ([M+Na]^+^) 625.3052, found 625.3045.

##### Methyl 3-[(adamantan-1-yloxy)(1-((S)-2-(tert-butoxycarbonylamino)-4-((tert-butyldime-thylsilyl)-oxy)butanamido)-3-methylbutyl)phosphinyl]propanoate (**22**)

(1.53 g, 51%) obtained as a white solid from N-benzyloxycarbonyl compound **17** (2.21 g, 4.37 mmol) and 10% Pd/C catalyst (437 mg) for the deprotection step, followed by coupling reaction using O-TBDMS-protected N-Boc-homoserine (Boc-_L_-Hse(TBDMS)-OH was prepared starting from _L_-Hse-OH through protection first the hydroxyl group as OTBDMS [39] followed by protection of the N-terminus as N-Boc protecting group [40]) (1.46 g, 4.37 mmol), as described in the general procedure. The crude product was purified by flash-chromatography eluting from 1:4 AcOEt/hexanes to AcOEt to afford phosphinotripeptide **22**. mp: 156–158 °C; ^1^H NMR (400 MHz, CDCl_3_) δ 4.43–4.18 (m, 2H, CH-P, NHCHCO), 3.75–3.68 (m, 2H, CH_2_-OSi), 3.64 (s, 3H)_major_, 3.63 (s, 3H)_minor_, 2.72–2.49 (m, 2H, PCH_2_CH_2_), 2.18–1.91 (m, 13H, CCH_2_ and CHCH_2_ of Ad group, PCH_2_CH_2_, CH_2_CH_2_OSi), 1.63–1.21 (m, 18H, CHCH_2_CH of Ad group, CHCH_2_ of ^i^Bu group, ^t^Bu of Boc protecting group), 0.92–0.82 (m, 6H, CH_2_CH(CH_3_)_2_), 0.86 (s, 9H, Si^t^Bu), 0.03 (s, 6H, Si(CH_3_)_2_)_major_, 0.02 (s, 6H, Si(CH_3_)_2_)_minor_; ^13^C NMR (100 MHz, CDCl_3_) δ 173.0 (d, ^3^*J*_PC_ = 16.6 Hz), 172.2_minor_, 172.0_major_, 155.8_major_, 155.4_minor_, 83.0 (d, ^2^*J*_PC_ = 9.9 Hz)_minor_, 82.9 (d, ^2^*J*_PC_ = 9.8 Hz)_major_, 80.5_minor_, 79.9_major_, 65.7, 55.2_minor_, 54.8_major_, 51.9_major_, 50.1_minor_, 45.6 (d, ^1^*J*_PC_ = 109.9 Hz), 44.3_major_, 44.3_minor_, 41.6_minor_, 41.5_major_, 36.2_major_, 36.1_minor_, 35.7, 31.1, 30.8, 28.2_major_, 28.1_minor_, 27.2_minor_, 27.1_major_, 25.8_minor_, 25.8_major_, 24.4 (d, ^3^*J*_PC_ = 10.9 Hz)_major_, 24.1 (d, ^3^*J*_PC_ = 10.9 Hz)_minor_, 23.5, 23.3 (d, ^1^*J*_PC_ = 90.1 Hz), 21.0_major_, 20.9_minor_, –5.6_minor_, –5.7_major_;^31^P NMR (160 MHz, CDCl_3_) δ 49.0_major_, 48.8_minor_; ESI-HRMS *m*/*z* calcd. for C_34_H_64_N_2_O_8_PSi ([M+H]^+^) 687.4170, found 687.4164.

##### Methyl 3-[(adamantan-1-yloxy)(1-((S)-2-(tert-butoxycarbonylamino)-4-(hydroxybutan-amido)-3-methylbutyl)phosphinyl]propanoate (**23**)

To a stirred 0 °C solution of phosphinotripeptide **22** (651 mg, 0.95 mmol) in THF (10 mL) was added a 1 M solution of tetrabutylammonium fluoride in THF (1.90 mL, 1.90 mmol). The solution was stirred for 5 min at 0 °C and then was allowed to warm to rt and was stirred for 20 h. The mixture was diluted with water (4 mL) and extracted with CH_2_Cl_2_ (3 × 4 mL). The combined organic layers were dried over MgSO_4_, and concentrated. The residue was worked up with hexanes/Et_2_O 1:1 and left overnight for crystallization to yield 384 mg (71%) of the title compound as a white solid. Due to the instability of this compound, it was subjected to deprotection of Boc and adamantyl groups without further purification steps. ^1^H NMR (400 MHz, CDCl_3_) *δ* 4.43–4.18 (m, 2H, C*H*-P, NHC*H*CO), 3.72–3.63 (m, 2H, C*H_2_*-OH), 3.66 (s, 3H)_minor_, 3.65 (s, 3H)_major_, 2.73–2.43 (m, 2H, PCH_2_C*H_2_*), 2.14–1.92 (m, 13H, CC*H_2_* and C*H*CH_2_ of Ad group, PC*H_2_*CH_2_, C*H_2_*CH_2_OH), 1.67–1.32 (m, 18H, CHC*H_2_*CH of Ad group, C*H*C*H_2_* of *^i^*Bu group, *^t^*Bu), 0.92–0.82 (m, 6H, CH_2_CH(C*H_3_*)_2_); ^31^P NMR (160 MHz, CDCl_3_) *δ* 50.0, 49.9; ESI-HRMS *m*/*z* calcd. for C_28_H_50_N_2_O_8_P ([M+H]^+^) 573.3305, found 573.3297.

##### Methyl 3-[(adamantan-1′-yloxy)-((1-((S)-3-([1,1′-biphenyl]-4-yl)-2-(tert-butoxycarbonyl-amino)propanamido)-3-phenylpropyl)phosphinyl]propanoate (**26**)

(1.05 g, 76%) obtained as a foamy white solid from *N*-benzyloxycarbonyl compound **16** (1.03 g, 1.87 mmol) and 10% Pd/C catalyst (187 mg) for the deprotection step, followed by coupling reaction using *N*-Boc-_L_-4,4′-biphenylalanine (660 mg, 1.87 mmol), as described in the general procedure. The crude product was purified by flash-chromatography eluting from (SiO_2_, AcOEt/hexanes 1:6) to (SiO_2_, AcOEt) to afford phosphinotripeptide **26**. ^1^H NMR (400 MHz, CDCl_3_) *δ* 7.55–7.05 (m, 14H), 4.52–4.21 (m, 2H, C*H*-P, NHC*H*CO), 3.65 (s, 3H)_major_, 3.61 (s, 3H)_minor_, 3.21–2.99 (m, 2H, C*H_2_*-biphenyl), 2.71–2.40 (m, 4H, PCH_2_C*H_2_*, CH_2_C*H_2_*Ph), 2.11–1.73 (m, 13H, CC*H_2_* and C*H*CH_2_ of Ad group, C*H_2_*CH_2_Ph, PC*H_2_*CH_2_), 1.62–1.46 (m, 6H, CHC*H_2_*CH of Ad group), 1.39 (s, 9H)_minor_, 1.36 (s, 9H)_major_; ^13^C NMR (100 MHz, CDCl_3_) *δ* 172.8 (d, ^3^*J*_PC_ = 17.0 Hz)_minor_, 172.7 (d, ^3^*J*_PC_ = 16.8 Hz)_major_, 171.0 (d, ^3^*J*_PC_ = 11.2 Hz)_minor_, 170.9 (d, ^3^*J*_PC_ = 11.4 Hz)_major_, 155.5_minor_, 155.3_major_, 141.1, 140.9, 140.7, 140.6, 140.4, 140.4, 139.8, 139.8, 135.6, 135.6, 135.5, 135.4, 129.7, 129.3, 128.7, 128.7, 128.6, 128.5, 128.5, 128.4, 128.4, 128.3, 128.3, 128.2, 127.4, 127.3, 127.3, 127.3, 127.2, 127.1, 126.9, 126.9, 126.8, 126.8, 126.0, 125.9, 83.5 (d, ^2^*J*_PC_ = 10.1 Hz)_major_, 83.0 (d, ^2^*J*_PC_ = 10.1 Hz)_minor_, 80.4_minor_, 80.2_major_, 56.0_major_, 55.7_minor_, 52.0_minor_, 51.9_major_, 47.3 (d, ^1^*J*_PC_ = 110.0 Hz)_minor_, 47.2 (d, ^1^*J*_PC_ = 110.0 Hz)_major_, 44.4 (d, ^3^*J*_PC_ = 4.1 Hz)_minor_, 44.3 (d, ^3^*J*_PC_ = 3.2 Hz)_major_, 37.7_minor_, 37.3_major_, 35.5_major_, 35.4_minor_, 32.2 (d, ^2^*J*_PC_ = 11.6 Hz)_major_, 32.0 (d, ^2^*J*_PC_ = 11.5 Hz)_minor_, 31.1_major_, 31.0_minor_, 29.4, 28.2_minor_, 28.1_major_, 23.6 (d, ^1^*J*_PC_ = 90.7 Hz)_minor_, 23.4 (d, ^1^*J*_PC_ = 90.0 Hz)_major_; ^31^P NMR (160 MHz, CDCl_3_) *δ* 48.0_major_, 47.9_minor_; ESI-HRMS *m*/*z* calcd. for C_43_H_56_N_2_O_7_P ([M+H]^+^) 743.3825, found 743.3824.

##### Methyl 3-[(adamantan-1-yloxy)-((1-((S)-3-([1,1′-biphenyl]-4-yl)-2-(tert-butoxycarbonyl-amino)propanamido)-3-methylbutyl)phosphinyl]propanoate (**27**)

(826 mg, 50%) obtained as a foamy white solid from *N*-benzyloxycarbonyl compound **17** (1.20 g, 2.38 mmol) and 10% Pd/C catalyst (238 mg) for the deprotection step, followed by coupling reaction using *N*-Boc-_L_-4,4′-biphenylalanine (812 mg, 2.38 mmol), as described in the general procedure. The crude product was purified by flash-chromatography eluting from (SiO_2_, AcOEt/hexanes 1:4) to (SiO_2_, AcOEt/hexanes 3:1) to afford phosphinotripeptide **27**. ^1^H NMR (400 MHz, CDCl_3_) *δ* 7.57–7.25 (m, 9H), 4.46–4.21 (m, 2H, C*H*-P, NHC*H*CO), 3.66 (s, 3H)_major_, 3.61 (s, 3H)_minor_, 3.18–3.00 (m, 2H, C*H_2_*-biphenyl), 2.67–2.44 (m, 2H, PCH_2_C*H_2_*), 2.14–1.90 (m, 11H, CC*H_2_* and C*H*CH_2_ of Ad group, PC*H_2_*CH_2_), 1.63–1.43 (m, 9H, CHC*H_2_*CH of Ad group, C*H*C*H_2_* of *^i^*Bu group), 1.40 (s, 9H)_minor_, 1.38 (s, 9H)_major_, 0.88 (d, ^3^*J*_HH_ = 6.3 Hz, 3H), 0.81 (d, ^3^*J*_HH_ = 6.2 Hz, 3H); ^13^C NMR (100 MHz, CDCl_3_) *δ* 172.8 (d, ^3^*J*_PC_ = 18.5 Hz)_minor_, 172.8 (d, ^3^*J*_PC_ = 17.0 Hz)_major_, 170.8_major_, 170.7_minor_, 155.5, 155.2, 140.7, 140.7, 140.5, 140.4, 139.7, 139.6, 135.6, 135.6, 135.5, 129.7, 129.7, 129.6, 128.7, 128.7, 128.7, 127.3, 127.2, 127.1, 126.9, 126.9, 126.8, 126.8, 83.2 (d, ^2^*J*_PC_ = 10.2 Hz)_major_, 82.8 (d, ^2^*J*_PC_ = 10.1 Hz)_minor_, 80.1_major_, 79.9_minor_, 55.8_major_, 55.6_minor_, 51.9_major_, 51.8_minor_, 45.7 (d, ^1^*J*_PC_ = 110.7 Hz)_minor_, 45.7 (d, ^1^*J*_PC_ = 110.7 Hz)_major_, 44.4_minor_, 44.3_major_, 37.6_minor_, 37.3_major_, 36.2_minor_, 35.9_major_, 35.6_major_, 35.5_minor_, 31.1_major_, 31.0_minor_, 28.2_major_, 28.1_minor_, 27.1 (d, ^2^*J*_PC_ = 30.7 Hz)_major_, 27.0 (d, ^2^*J*_PC_ = 28.2 Hz)_minor_, 24.3, 24.1, 23.4 (d, ^1^*J*_PC_ = 89.6 Hz)_minor_, 23.4_major_, 23.2 (d, ^1^*J*_PC_ = 89.1 Hz)_major_, 21.1_minor_; ^31^P NMR (160 MHz, CDCl_3_) *δ* 48.6_minor_, 48.5_major_; ESI-HRMS *m*/*z* calcd. for C_39_H_56_N_2_O_7_P ([M+H]^+^) 695.3825, found 695.3809.

##### Methyl 3-[(adamantan-1-yloxy)-((1-((S)-3-(4-benzoylphenyl)-2-(tert-butoxycarbonyl-amino)-propanamido)-3-phenylpropyl)phosphinyl]propanoate (**28**)

(1.26 g, 66%) obtained as a foamy white solid from *N*-benzyloxycarbonyl compound **16** (1.37 g, 2.47 mmol) and 10% Pd/C catalyst (247 mg) for the deprotection step, followed by coupling reaction using *N*-Boc-*p*-benzoyl-_L_-phenylalanine (911 mg, 2.47 mmol), as described in the general procedure. The crude product was purified by flash-chromatography eluting from (SiO_2_, AcOEt/hexanes 1:3) to (SiO_2_, AcOEt/hexanes 4:1) to afford phosphinotripeptide **28**. ^1^H NMR (400 MHz, CDCl_3_) *δ* 7.75–7.08 (m, 14H), 4.44–4.22 (m, 2H, C*H*-P, NHC*H*CO), 3.65 (s, 3H), 3.63 (s, 3H), 3.31–3.07 (m, 2H, C*H_2_*-4-benzoylphenyl), 2.64–2.44 (m, 4H, PCH_2_C*H_2_*, CH_2_C*H_2_*Ph), 2.11–1.85 (m, 13H, CC*H_2_* and C*H*CH_2_ of Ad group, C*H_2_*CH_2_Ph, PC*H_2_*CH_2_), 1.62–1.57 (m, 6H, CHC*H_2_*CH of Ad group), 1.38 (s, 9H), 1.35 (s, 9H); ^13^C NMR (100 MHz, CDCl_3_) *δ* 196.2_minor_, 196.1_major_, 173.0 (d, ^3^*J*_PC_ = 14.1 Hz)_minor_, 172.7 (d, ^3^*J*_PC_ = 16.6 Hz)_major_, 171.4_major_, 170.7_minor_, 155.4_minor_, 155.3_major_, 141.9, 141.8, 141.7, 141.6, 141.0, 140.9, 140.7, 140.6, 137.5, 137.4, 136.2, 136.2, 136.1, 136.1, 132.4, 132.3, 132.2, 130.5, 130.5, 130.4, 130.4, 129.9, 129.9, 129.8, 129.3, 129.3, 129.2, 128.5, 128.4, 128.4, 128.4, 128.3, 128.2, 128.2, 126.1, 126.0, 83.5 (d, ^2^*J*_PC_ = 93.8 Hz), 83.4 (d, ^2^*J*_PC_ = 10.4 Hz), 80.3_major_, 80.2_minor_, 55.6_major_, 55.5_minor_, 52.0_minor_, 52.0_major_, 47.5 (d, ^1^*J*_PC_ = 104.4 Hz)_minor_, 47.4 (d, ^1^*J*_PC_ = 109.8 Hz)_major_, 44.4 (d, ^3^*J*_PC_ = 3.0 Hz)_minor_, 44.3 (d, ^3^*J*_PC_ = 3.0 Hz)_major_, 37.9, 37.6, 35.5, 35.5, 32.0 (d, ^2^*J*_PC_ = 11.6 Hz), 31.1_minor_, 31.1_major_, 29.5, 28.2_minor_, 28.1_major_, 27.1_major_, 26.8_minor_, 23.6 (d, ^1^*J*_PC_ = 90.8 Hz)_minor_, 23.4 (d, ^1^*J*_PC_ = 90.7 Hz)_major_; ^31^P NMR (160 MHz, CDCl_3_) *δ* 47.7, 47.6; ESI-HRMS *m*/*z* calcd. for C_44_H_56_N_2_O_8_P ([M+H]^+^) 771.3774, found 771.3786.

##### Methyl 3-[(adamantan-1-yloxy)-((1-((S)-3-(4-benzoylphenyl)-2-(tert-butoxycarbony)-amino)-propanamido)-3′-methylbutyl)phosphinyl]propanoate (**29**)

(923 mg, 51%) obtained as a foamy white solid from *N*-benzyloxycarbonyl compound **17** (1.26 g, 2.49 mmol) and 10% Pd/C catalyst (249 mg) for the deprotection step, followed by coupling reaction using *N*-Boc-*p*-benzoyl-_L_-phenylalanine (919 mg, 2.49 mmol), as described in the general procedure. The crude product was purified by flash-chromatography eluting from (SiO_2_, AcOEt/hexanes 1:4) to (SiO_2_, AcOEt/hexanes 3:1) to afford phosphinotripeptide **29**. ^1^H NMR (400 MHz, CDCl_3_) *δ* 7.77–7.31 (m, 9H), 4.44–4.24 (m, 2H, C*H*-P, NHC*H*CO), 3.65 (s, 3H), 3.64 (s, 3H), 3.24–3.01 (m, 2H, C*H_2_*-4-benzoylphenyl), 2.64–2.51 (m, 2H, PCH_2_C*H_2_*), 2.14–1.88 (m, 11H, CC*H_2_* and C*H*CH_2_ of Ad group, PC*H_2_*CH_2_), 1.62–1.41 (m, 9H, CHC*H_2_*CH of Ad group, C*H*C*H_2_* of *^i^*Bu group), 1.38 (s, 9H), 1.36 (s, 9H), 0.90–0.80 (m, 6H); ^13^C NMR (100 MHz, CDCl_3_) *δ* 196.2, 196.1, 172.9, 172.7, 170.6, 170.5, 155.5, 155.3, 141.9, 141.8, 141.7, 141.6, 137.6, 137.6, 137.5, 137.5, 136.3, 136.2, 136.1, 132.4, 132.4, 132.3, 130.5, 130.4, 130.4, 130.0, 129.9, 129.9, 129.3, 129.3, 129.2, 128.3, 128.2, 83.4, 82.7, 80.4, 80.3, 55.6, 55.5, 52.0, 51.9, 45.9 (d, ^1^*J*_PC_ = 105.8 Hz), 44.5, 44.4, 38.0, 37.6, 36.5, 36.0, 35.6, 31.1, 28.2, 27.1 (d, ^2^*J*_PC_ = 27.2 Hz), 24.4, 24.3, 23.7 (d, ^1^*J*_PC_ = 90.0 Hz), 23.6 (d, ^1^*J*_PC_ = 89.7 Hz), 23.4, 21.1, 21.0; ^31^P NMR (160 MHz, CDCl_3_) *δ* 48.5, 48.4; ESI-HRMS *m*/*z* calcd. for C_40_H_56_N_2_O_8_P ([M+H]^+^) 723.3774, found 723.3783.

#### 3.1.6. General Procedure for Removal of the C-Terminal Methyl Ester Group

In phosphinotripeptides **20** and **21**, the *C*-terminal methyl ester was removed by saponification. A 4 M NaOH aqueous solution (2 equiv) was added dropwise to a solution of compound **20** or **21** dissolved in MeOH. The final concentration of the NaOH was 0.4 M. The mixture was stirred overnight at room temperature. Then, methanol was removed under reduced pressure, and the residue was diluted with water and acidified with 0.5 M HCl to pH 1 in an ice-water bath. The aqueous solution was extracted 3 times with AcOEt, and the organic layer was washed twice with water and brine. The organic layer was dried and concentrated in vacuo to give the crude product.

##### 3-[(Adamantan-1-yloxy)(1-((S)-2-(tert-butoxycarbonylamino)-4-(methylthio)butanamido)-3-phenylpropyl)phosphinyl]propanoic acid (**24**)

(403 mg, 91%) obtained as a white solid from phosphinotripeptide **20** (453 mg, 0.697 mmol), 4 M NaOH (0.35 mL, 1.394 mmol), and MeOH (3.15 mL) as described in the general procedure. mp: 171–172 °C; ^1^H NMR (400 MHz, CD_3_OD) *δ* 7.29–7.16 (m, 5H), 4.25–4.19 (m, 2H, C*H*-P, NHC*H*CO), 2.81–2.47 (m, 6H, PCH_2_C*H_2_*, CH_2_C*H_2_*S, CH_2_C*H_2_*Ph), 2.19–1.89 (m, 18H, CC*H_2_* and C*H*CH_2_ of Ad group, C*H_2_*CH_2_Ph, PC*H_2_*CH_2_, C*H_2_*CH_2_SC*H_3_*), 1.66 (s, 6H, CHC*H_2_*CH of Ad group), 1.45 (s, 9H)_major_, 1.44 (s, 9H)_minor_; ^13^C NMR (100 MHz, CD_3_OD) *δ* 175.8 (d, ^3^*J*_PC_ = 13.5 Hz), 175.1 (d, ^3^*J*_PC_ = 21.8 Hz), 158.0, 142.2, 142.1, 130.0, 129.8, 129.7, 129.6, 127.4, 127.3, 85.0 (d, ^2^*J*_PC_ = 10.0 Hz), 80.8, 55.8_minor_, 55.5_major_, 49.4 (d, ^1^*J*_PC_ = 100.8 Hz)_major_, 49.1 (d, ^1^*J*_PC_ = 107.4 Hz)_minor_, 45.6 (d, ^3^*J*_PC_ = 2.6 Hz)_minor_, 45.4 (d, ^3^*J*_PC_ = 2.9 Hz)_major_, 36.9_major_, 36.8_minor_, 33.2, 33.0 (d, ^2^*J*_PC_ = 12.5 Hz), 32.8, 31.4, 31.3, 28.9, 28.0_minor_, 27.7_major_, 23.8 (d, ^1^*J*_PC_ = 93.1 Hz)_minor_, 23.6 (d, ^1^*J*_PC_ = 93.1 Hz)_major_, 15.5_minor_, 15.4_major_; ^31^P NMR (160 MHz, CD_3_OD) *δ* 51.8_minor_, 51.6_major_; ESI-HRMS *m*/*z* calcd. for C_32_H_50_N_2_O_7_PS ([M+H]^+^) 637.3071, found 637.3071.

##### 3-[(Adamantan-1-yloxy)(1-((S)-2-(tert-butoxycarbonylamino)-4-(methylthio)butanamido)-3-methylbutyl)phosphinyl]propanoic acid (**25**)

(160 mg, 80%) obtained as a white solid from phosphinotripeptide **21** (204 mg, 0.34 mmol), 4 M NaOH (0.17 mL, 0.68 mmol), and MeOH (1.53 mL) as described in the general procedure. This compound showed to be very unstable, and it was subjected to deprotection of Boc and adamantyl groups without further purification steps. ^1^H NMR (400 MHz, CD_3_OD) *δ* 4.35–4.07 (m, 2H, C*H*-P, NHC*H*CO), 2.66–2.41 (m, 4H, PCH_2_C*H_2_*, CH_2_C*H_2_*S), 2.20–1.76 (m, 16H, CC*H_2_* and C*H*CH_2_ of Ad group, C*H_2_*CH_2_SC*H_3_*, PC*H_2_*CH_2_), 1.76–1.56 (m, 9H, CHC*H_2_*CH of Ad group, C*H*C*H_2_* of *^i^*Bu group), 1.40 (s, 9H), 0.92 (bs, 3H), 0.84 (bs, 3H); ^31^P NMR (160 MHz, CD_3_OD) *δ* 52.1, 51.9.

#### 3.1.7. General Procedure for Removal of Boc and Ad Protecting Groups

In phosphinotripeptides **20**, **21**, **23**–**29**, the Boc and Ad protecting groups were removed in acidic conditions. Then, *N*-Boc and *P*-Ad protected phosphinotripeptide derivatives were treated with a mixture of 50% TFA/CH_2_Cl_2_ and stirred for 3 h at room temperature. Then, the mixture was concentrated in vacuo. CH_2_Cl_2_ was added to the residue, and the solvent was removed under reduced pressure (3 times). A mixture of Et_2_O/hexanes 1:1 was added to the residue. The white precipitate was filtered and washed with Et_2_O/hexanes 1:1.

##### Methyl 3-[(1-((S)-2-amino-4-(methylthio)butanamido)-3-phenylpropyl)phosphinyl]propanoate (**1**)

(512 mg, 99%) obtained as a white solid from phosphinotripeptide **20** (807 mg, 1.24 mmol), and a mixture of 50% TFA/CH_2_Cl_2_ (5.6 mL) as described in the general procedure. mp: 161–164 °C; ^1^H NMR (400 MHz, DMSO-*d6*) *δ* 8.37 (bs, 2H, N*H_2_*), 7.29–7.08 (m, 5H), 3.99–3.89 (m, 2H, NHC*H*CO, C*H*-P), 3.56 (s, 3H), 2.85–2.37 (m, 6H, PCH_2_C*H_2_*, CH_2_C*H_2_*Ph, CH_2_C*H_2_*S), 2.12–1.59 (m, 6H, C*H_2_*CH_2_Ph, PC*H_2_*CH_2_, C*H_2_*CH_2_SCH_3_), 2.03 (s, 3H, SC*H_3_*); ^13^C NMR (100 MHz, DMSO-*d6*) *δ* 173.1, 172.9, 172.7, 141.5, 141.3, 128.5, 128.4, 128.3, 128.3, 125.9, 125.8, 52.0_minor_, 51.7_major_, 51.6_major_, 51.5_minor_, 47.5 (d, ^1^*J*_PC_ = 103.1 Hz)_minor_, 47.4 (d, ^1^*J*_PC_ = 103.5 Hz)_major_, 32.0_minor_, 31.8_major_, 31.3_minor_, 31.3_major_, 29.4, 28.4, 28.2, 22.7 (d, ^1^*J*_PC_ = 91.6 Hz)_major_, 22.5 (d, ^1^*J*_PC_ = 91.0 Hz)_minor_, 14.5_minor_, 14.4_major_; ^31^P NMR (160 MHz, DMSO-*d6*) *δ* 40.5, 40.3; ESI-HRMS *m*/*z* calcd. for C_18_H_30_N_2_O_5_PS ([M+H]^+^) 417.1613, found 417.1609.

##### Methyl 3-[(1-((S)-2-amino-4-(methylthio)butanamido)-3-methylbutyl)phosphinyl]propanoate (**2**)

(245 mg, 99%) obtained as a white solid from phosphinotripeptide **21** (405 mg, 0.673 mmol), and a mixture of 50% TFA/CH_2_Cl_2_ (3 mL) as described in the general procedure. mp: 183–184 °C; ^1^H NMR (400 MHz, D_2_O) *δ* 4.21–4.11 (m, 2H, C*H*-P, NHC*H*CO), 3.68 (s, 3H), 2.63–2.51 (m, 4H, PCH_2_C*H_2_*, C*H_2_*S), 2.22–2.12 (m, 2H, C*H_2_*CH_2_S), 2.09 (s, 3H), 1.95–1.88 (m, 2H, PC*H_2_*CH_2_), 1.65–1.49 (m, 3H, C*H_2_*C*H* of *^i^*Bu group), 0.90 (d, ^3^*J*_HH_ = 5.9 Hz, 3H), 0.83 (d, ^3^*J*_HH_ = 4.8 Hz, 3H); ^13^C NMR (100 MHz, D_2_O + CD_3_OD) *δ* 176.8 (d, ^3^*J*_PC_ = 15.7 Hz)_minor_, 176.4 (d, ^3^*J*_PC_ = 15.3 Hz)_major_, 169.9_major_, 169.8_minor_, 53.8, 53.6_major_, 53.5_minor_, 48.4 (d, ^1^*J*_PC_ = 102.5 Hz)_minor_, 48.2 (d, ^1^*J*_PC_ = 103.4 Hz)_major_, 36.5, 31.5_major_, 31.2_minor_, 29.3_major_, 29.3_minor_, 27.6_minor_, 27.5_major_, 23.8_minor_, 23.7_major_, 23.0 (d, ^1^*J*_PC_ = 91.1 Hz)_minor_, 22.8 (d, ^1^*J*_PC_ = 90.3 Hz)_major_, 21.2, 21.0, 15.1; ^31^P NMR (160 MHz, D_2_O) *δ* 45.6_major_, 44.3_minor_; ESI-HRMS *m*/*z* calcd. for C_14_H_30_N_2_O_5_PS ([M+H]^+^) 369.1608, found 369.1618.

##### Methyl 3-[(1-((S)-2-amino-4-(hydroxybutanamido)-3-methylbutyl)phosphinyl]propanoate (**3**)

(179 mg, 85%) obtained as a white solid from phosphinotripeptide **23** (357 mg, 0.623 mmol), and a mixture of 50% TFA/CH_2_Cl_2_ (4 mL) as described in the general procedure. mp: 174–176 °C; ^1^H NMR (400 MHz, D_2_O) *δ* 4.16–4.08 (m, 2H, C*H*-P, NHC*H*CO), 3.79–3.72 (m, 2H, C*H_2_*-OH), 3.31 (s, 3H), 2.62–2.53 (m, 2H, PCH_2_C*H_2_*), 2.21–1.80 (m, 4H, PC*H_2_*CH_2_, C*H_2_*CH_2_OH), 1.65–1.52 (m, 3H, C*H*C*H_2_* of *^i^*Bu group), 0.96–0.81 (m 6H, CH_2_CH(C*H_3_*)_2_); ^13^C NMR (100 MHz, D_2_O) *δ* 177.3 (d, ^3^*J*_PC_ = 15.9 Hz)_minor_, 177.1 (d, ^3^*J*_PC_ = 15.4 Hz)_major_, 168.9_minor_, 168.9_major_, 67.3, 48.8_major_, 48.4_minor_, 47.9, 47.3 (d, ^1^*J*_PC_ = 102.4 Hz)_minor_, 47.2 (d, ^1^*J*_PC_ = 103.3 Hz)_major_, 36.2, 35.5_minor_, 35.4_major_, 26.7_major_, 26.6_minor_, 22.7, 22.6, 22.6 (d, ^1^*J*_PC_ = 95.1 Hz)_major_, 22.0 (d, ^1^*J*_PC_ = 91.1 Hz)_minor_, 20.1; ^31^P NMR (160 MHz, D_2_O) *δ* 46.1, 45.3; ESI-HRMS *m*/*z* calcd. for C_13_H_28_N_2_O_6_P ([M+H]^+^) 339.1685, found 339.1680.

##### 3-[(1-((S)-2-Amino)-4-(methylthio)butanamido)-3-phenylpropyl)-phosphinyl]propanoic acid (**4**)

(173 mg, 99%) obtained as a white solid from phosphinotripeptide **24** (276 mg, 0.434 mmol), and a mixture of 50% TFA/CH_2_Cl_2_ (4 mL) as described in the general procedure. mp: 180–185 °C; ^1^H NMR (400 MHz, D_2_O + CD_3_OD) *δ* 7.31–7.24 (m, 5H), 3.92–3.87 (m, 1H, C*H*-P), 2.87–2.53 (m, 6H, PCH_2_C*H_2_*, CH_2_C*H_2_*S, CH_2_C*H_2_*Ph), 2.37–1.56 (m, 6H, C*H_2_*CH_2_Ph, PC*H_2_*CH_2_, C*H_2_*CH_2_SCH_3_), 2.06 (s, 3H, SC*H_3_*); ^13^C NMR (100 MHz, D_2_O + CD_3_OD) *δ* 183.8 (d, ^3^*J*_PC_ = 14.3 Hz), 183.7 (d, ^3^*J*_PC_ = 13.9 Hz), 164.9, 164.6, 143.5, 143.4, 130.5, 128.1, 128.0, 55.8, 55.7, 50.4 (d, ^1^*J*_PC_ = 100.4 Hz), 50.3 (d, ^1^*J*_PC_ = 100.0 Hz), 35.2, 35.2, 34.1 (d, ^2^*J*_PC_ = 11.1 Hz), 31.3, 31.4, 30.8, 30.6, 26.6 (d, ^1^*J*_PC_ = 91.9 Hz), 26.5 (d, ^1^*J*_PC_ = 91.6 Hz), 15.9; ^31^P NMR (160 MHz, CD_3_OD) *δ* 40.4, 40.3; ESI-HRMS *m*/*z* calcd. for C_17_H_28_N_2_O_5_PS ([M+H]^+^) 403.1457, found 403.1452.

##### 3-[(1-((S)-2-Amino-4-(methylthio)butanamido)-3-methylbutyl)phosphinyl]propanoic acid (**5**)

(95 mg, 99%) obtained as a white solid from phosphinotripeptide **25** (159 mg, 0.27 mmol), and a mixture of 50% TFA/CH_2_Cl_2_ (1 mL) as described in the general procedure. mp: 218–222 °C; ^1^H NMR (400 MHz, D_2_O) *δ* 4.25–3.96 (m, 2H, C*H*-P, NHC*H*CO), 2.73–2.40 (m, 4H, PCH_2_C*H_2_*, C*H_2_*S), 2.28–1.99 (m, 5H, C*H_2_*CH_2_SC*H_3_*), 1.95–1.75 (m, 2H, PC*H_2_*CH_2_), 1.70–1.38 (m, 3H, C*H_2_*C*H* of *^i^*Bu group), 0.90 (bs, 3H), 0.82 (bs, 3H); ^13^C NMR (100 MHz, D_2_O + CD_3_OD) *δ* 178.4 (d, ^3^*J*_PC_ = 14.8 Hz)_minor_, 178.0 (d, ^3^*J*_PC_ = 16.3 Hz)_major_, 169.9, 53.8_minor_, 53.5_major_, 48.2 (d, ^1^*J*_PC_ = 101.6 Hz)_minor_, 48.0 (d, ^1^*J*_PC_ = 103.8 Hz)_major_, 36.6_minor_, 36.5_major_, 31.4_major_, 31.2_minor_, 29.3, 27.9_minor_, 27.8_major_, 23.9_minor_, 23.8_major_, 23.3 (d, ^1^*J*_PC_ = 86.8 Hz)_minor_, 23.2 (d, ^1^*J*_PC_ = 92.1 Hz)_major_, 21.3, 21.0, 15.1; ^31^P NMR (160 MHz, D_2_O) *δ* 43.9_major_, 42.8_minor_; ESI-HRMS *m*/*z* calcd. for C_13_H_28_N_2_O_5_PS ([M+H]^+^) 355.1457, found 355.1461.

##### Methyl 3-[(1-((S)-3-([1,1′-biphenyl]-4-yl)-2-aminopropanamido)-3-phenylpropyl)phosphinyl]-propanoate (**6**)

(345 mg, 99%) obtained as a white solid from phosphinotripeptide **26** (509 mg, 0.686 mmol), and a mixture of 50% TFA/CH_2_Cl_2_ (5 mL) as described in the general procedure. mp: 126–128 °C; ^1^H NMR (400 MHz, CD_3_OD) δ 7.64–6.94 (m, 14H), 4.28–4.02 (m, 2H, CH-P, NH_2_CHCO), 3.65 (s, 3H), 3.60 (s, 3H), 3.43–3.06 (m, 2H, CH_2_-biphenyl), 2.86–2.47 (m, 4H, PCH_2_CH_2_, CH_2_CH_2_Ph), 2.25–1.65 (m, 4H, PCH_2_CH_2_, CH_2_CH_2_Ph); ^13^C NMR (100 MHz, CD_3_OD) δ 175.6 (d, ^3^*J*_PC_ = 16.1 Hz), 175.1 (d, ^3^*J*_PC_ = 17.1 Hz), 169.8, 169.3, 142.8, 142.5, 142.2, 141.9, 141.7, 134.9, 131.2, 131.2, 130.1, 129.9, 129.8, 129.7, 129.6, 129.5, 129.0, 128.8, 128.7, 128.5, 128.1, 128.0, 127.2, 127.0, 56.3, 55.8, 52.5, 52.5, 38.5, 38.2, 33.9, 33.8, 31.6, 30.9, 27.9, 27.8, 24.2 24.2 (d, ^1^*J*_PC_ = 91.6 Hz), 24.0 (d, ^1^*J*_PC_ = 91.4 Hz); ^31^P NMR (160 MHz, CD_3_OD) δ 42.9, 40.7; ESI-HRMS m/z calcd. for C_28_H_34_N_2_O_5_P ([M+H]^+^) 509.2205, found 509.2210.

##### Methyl 3-[(1-((S)-3-([1,1′-biphenyl]-4-yl)-2-aminopropanamido)-3-methylbutyl)phosphinyl]-propanoate (**7**)

(209 mg, 78%) obtained as a white solid from phosphinotripeptide **27** (405 mg, 0.583 mmol), and a mixture of 50% TFA/CH_2_Cl_2_ (4 mL) as described in the general procedure. mp: 150–152 °C; ^1^H NMR (400 MHz, CD_3_OD) *δ* 7.81–7.32 (m, 9H), 4.64–4.13 (m, 2H, C*H*-P, NH_2_C*H*CO), 3.67 (s, 3H), 3.61 (s, 3H), 3.53–3.05 (m, 2H, C*H_2_*-biphenyl), 2.75–2.57 (m, 2H, PCH_2_C*H_2_*), 1.93–1.22 (m, 5H, PC*H_2_*CH_2_, C*H_2_*C*H* of *^i^*Bu group), 0.98 (d, ^3^*J*_HH_ = 5.9 Hz, 3H), 0.92 (d, ^3^*J*_HH_ = 5.9 Hz, 3H), 0.71 (d, ^3^*J*_HH_ = 6.4 Hz, 3H), 0.68 (d, ^3^*J*_HH_ = 6.3 Hz, 3H); ^13^C NMR (100 MHz, CD_3_OD) *δ* 175.6 (d, ^3^*J*_PC_ = 16.3 Hz), 175.1 (d, ^3^*J*_PC_ = 17.1 Hz), 169.6, 169.1, 142.2, 141.9, 141.8, 134.9, 134.8, 131.2, 130.1, 128.9, 128.7, 128.6, 128.1, 128.0, 56.2, 55.7, 52.6, 52.5, 38.5, 38.1, 37.8, 37.3, 27.9, 27.8, 25.8 (d, ^3^*J*_PC_ = 10.8 Hz), 25.3 (d, ^3^*J*_PC_ = 10.7 Hz), 24.1, 23.3, 23.1, 21.6, 21.4; ^31^P NMR (160 MHz, CD_3_OD) *δ* 43.8, 41.6; ESI-HRMS *m*/*z* calcd. for C_24_H_34_N_2_O_5_P ([M+H]^+^) 461.2205, found 461.2215.

##### Methyl 3-[(1-((S)-2-amino-3-(4-benzoylphenyl)propanamido)-3-phenylpropyl)phosphinyl]-propanoate (**8**)

(377 mg, 99%) obtained as a white solid from phosphinotripeptide **28** (547 mg, 0.710 mmol), and a mixture of 50% TFA/CH_2_Cl_2_ (5 mL) as described in the general procedure. mp: 134–140 °C (dec); ^1^H NMR (400 MHz, CD_3_OD) δ 7.79–6.92 (m, 14H), 4.33–4.02 (m, 2H, CH-P, NH_2_CHCO), 3.65 (s, 3H), 3.61 (s, 3H), 3.59–3.15 (m, 2H, CH_2_-4-benzoylphenyl), 2.83–2.53 (m, 4H, PCH_2_CH_2_, CH_2_CH_2_Ph), 2.20–1.73 (m, 4H, PCH_2_CH_2_, CH_2_CH_2_Ph); ^13^C NMR (100 MHz, CD_3_OD) δ 198.2, 197.9, 175.6 (d, ^3^*J*_PC_ = 16.8 Hz), 175.1 (d, ^3^*J*_PC_ = 14.7 Hz), 169.6, 169.1, 142.7, 142.4, 141.3, 141.0, 138.8, 138.7, 138.3, 138.0, 133.8, 132.8, 132.1, 132.0, 131.2, 131.0, 130.9, 129.7, 129.6, 129.5, 127.2, 127.1, 55.8, 55.5, 52.6, 52.6, 38.6, 38.3, 33.7, 33.6, 31.5, 31.0, 27.8, 27.7, 24.1 (d, ^1^*J*_PC_ = 103.4 Hz), 24.0 (d, ^1^*J*_PC_ = 96.0 Hz); ^31^P NMR (160 MHz, CD_3_OD) δ 40.7, 38.9; ESI-HRMS m/z calcd. for C_29_H_34_N_2_O_6_P ([M+H]^+^) 537.2154, found 537.2166.

##### Methyl 3-[(1-((S)-2-amino-3-(4-benzoylphenyl)propanamido)-3-methylbutyl)phosphinyl]-propanoate (**9**)

(334 mg, 99%) obtained as a white solid from phosphinotripeptide **29** (500 mg, 0.692 mmol), and a mixture of 50% TFA/CH_2_Cl_2_ (5 mL) as described in the general procedure. mp: 124–126 °C; ^1^H NMR (400 MHz, CD_3_OD) δ 7.81–7.45 (m, 9H), 4.26–4.13 (m, 2H, CH-P, NH_2_CHCO), 3.68 (s, 3H), 3.65 (s, 3H), 3.46–3.12 (m, 2H, CH_2_-4-benzoylphenyl), 2.63–2.57 (m, 2H, PCH_2_CH_2_), 1.95–1.29 (m, 5H, PCH_2_CH_2_, CH_2_CH of ^i^Bu group), 1.00 (d, ^3^*J*_HH_ = 6.4 Hz, 3H), 0.93 (d, ^3^*J*_HH_ = 6.3 Hz, 3H), 0.77 (d, ^3^*J*_HH_ = 6.6 Hz, 3H), 0.74 (d, ^3^*J*_HH_ = 6.6 Hz, 3H); ^13^C NMR (100 MHz, CD_3_OD) δ 198.2, 197.9, 175.7 (d, ^3^*J*_PC_ = 16.2 Hz), 175.1 (d, ^3^*J*_PC_ = 16.5 Hz), 169.3, 168.8, 141.1, 140.9, 138.8, 138.8, 138.5, 138.2, 134.1, 134.1, 132.0, 131.9, 131.2, 131.0, 130.8, 130.8, 129.7, 55.9, 55.4, 52.6, 52.5, 38.7, 38.3, 37.3, 37.0, 27.8, 27.7, 25.8 (d, ^3^*J*_PC_ = 11.0 Hz), 25.5 (d, ^3^*J*_PC_ = 11.1 Hz), 24.1, 24.0 (d, ^1^*J*_PC_ = 91.1 Hz), 23.8 (d, ^1^*J*_PC_ = 92.1 Hz), 21.5, 21.3, ^31^P NMR (160 MHz, CD_3_OD) δ 45.3, 43.1; ESI-HRMS *m*/*z* calcd. for C_25_H_34_N_2_O_6_P ([M+H]^+^) 489.2154, found 489.2170.

### 3.2. Inhibitor Activity

#### 3.2.1. Enzyme Preparation

Leucyl aminopeptidase (SsLAP) was isolated from the porcine kidney modified method described by Spackman [56] and Ledeme [57]. K_M_ of the isolated enzyme on the synthetic substrate _L_-leucine-p-nitroanilide (_L_-Leu-pNA) was 0.86 mM, and V_max_ was 0.0012 μmol/min. K_M_ values correlate well with those given in the works 0.77 mM [58,59]. The purity of the enzyme was confirmed by electrophoresis.

The aminopeptidase from barley seeds (HvLAP) was isolated and purified according to the procedure described by Oszywa [46]. K_M_ of the isolated enzyme on the synthetic substrate _L_-leucine-p-nitroanilide (_L_-Leu-pNA) was 0.55 mM, and V_max_ was 0.0146 μmol/min [46,48]. The purity of the enzyme was confirmed by electrophoresis.

#### 3.2.2. Kinetic Assays

The enzymatic reaction in the presence of SsLAP was carried out at 37 °C in 7.5 mM TEA-HCl buffer (pH 8.5), containing 5 mM NaCl. The progress of the reaction was monitored spectrophotometrically (UV-VIS Spectrophotometer Cintra 303, GBC Scientific Equipment Ltd., Victoria, Australia) at a wavelength of 405 nm against a control sample containing no enzyme. The attempting mixture contained: Solution of the synthetic substrate _L_-Leu-p-nitroanilide in DMSO (substrate concentration: 1.5 to 0.2 mM—final concentration), 7.5 mM TEA-HCl buffer (pH 8.5), containing 5 mM NaCl, the solution of the potential inhibitor in reaction buffer (concentration of compound depending on the braking force), and enzyme (0.005 mg of the sample).

The enzymatic reaction in the presence of aminopeptidase from barley seeds (HvLAP) was carried out at 37 °C in 50 mM Tris-HCl buffer (pH 7.5), containing 50 mM NaCl, and 10 mM 2-mercaptoethanol. The progress of the reaction was monitored spectrophotometrically (UV-VIS Spectrophotometer Cintra 303, GBC Scientific Equipment Ltd., Victoria, Australia) at a wavelength of 405 nm against a control sample containing no enzyme. The attempting mixture contained: Solution of the synthetic substrate _L_-Leu-p-nitroanilide in DMSO (substrate concentration: 1.5 to 0.2 mM—final concentration), 50 mM Tris-HCl buffer (pH 7.5), containing 50 mM NaCl, and 10 mM 2-mercaptoethanol, the solution of the potential inhibitor in reaction buffer (concentration of compound depending on the braking force), and enzyme (0.028 mg of the sample).

#### 3.2.3. Kinetic Calculation

Kinetic constants K_M_, V_max_, and K_I_ and type of inhibition was determined by methods Lineweaver-Burk, Dixon, Hanes-Woolf, and method half-inhibitory concentration. In the calculation, linearization methods used weighted regression. The values (Ki) given in the tables are the values calculated by the Dixon method. The Ki values calculated with all the above-mentioned methods close to each other. All measurements were made in three-fold repetitions. The graphs show the average values of the three measurements and S_b_.

The used weight factors have been calculated as follow:

w_i_ = 1/(σ_i_^2^), σ^2^ = (dy/dx)^2^, where y = 1/V, or σ^2^ = (dy/dx)^2^ which gives the formula for weight I = V^4^; w_i_—statistical weight of the i-th measurement, σ_i_^2^—variance of the i-th experimental point.

### 3.3. Molecular Modeling

Due to synthetic route designed inhibitors contain S isomers of amino acids in the P2 position, whereas in the P1 position, both R and S stereoisomers are possible. Because of that, all combinations of R and S stereoisomers in the P1 position with stereoisomer S in the P2 position for all phosphinic inhibitors were considered during molecular modeling studies. The structures of all the ligands were optimized in program Gaussian09 at the B3LYP/6-311g (d,p) level of theory [60] with the PCM solvent model using water as the solvent. This protocol was applied for the designed phosphinic inhibitors, as well as for the phosphonic analog of leucine, a well-known inhibitor of LAP, which was considered as the reference compound, serving to verify the docking methodology.

Next, ligands were prepared with the Discover Studio Visualizer 5 (Dassault Systems BIOVIA, Waltham, MA, USA). For each ligand, 10 of the most possible conformers have been generated and use in docking protocol. For further analysis, five of them with the best value of GoldScore function have been selected. As a model of mammalian leucine aminopeptidase, the crystal structures from bovine lens LAP, whereas as the plant aminopeptidase model, the structure of tomato aminopeptidase has been taken. The structures were obtained from the RCSB Protein Data Bank ID: 1LCP [51] and 4KSI [52]. Lacking protons and charges were added to the protein using the H++ server [54] according to the experimental pH value. The ligand, besides metal ions, was removed from the structure of the enzymes. The active site sphere was selected based on the location of zinc Zn408 and magnesium Mg601 ions for SsLAP and HvLAP*,* respectively, with the radius 15Ang. During the docking protocol, the protein molecules have been rigid. Molecular docking in the defined active sites was carried out using GOLD Algorithm (2010, 1 version, CCDC, Cambridge, UK) [53] with applied Genetic Algorithm protocol. Analysis of inhibitor-enzyme interactions of docked molecules was performed with Discovery Studio Visualizer 5 (Dassault Systems BIOVIA, Waltham, MA, USA).

## 4. Conclusions

It is known that phosphinic moiety is a good analog of the transition state during peptide bond hydrolysis. It is possible thanks to imitating the tetrahedral geometry, electron distribution, and the ability to metal complexation. Application of the phosphonic moiety to construct pseudopeptides, which are effective inhibitors of therapeutically significant metalloproteases, including porcine and barley leucine aminopeptidase, obtain one of the most potent inhibitors of these enzymes. Because both enzymes are responsible for the consecutive removal of the N-terminal residue from the peptidic substrates, elongation of the N-terminal parts of the inhibitor is not suggested. However, within this work, we showed that elongation of the phosphinatedipeptides by adding one more amino acid residue in the P2 position will not deprive their biological properties. Obtained compounds are micromolar inhibitors of SsLAP and HvLAP. The best inhibitor activity has been found for **3** toward SsLAP (K_i_ = 14 μM) and **1** toward HvLAP (K_i_ = 38 μM). Moreover, the introduction of additional amino acid residue in the P2 position induced the higher selectivity of the final tripeptides.

Additionally, based on the retrosynthesis analysis, we proposed a synthetic route to obtain phosphinate pseudotripeptide **1**–**9** within a reasonable number of steps with a satisfactory overall yield.

## Figures and Tables

**Table 1:** The activity of pseudotripeptides as inhibitors of porcine LAP and barley LAP.

Entry		Structure	K_I_ [μM]	
			SsLAP	HvLAP
1	Met-hPheP[CH_2_]Gly-OMe	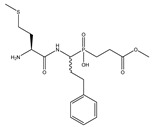	532 ± 46	38 ± 9
2	Met-LeuP[CH_2_]Gly-OMe	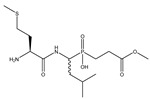	1090 ± 73	649 ± 43
3	Ser-LeuP[CH_2_]Gly-OMe	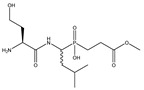	14 ± 1	121 ± 19
4	Met-hPheP[CH_2_]Gly	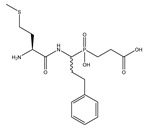	229 ± 40	228 ± 10
5	Met-LeuP[CH_2_]Gly	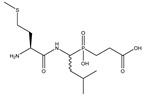	3618 ± 433	1035 ± 102
6	Bip-hPheP[CH_2_]Gly-OMe	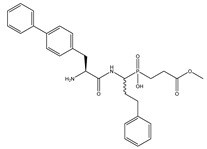	175 ± 3	878 ± 70
7	Bip-LeuP[CH_2_]Gly-OMe	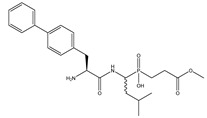	130 ± 14	1303 ± 84
8	Bpa-hPheP[CH_2_]Gly-OMe	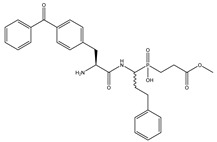	95 ± 9	159 ± 25
9	Bpa-LeuP[CH_2_]Gly-OMe	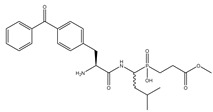	507 ± 31	797 ± 76

## Supplementary Materials

The following are available online at https://www.mdpi.com/article/10.3390/ijms22105090/s1.
