# Supplementary material for: Phosphinotripeptidic Inhibitors of Leucylaminopeptidases
Source: Int J Mol Sci. 2021 May 11;22(10):5090. doi: 10.3390/ijms22105090 (PMC8151835; doi:10.3390/ijms22105090)
Supplement: Supplementary file 1 [file ijms-22-05090-s001.zip › ijms-1200928-supplementary.pdf]

Electronic Supplementary Data for online publication

Phosphinotripeptidic inhibitors of leucylaminopeptidases.

Michał Jewgiński [1]*, Kinga Haremza [1], Jesús M. de los Santos [2], Zouhair Es Sbai [2], Bartosz Oszywa [3], Małgożata Pawełczak [3], Francisco Palacios [2], Rafał Latajka [1]

[1] Department of Bioorganic Chemistry, Wrocław University of Science and Technology, Wybrzeże Wyspiańskiego 27, 50-370 Wrocław, Poland
[2] Departamento de Química Orgánica I, Facultad de Farmacia, Centro de Investigaciones y Estudios Avanzados "Lucio Lascaray", Universidad del País Vasco, Paseo de la Universidad, 7. 01006 Vitoria, Spain.
[3] Faculty of Chemistry, University of Opole, Oleska 48, 45-052 Opole, Poland
* Correspondence: michal.jewginski@pwr.edu.pl; Tel.: +48 71 320 24 61

Table of Contents

1	**Figure S1**. Proton – 1H (top) and carbon – 13C (bottom) NMR spectra for compound **12**	S3
2	**Figure S2**. Proton – 1H (top) and carbon – 13C (bottom) NMR spectra for compound **13**	S4
3	**Figure S3**. Proton – 1H (top) and carbon – 13C (bottom) NMR spectra for compound **14**	S5
4	**Figure S4**. Proton – 1H (top) and carbon – 13C (bottom) NMR spectra for compound **15**	S6
5	**Figure S5**. Proton – 1H (top) and carbon – 13C (bottom) NMR spectra for compound **16**	S7
6	**Figure S6**. Proton – 1H (top) and carbon – 13C (bottom) NMR spectra for compound **17**	S8
7	**Figure S7**. Proton – 1H (top) and carbon – 13C (bottom) NMR spectra for compound **20**	S9
8	**Figure S8**. Proton – 1H (top) and carbon – 13C (bottom) NMR spectra for compound **21**	S10
9	**Figure S9**. Proton – 1H (top) and carbon – 13C (bottom) NMR spectra for compound **22**	S11
10	**Figure S10**. Proton – 1H NMR spectra for compound **23**	S12
11	**Figure S11**. Proton – 1H (top) and carbon – 13C (bottom) NMR spectra for compound **26**	S13
12	**Figure S12**. Proton – 1H (top) and carbon – 13C (bottom) NMR spectra for compound **27**	S14
13	**Figure S13**. Proton – 1H (top) and carbon – 13C (bottom) NMR spectra for compound **28**	S15
14	**Figure S14**. Proton – 1H (top) and carbon – 13C (bottom) NMR spectra for compound **29**	S16
15	**Figure S15**. Proton – 1H (top) and carbon – 13C (bottom) NMR spectra for compound **24**	S17
16	**Figure S16**. Proton – 1H NMR spectra for compound **25**	S18
17	**Figure S17**. Proton – 1H NMR spectra for compound 1	S19
18	**Figure S18**. Proton – 1H NMR spectra for compound **2**	S20
19	**Figure S19**. Proton – 1H NMR spectra for compound **3**	S21
20	**Figure S20**. Proton – 1H NMR spectra for compound **4**	S22
21	**Figure S21**. Proton – 1H NMR spectra for compound **5**	S23
22	**Figure S22**. Proton – 1H NMR spectra for compound **6**	S24
23	**Figure S23**. Proton – 1H NMR spectra for compound **7**	S25
24	**Figure S24**. Proton – 1H NMR spectra for compound **8**	S26
25	**Figure S25**. Proton – 1H NMR spectra for compound **9**	S27
26	**Table S1.** Dixon plots for investigated compounds in the presence of *SsLAP*	S28
27	**Table S2.** Dixon plots for investigated compounds in the presence of *HvLAP*	S30
28	**Figure S26**. Comparison of alignments of $LeuPO_3H_2$ in bovine lens LAP in crystal structure (magenta) (PDB ID: *1LCP*) and docked with GOLD software (cyan)	S32
29	**Table S3.** GoldScore Fitness values for the investigated compounds 1-9 and mammalian and plant aminopeptidases.	S33
30	**Figure S27**. Modes of binding of all the studied phosphinic pseudotripeptides by bovine lens leucyl aminopeptidase [PDB ID: 1LCP] with showed specific intermolecular interactions: a),b) **1**-*SR* isomer; c),d)	S34

	-**1**-SS isomer; e),f) **2**-*SR* isomer; g),h) **2**-SS *isomer;* i),j) **3**-*SR* isomer*;* k),l) **3** – *SS* isomer; m),n) **4** -*SR* isomer; o),p) **4** – *SS* isomer; q),r) **5** -*SR* isomer; s),t) **5** -*SS* isomer; u),v) **6** – *SR* isomer; w),x) **6** – *SS* isomer.	
31	**Figure S28**. Modes of binding of all the studied phosphinic pseudotripeptides by bovine lens leucyl aminopeptidase [PDB ID: 1LCP] with showed specific intermolecular interactions: a),b) **7**-*SR* isomer; c),d) **7**-SS isomer; e),f) **8**-*SR* isomer; g),h) **8**-SS *isomer;* i),j) **9**-*SR* isomer*;* k),l) **9** – *SS* isomer.	S35
32	**Figure S29**. Modes of binding of all the studied phosphinic pseudotripeptides by tomato amino peptidase [PDB ID: 4KSI] with showed specific intermolecular interactions: a),b) **1**-*SR* isomer; c),d) -**1**-SS isomer; e),f) **2**-*SR* isomer; g),h) **2**-SS *isomer;* i),j) **3**-*SR* isomer*;* k),l) **3** – *SS* isomer; m),n) **4** -*SR* isomer; o),p) **4** – *SS* isomer; q),r) **5** -*SR* isomer; s),t) **5** -*SS* isomer; u),v) **6** – *SR* isomer; w),x) **6** – *SS* isomer.	S36
33	**Figure S30**. Modes of binding of all the studied phosphinic pseudotripeptides by tomato aminopeptidase [PDB ID: 4KSI] with showed specific intermolecular interactions: a),b) **7**-*SR* isomer; c),d) **7**-SS isomer; e),f) **8**-*SR* isomer; g),h) **8**-*SS* isomer*;* i),j) **9**-*SR* isomer*;* k),l) **9**-*SS* isomer.	S37

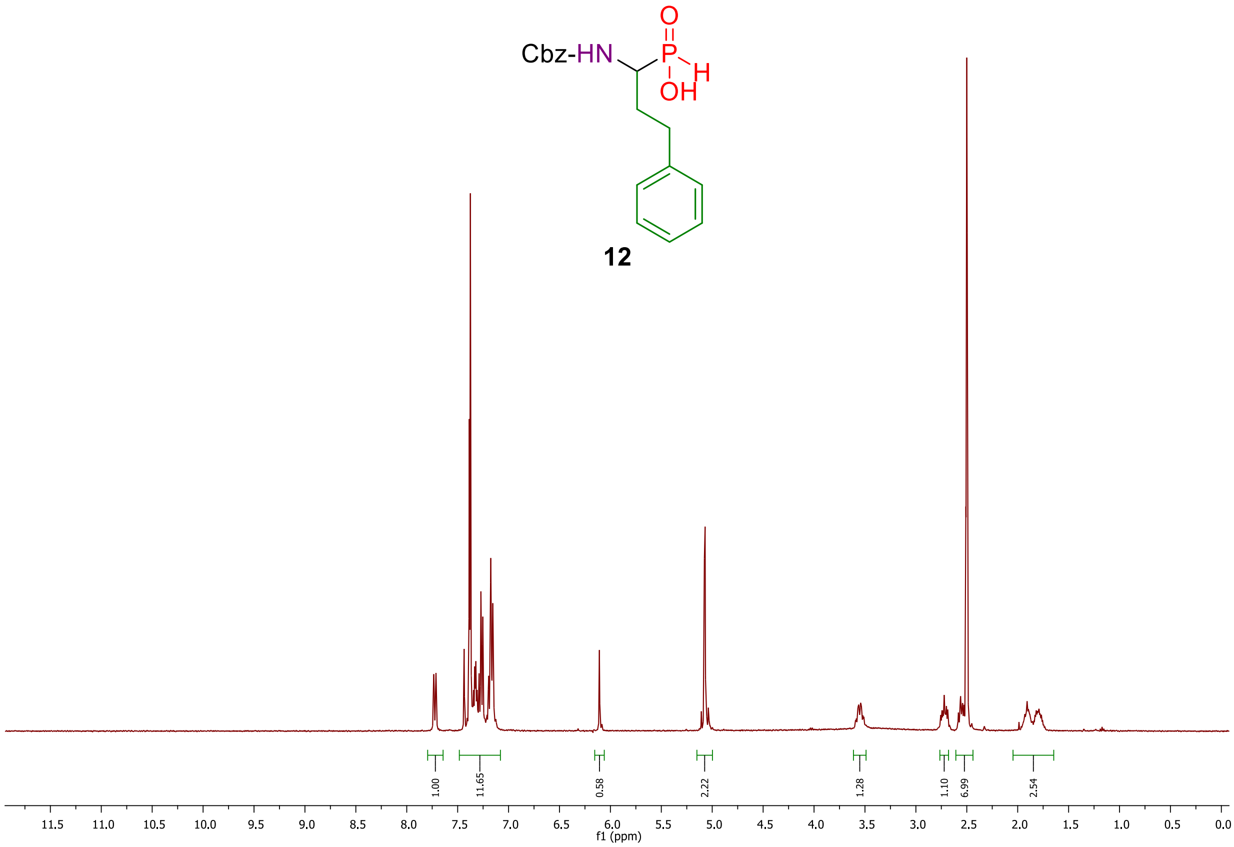

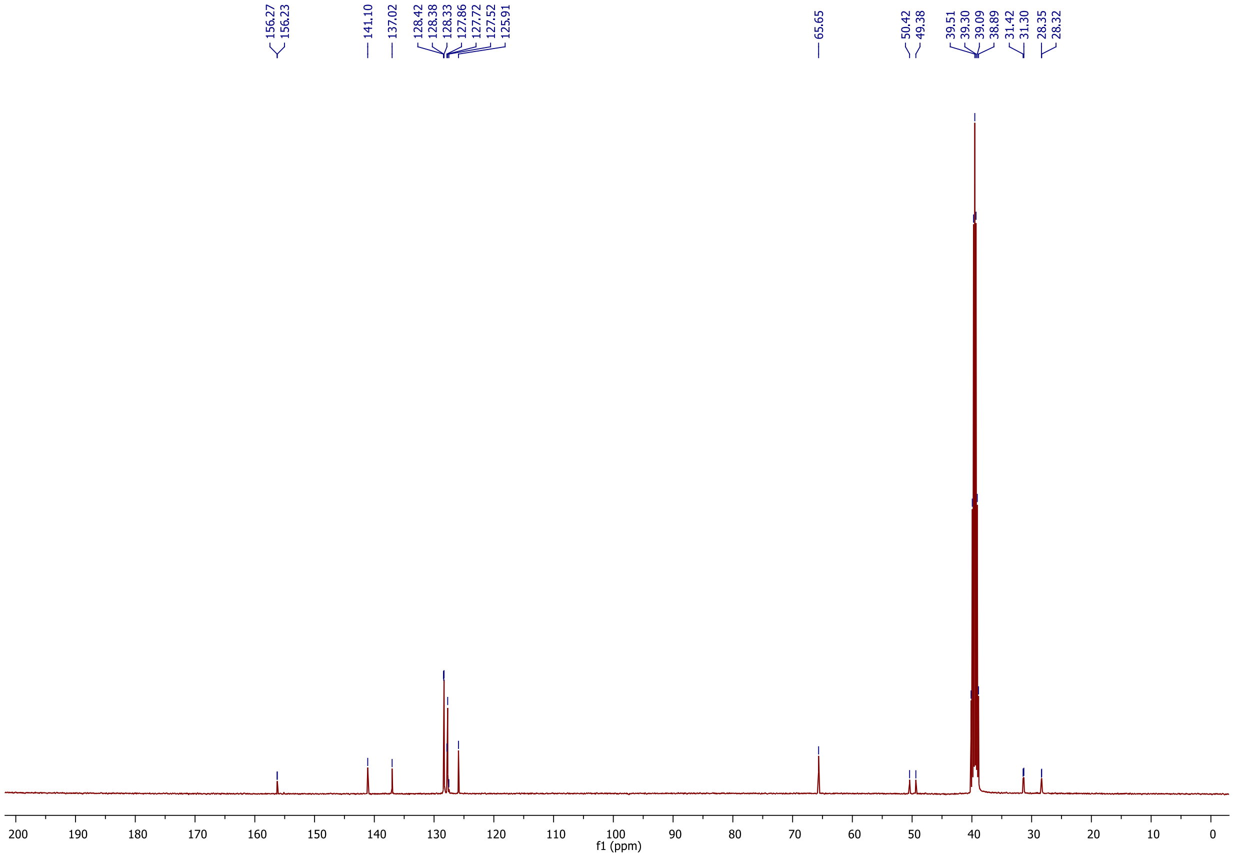

Figure S2. Proton – 1H (top) and carbon – 13C (bottom) NMR spectra for compound **12**

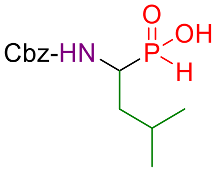

13

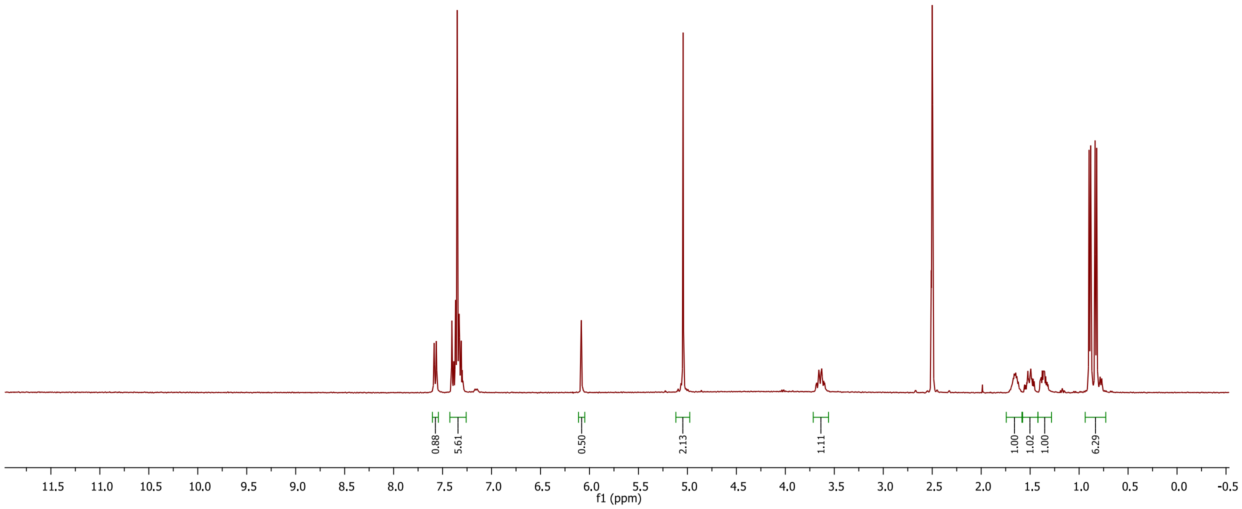

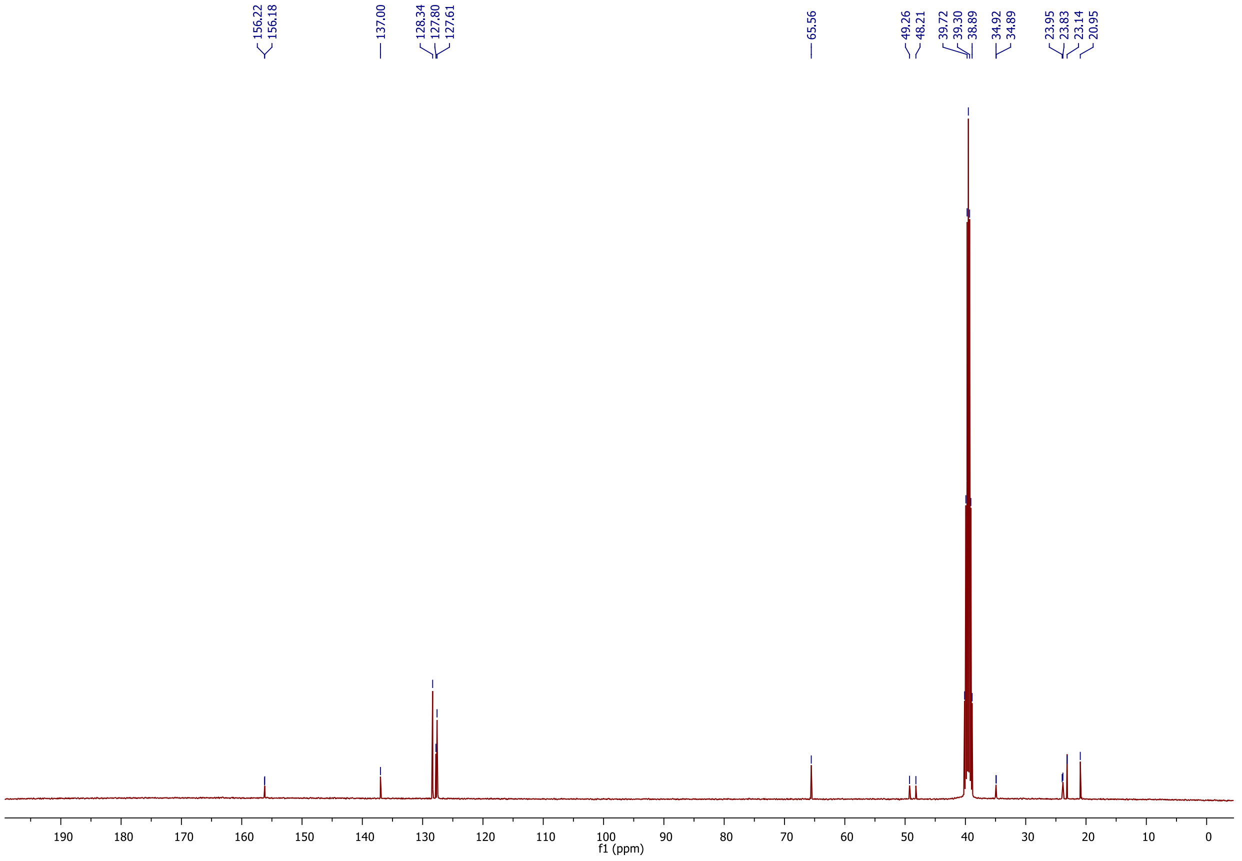

Figure S2. Proton – 1H (top) and carbon – 13C (bottom) NMR spectra for compound **13**

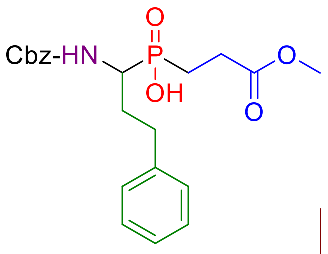

14

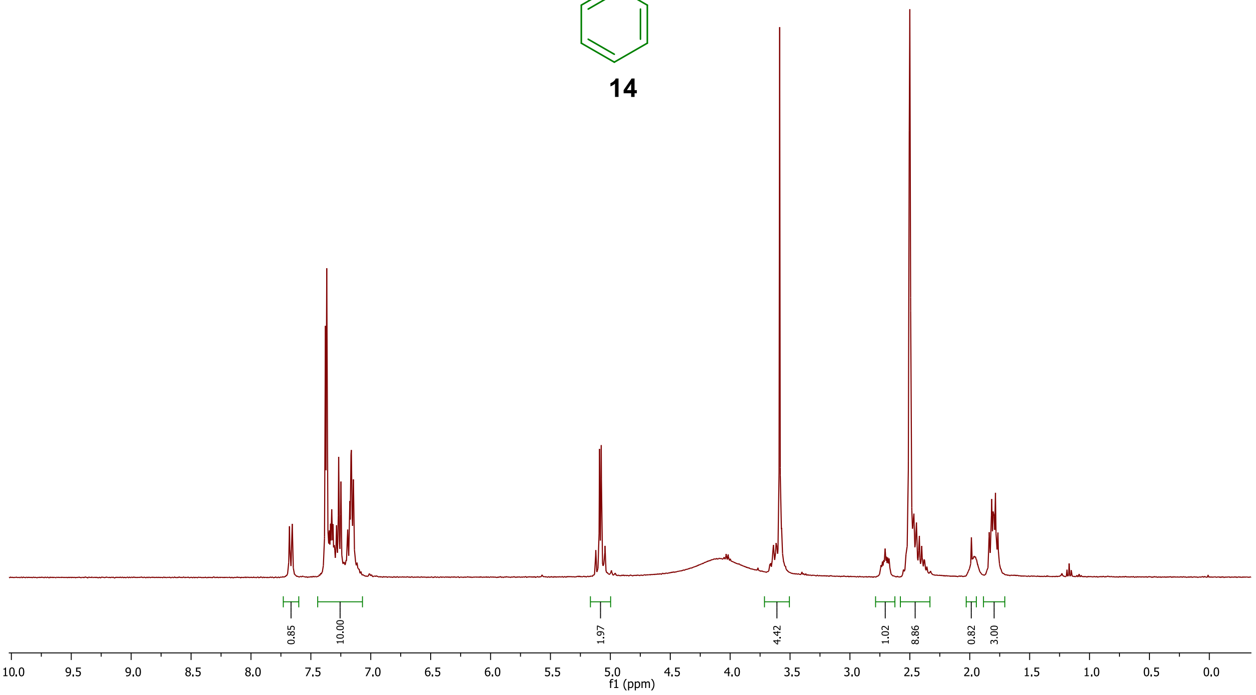

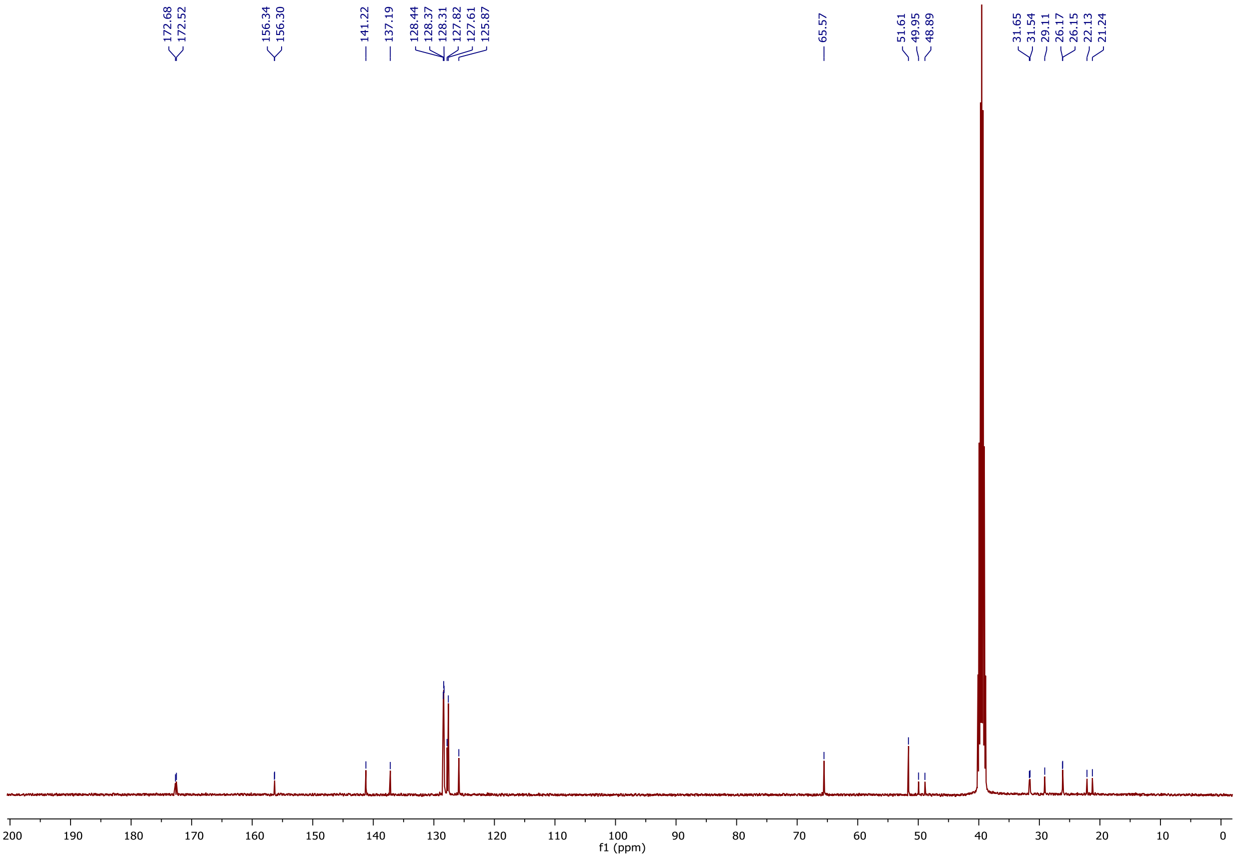

Figure S3. Proton – 1H (top) and carbon – 13C (bottom) NMR spectra for compound **14**

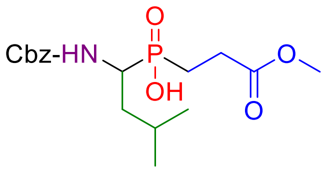

15

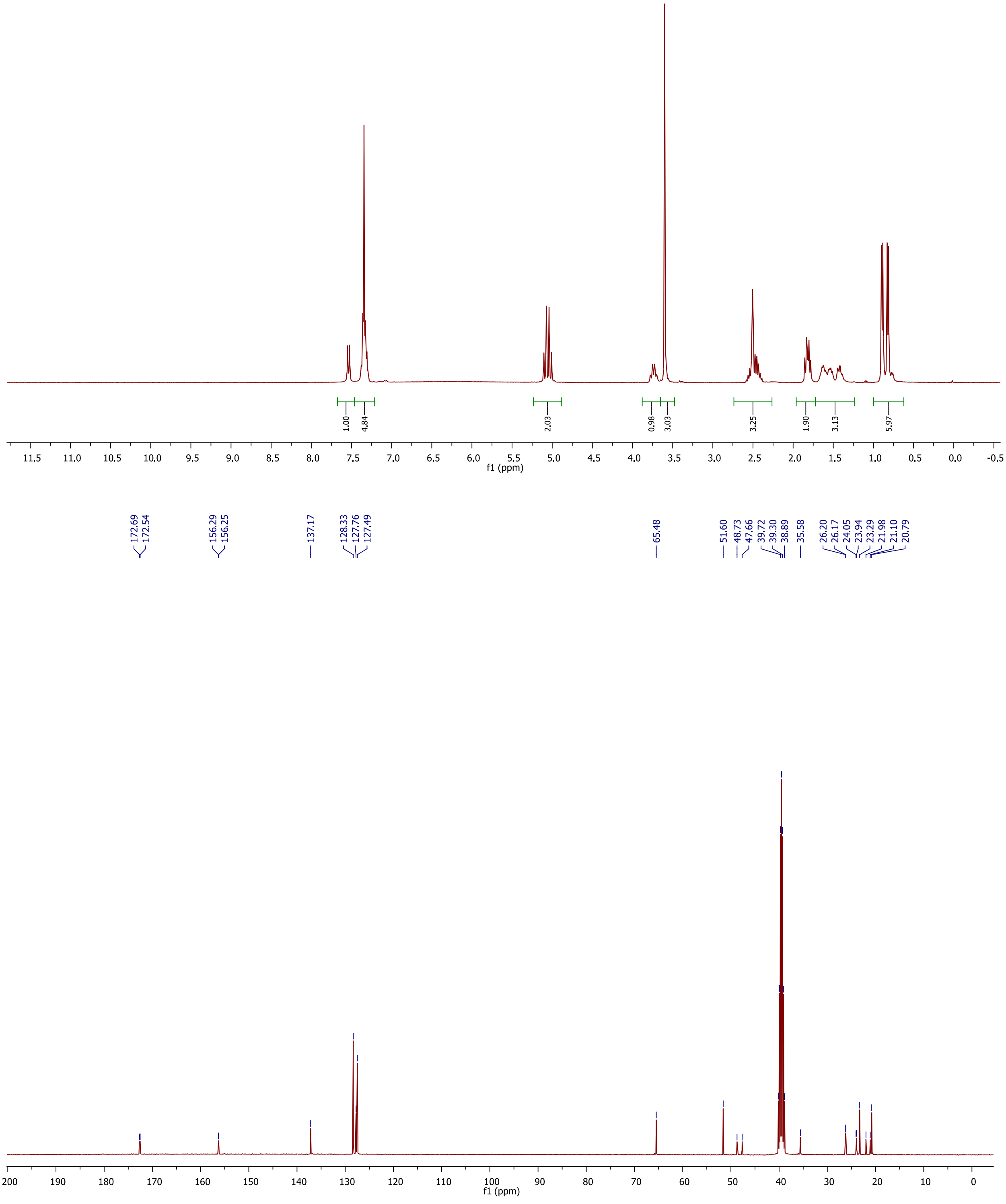

Figure S4. Proton – 1H (top) and carbon – 13C (bottom) NMR spectra for compound **15**

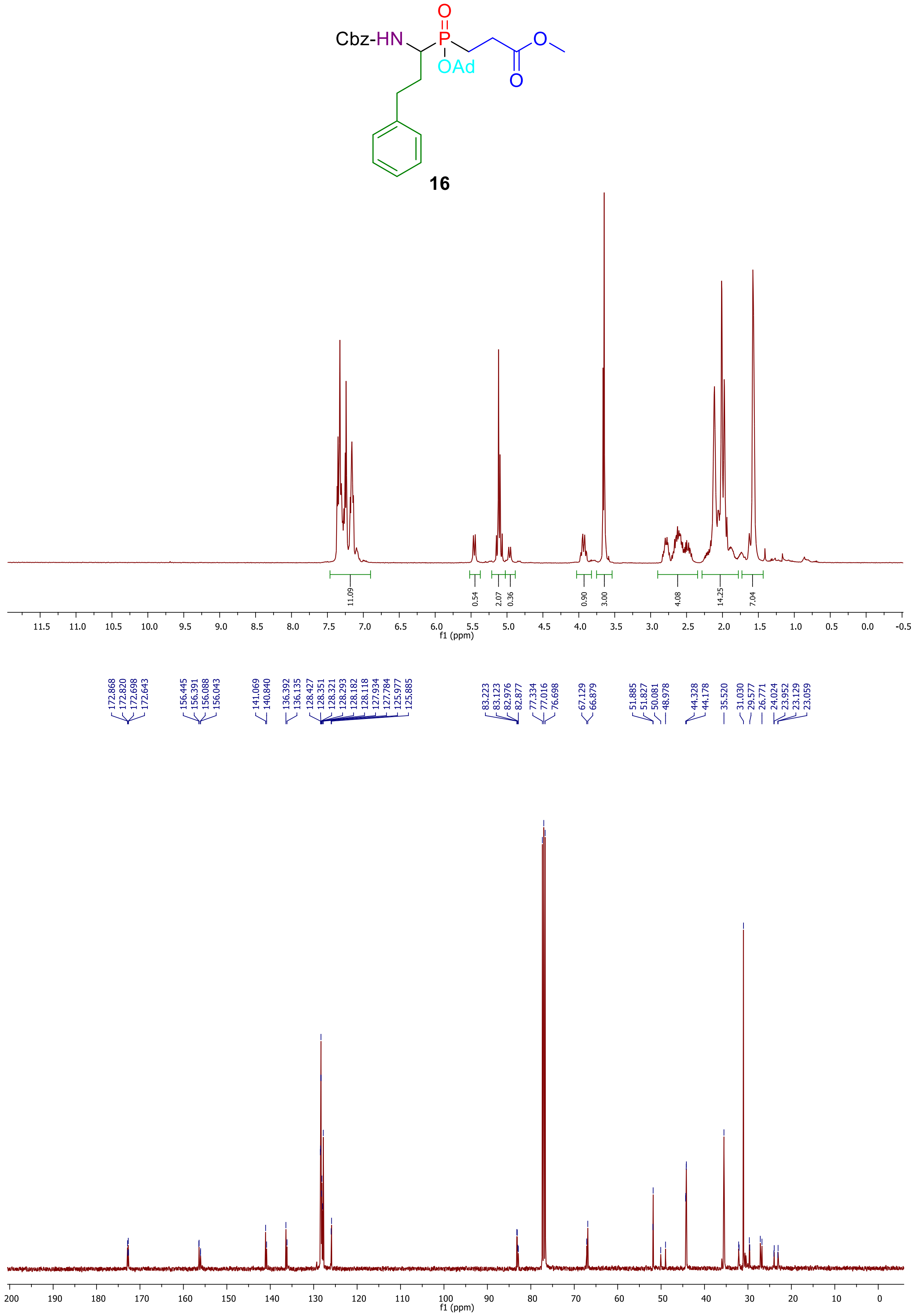

Figure S5. Proton – 1H (top) and carbon – 13C (bottom) NMR spectra for compound **16**

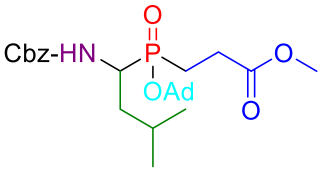

17

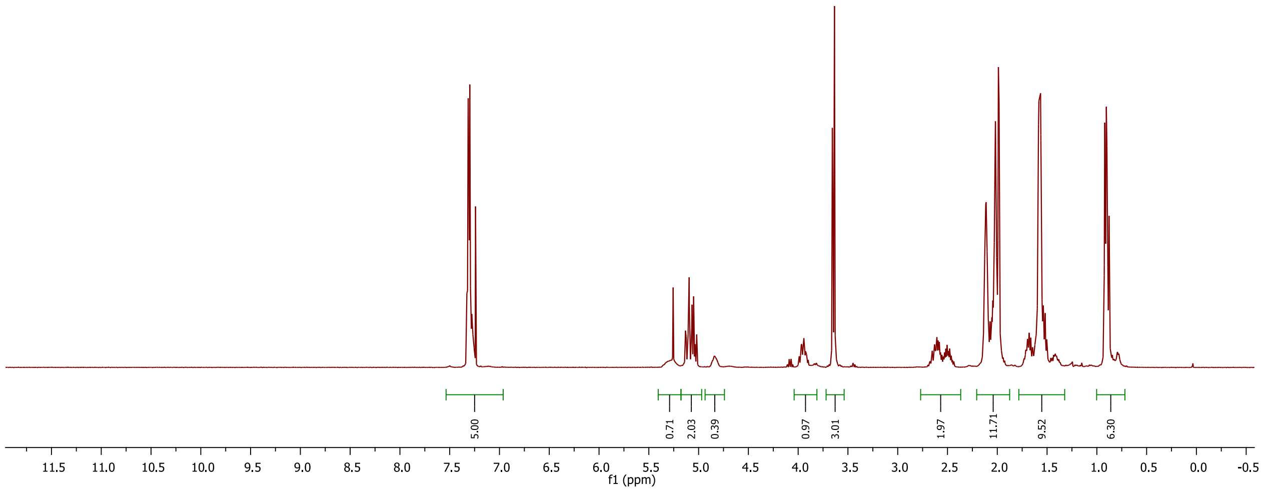

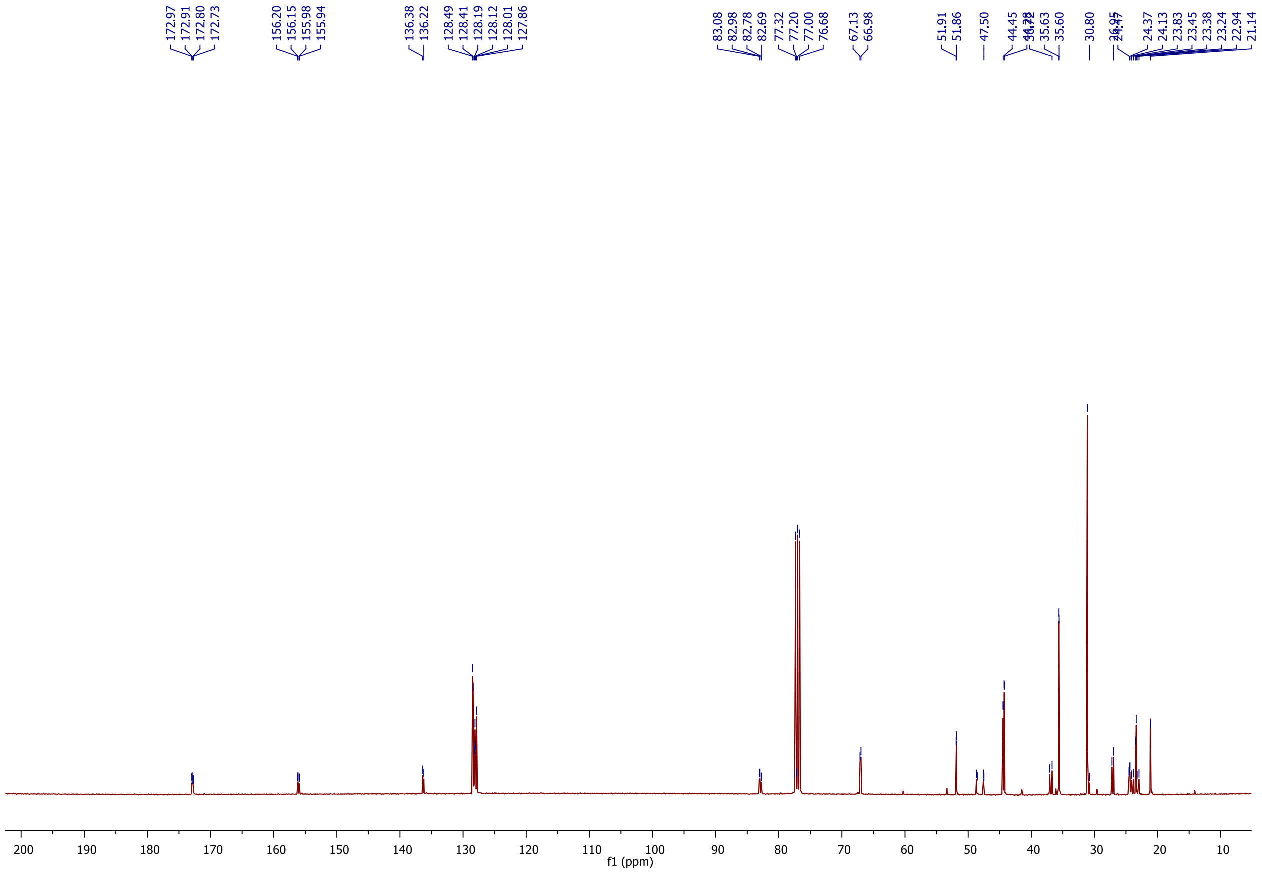

Figure S6. Proton – 1H (top) and carbon – 13C (bottom) NMR spectra for compound **17**

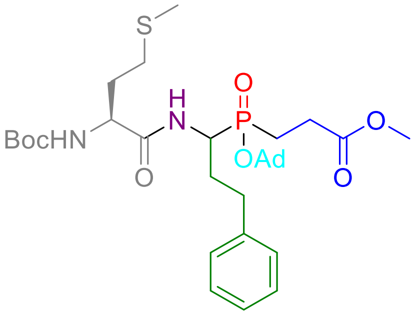

20

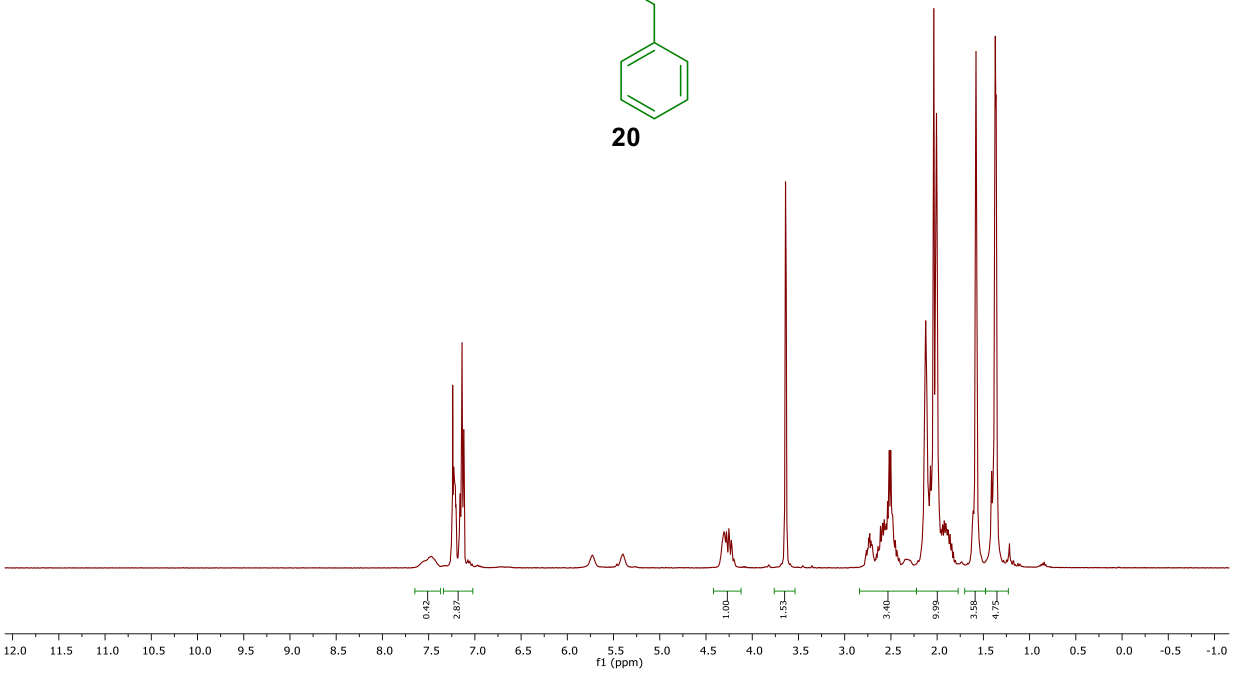

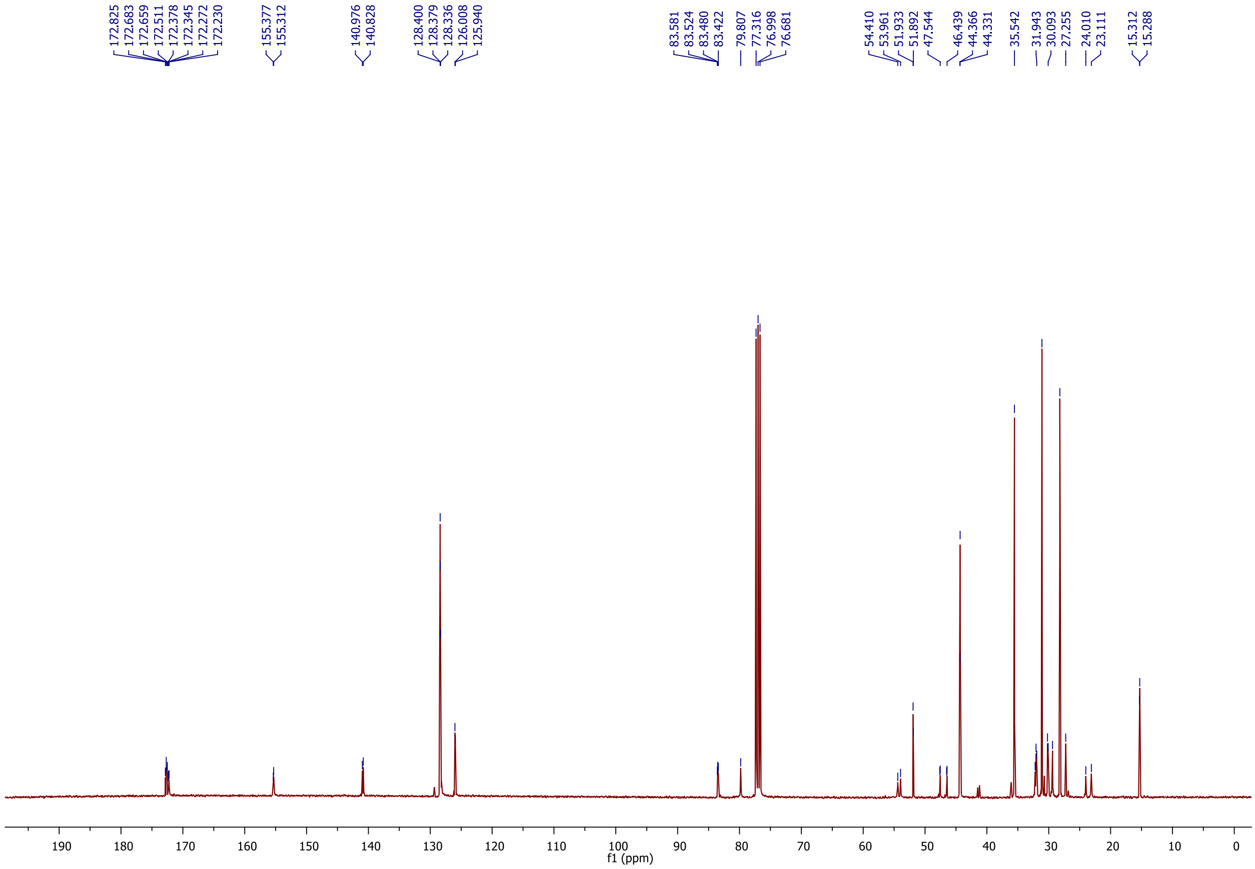

Figure S7. Proton – 1H (top) and carbon – 13C (bottom) NMR spectra for compound **20**

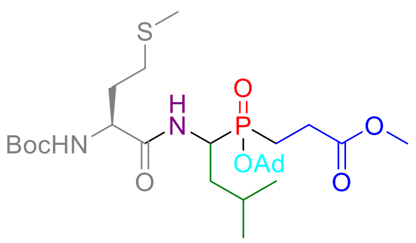

21

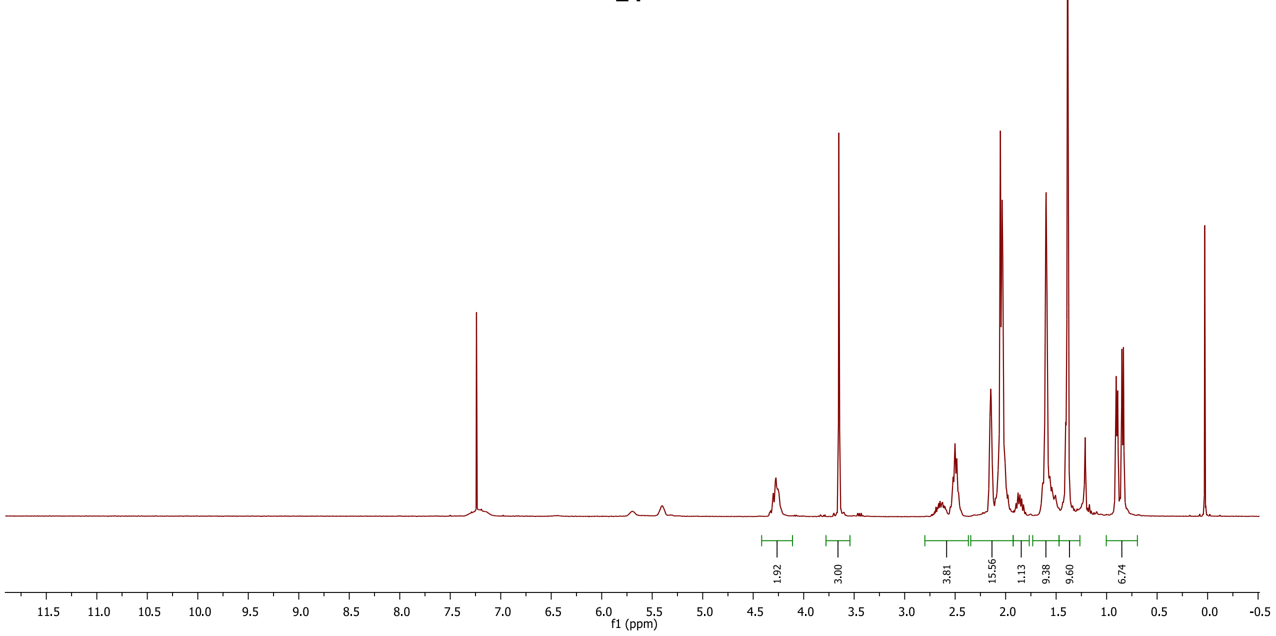

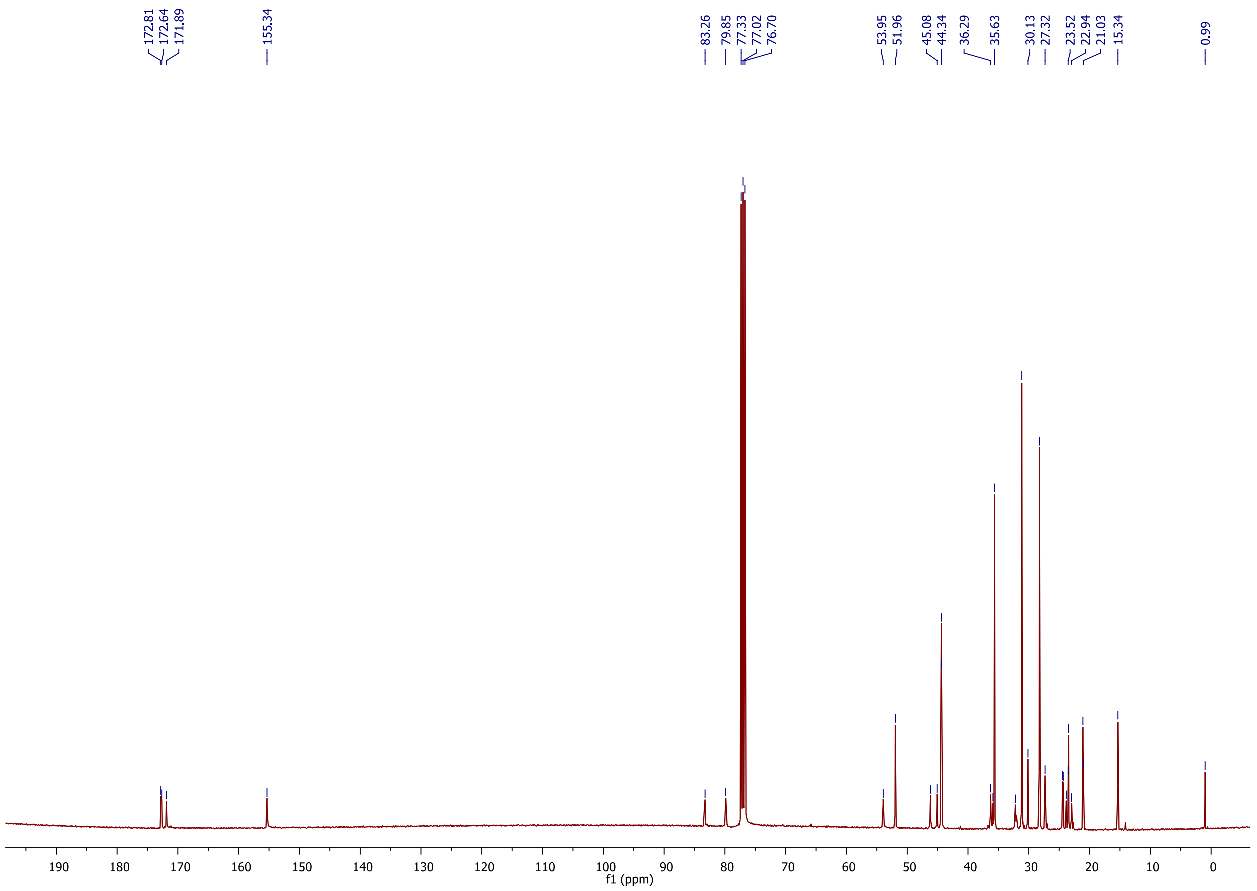

Figure S8. Proton – 1H (top) and carbon – 13C (bottom) NMR spectra for compound **21**

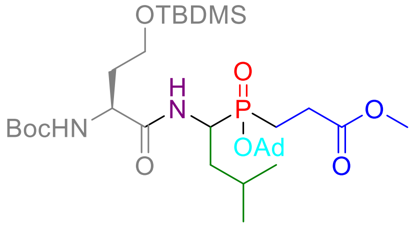

22

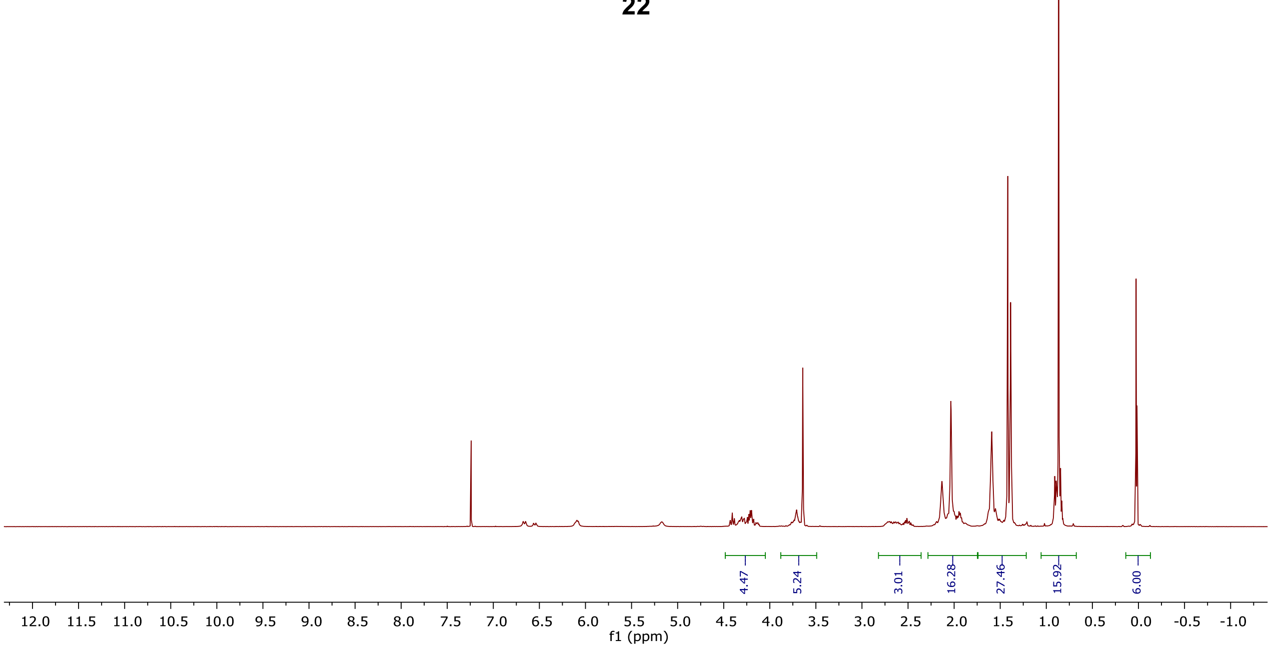

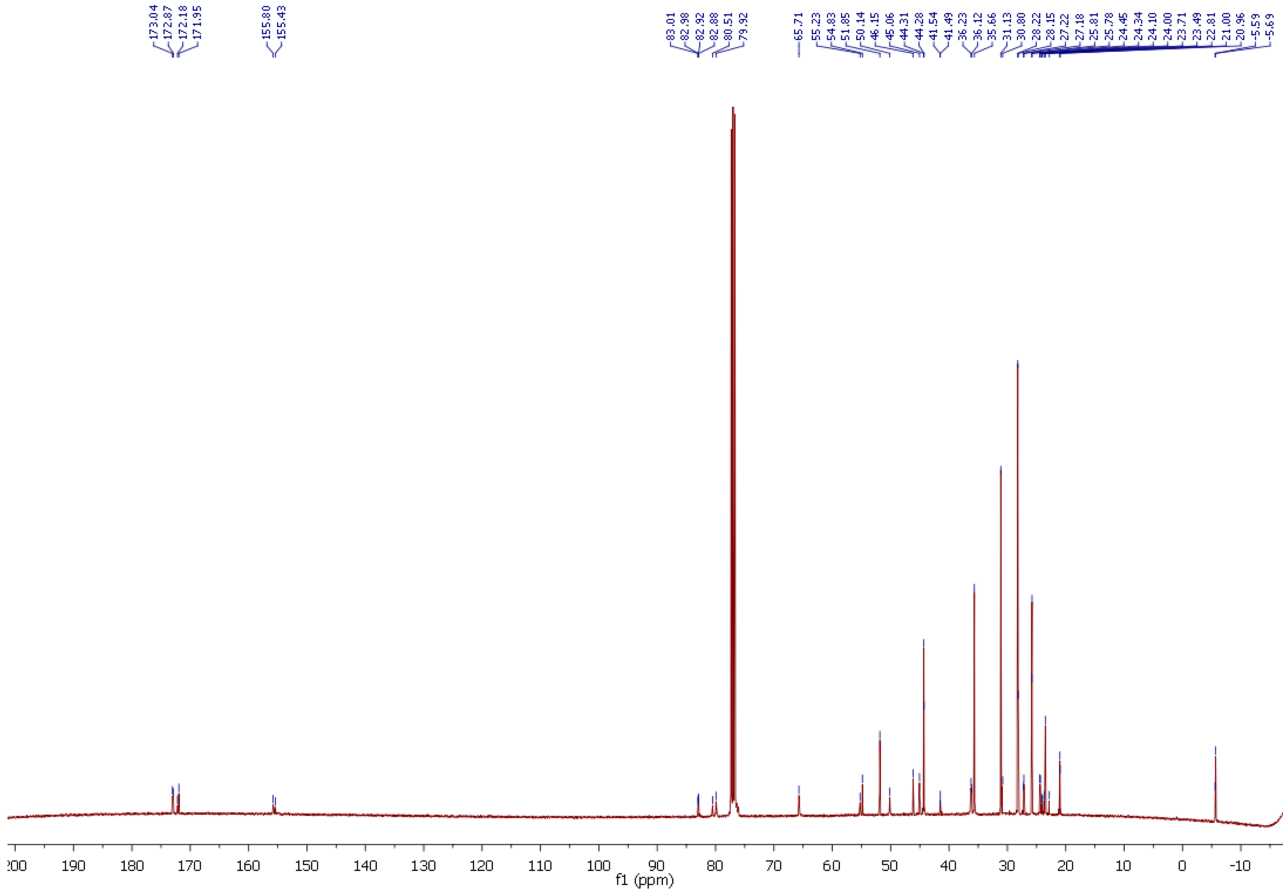

Figure S9. Proton – 1H (top) and carbon – 13C (bottom) NMR spectra for compound **22**

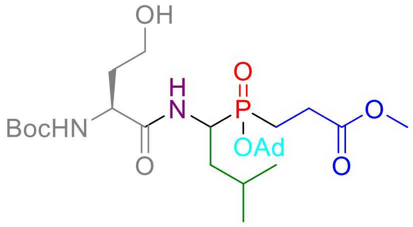

23

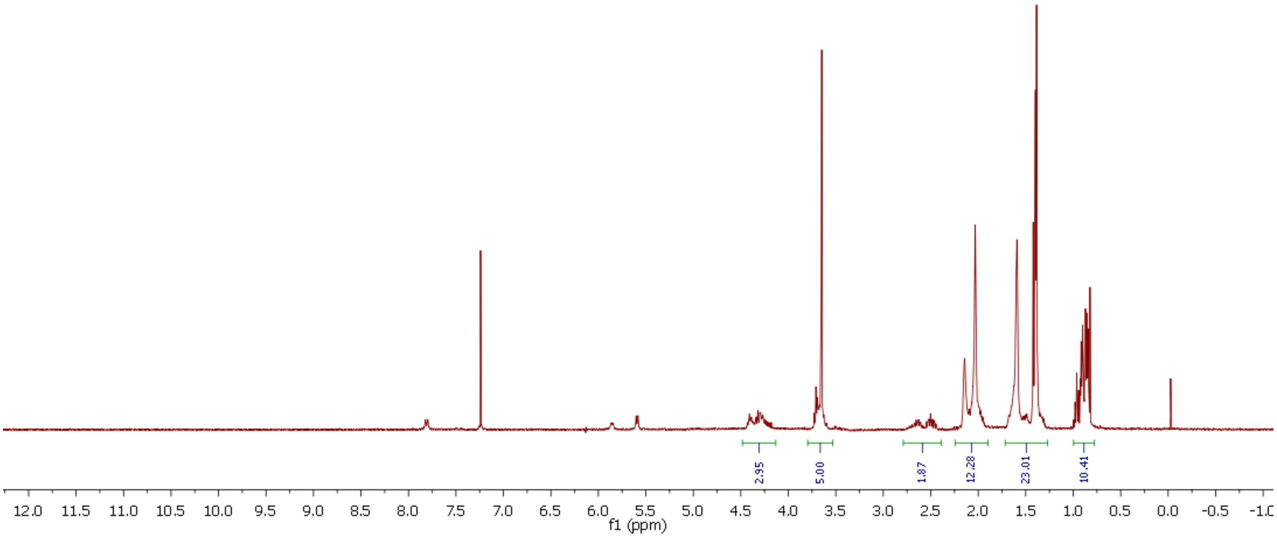

Figure S10. Proton – 1H NMR spectra for compound **23**

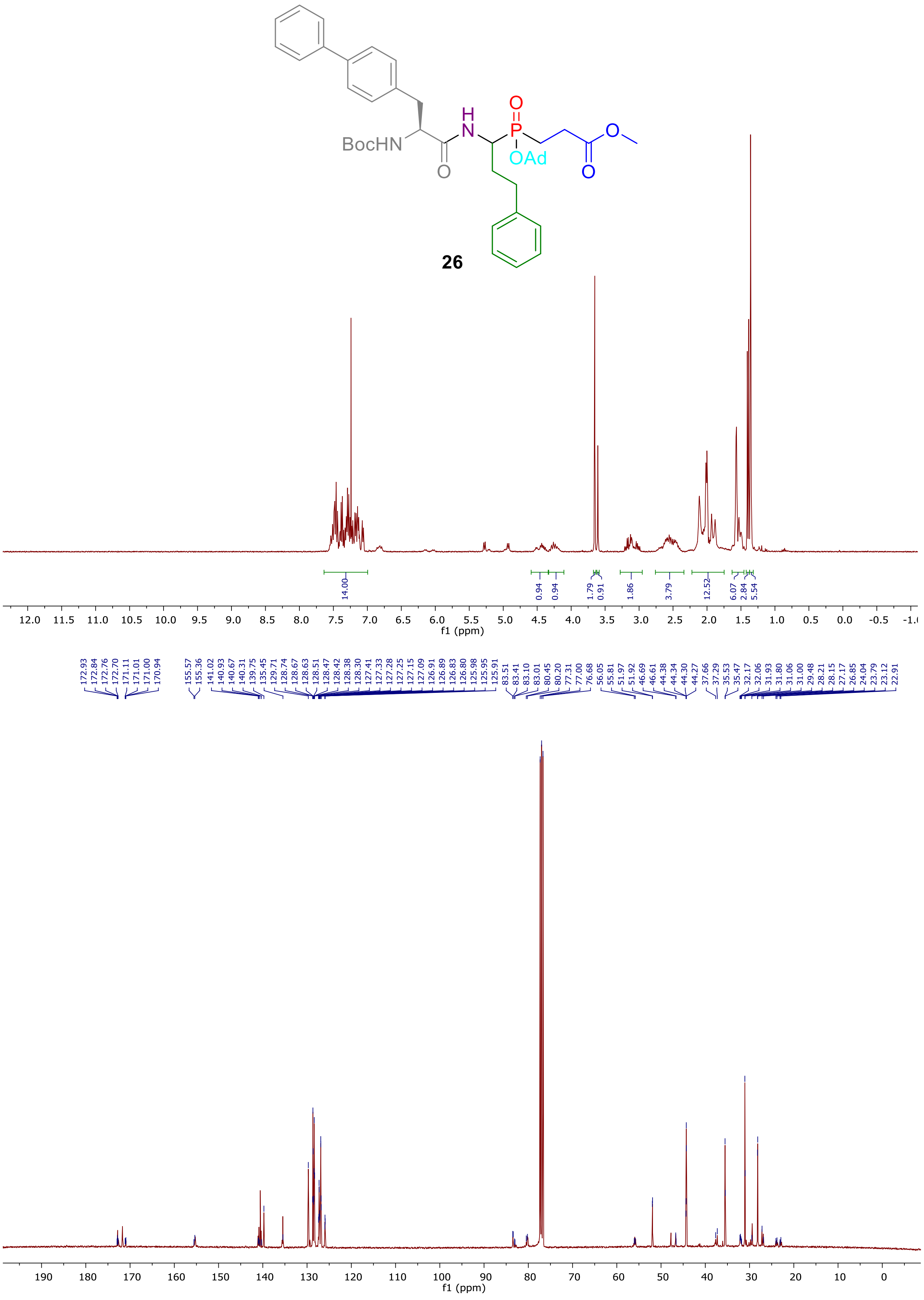

Figure S11. Proton – 1H (top) and carbon – 13C (bottom) NMR spectra for compound **26**

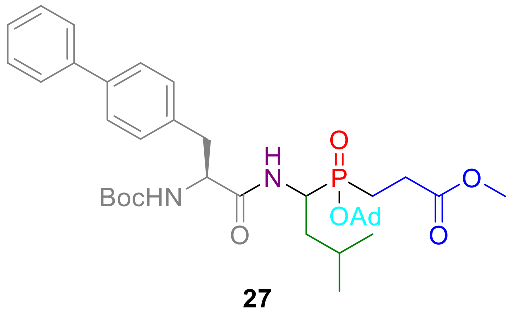

27

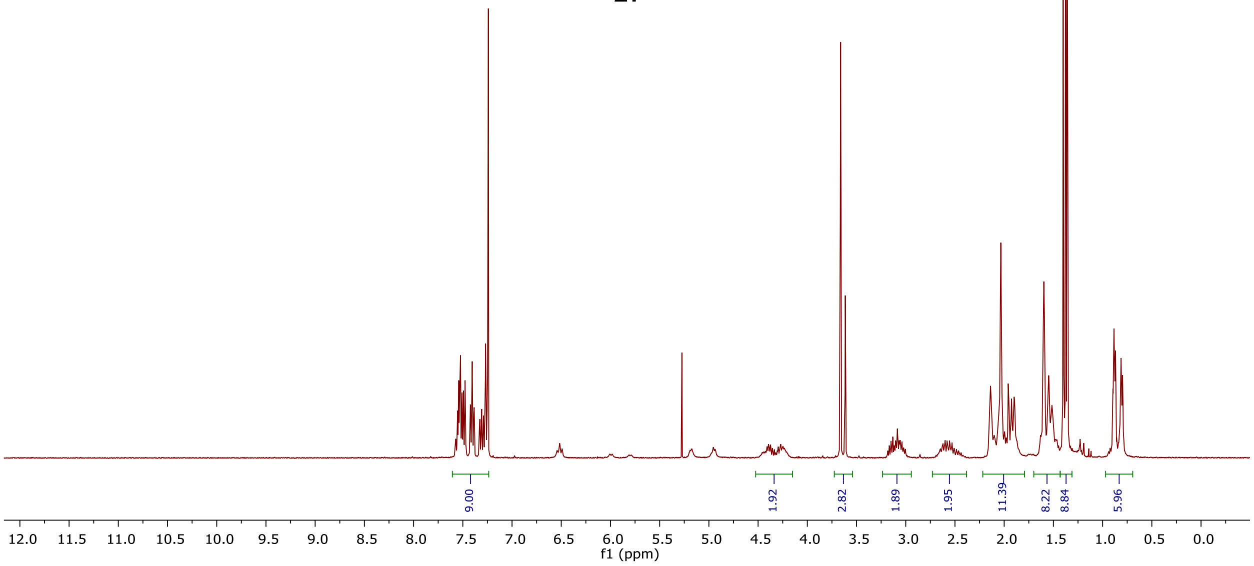

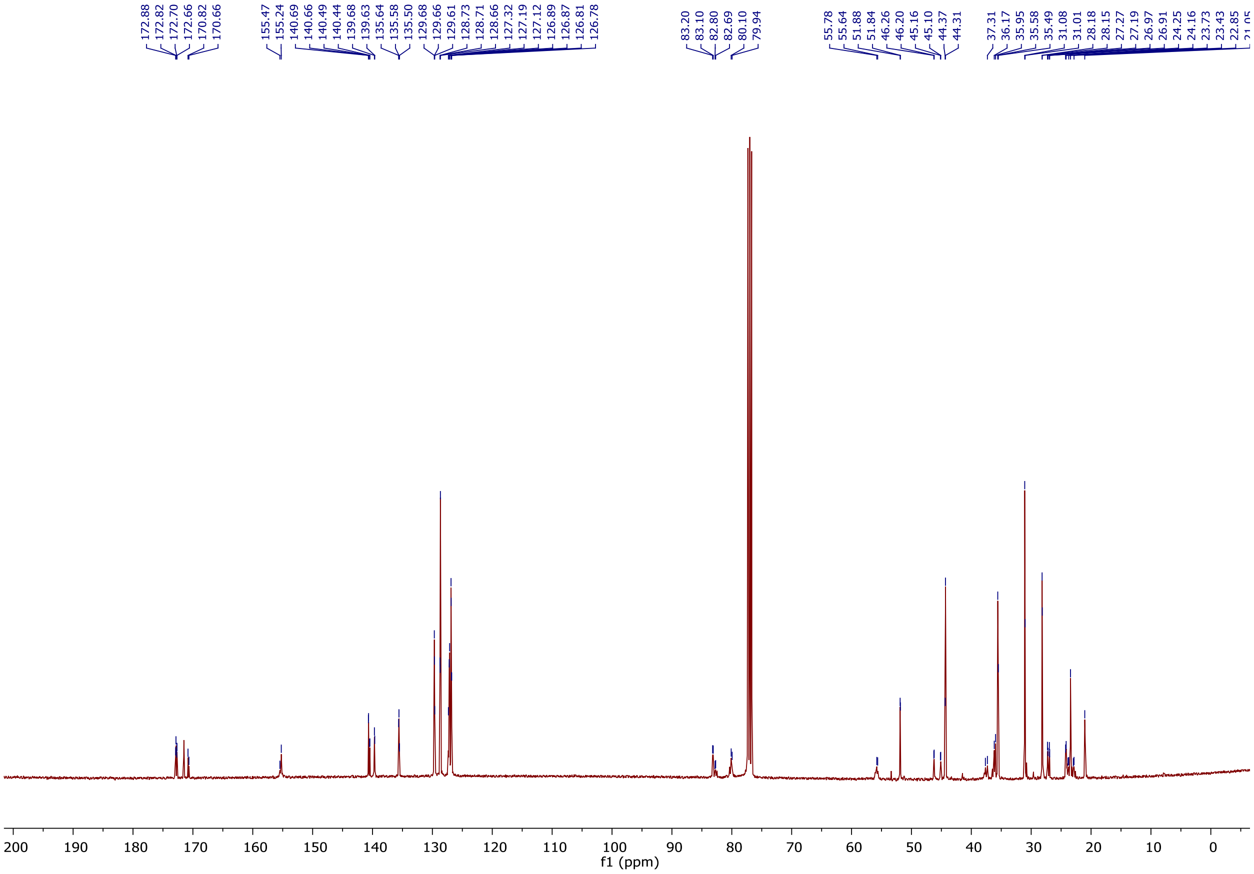

Figure S12. Proton – 1H (top) and carbon – 13C (bottom) NMR spectra for compound **27**

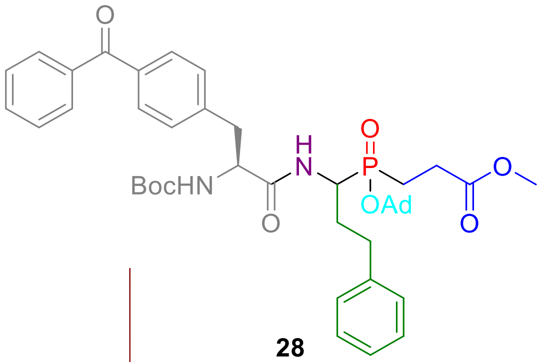

28

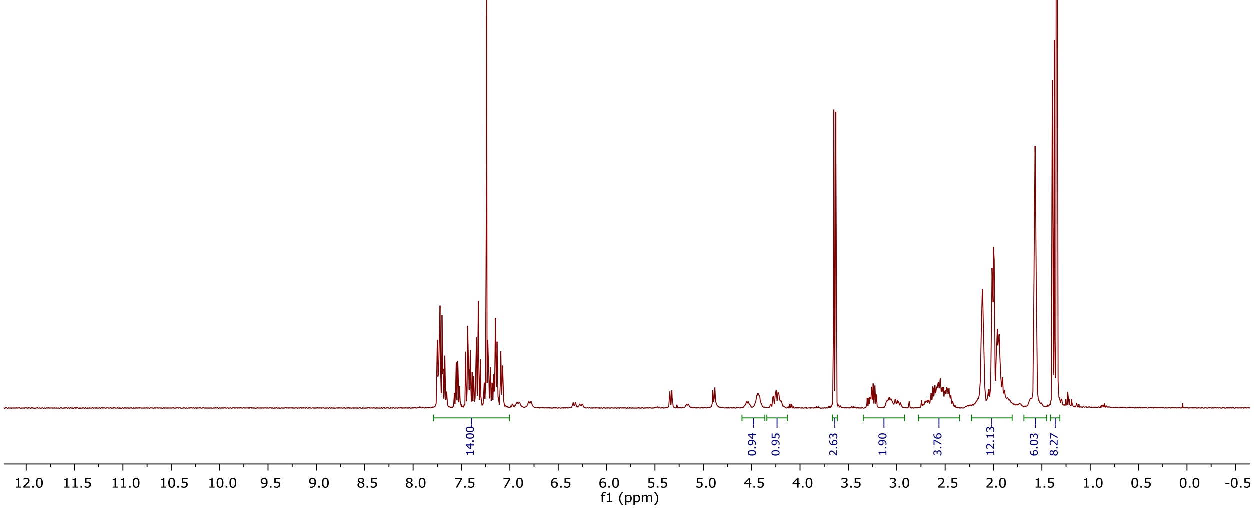

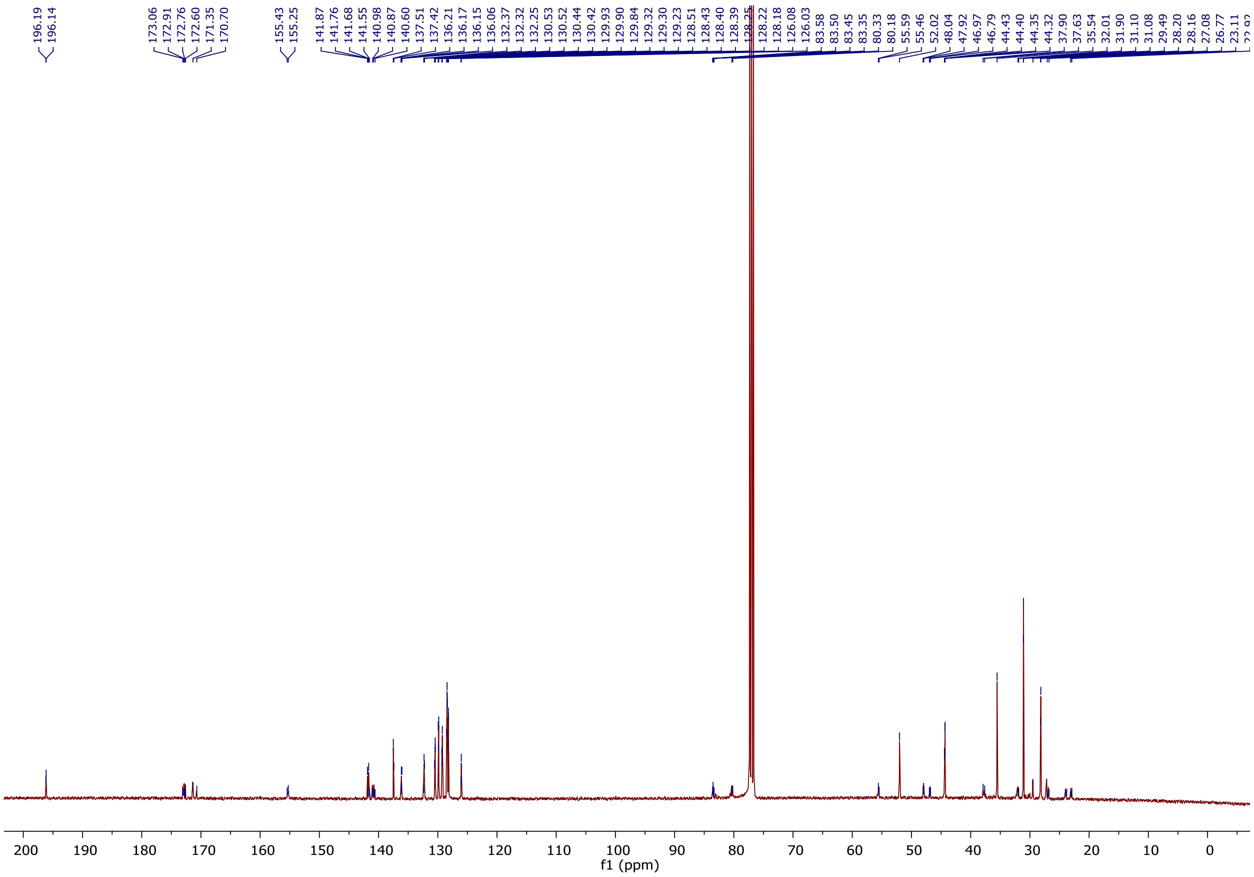

Figure S13. Proton – 1H (top) and carbon – 13C (bottom) NMR spectra for compound **28**

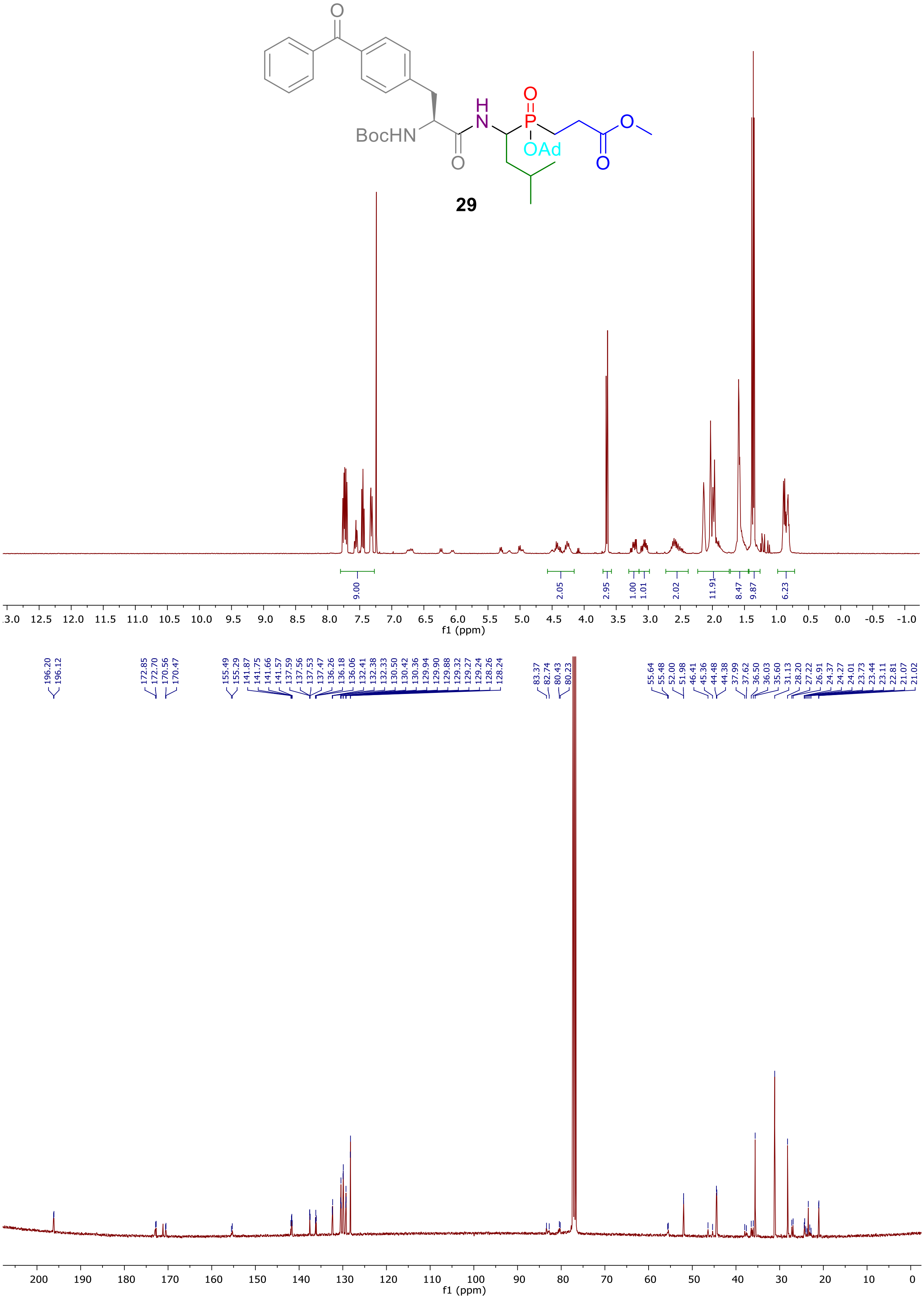

Figure S14. Proton – 1H (top) and carbon – 13C (bottom) NMR spectra for compound **29**

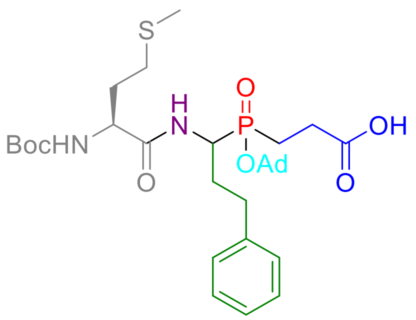

24

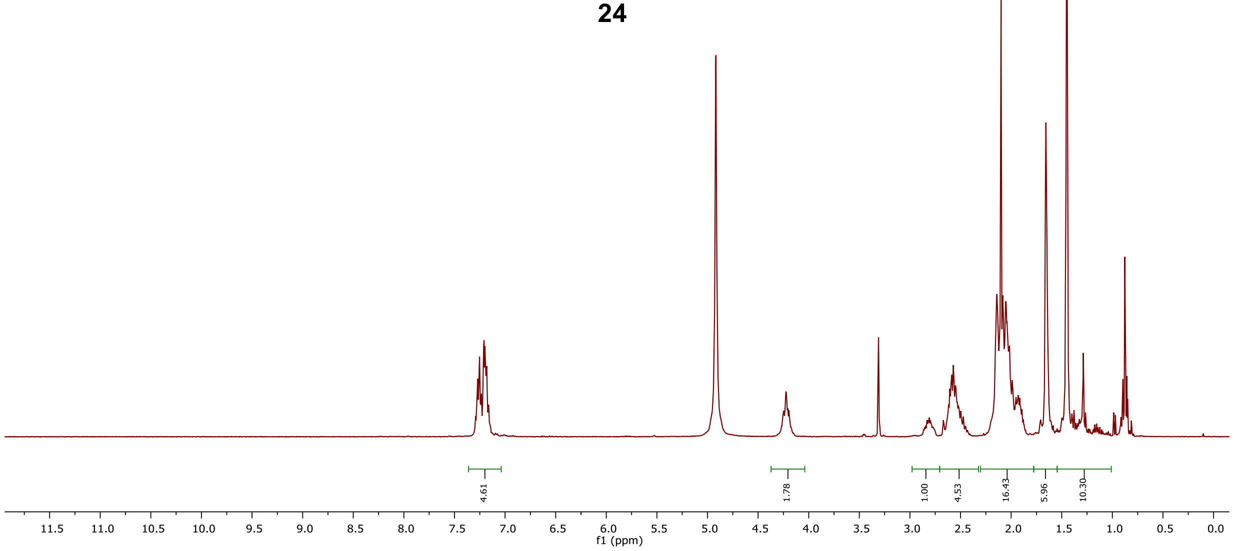

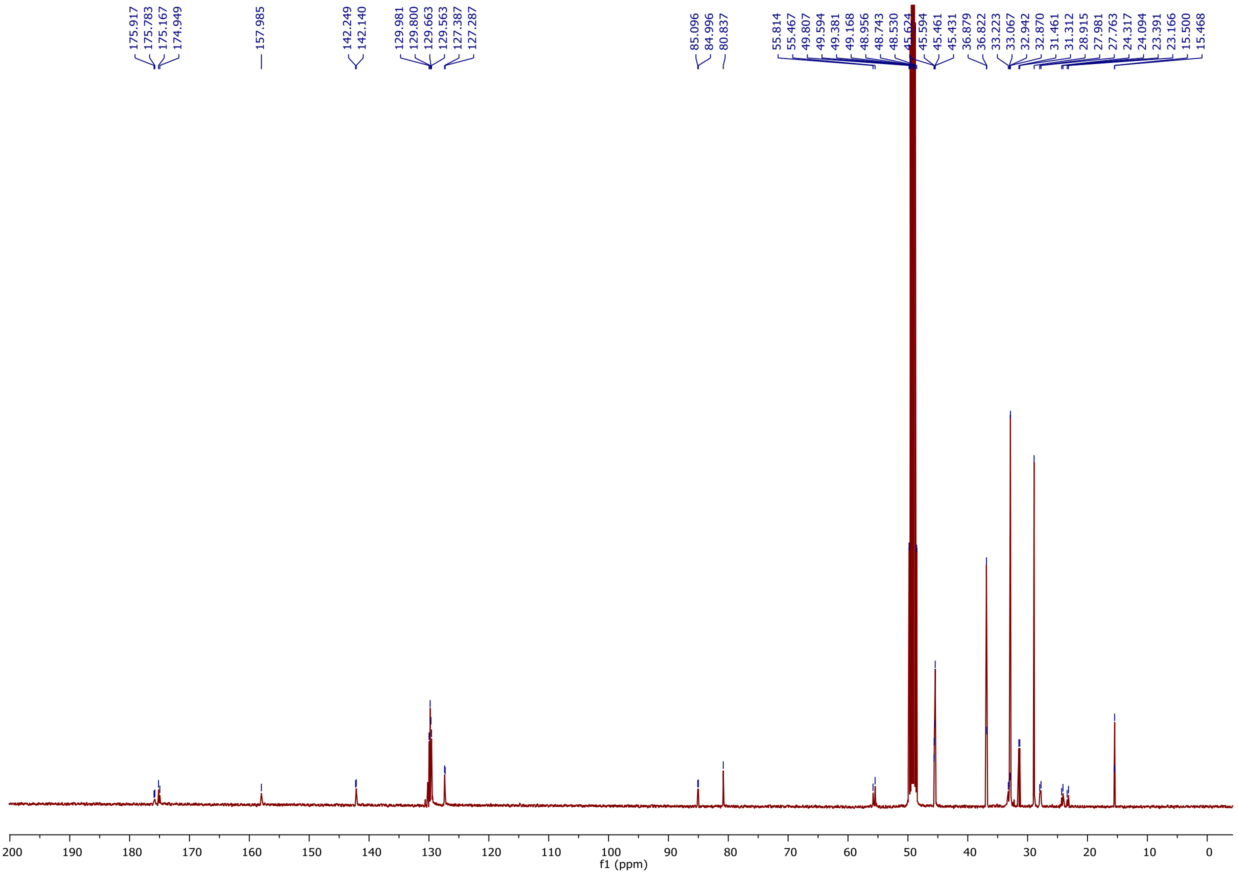

Figure S15. Proton – 1H (top) and carbon – 13C (bottom) NMR spectra for compound **24**

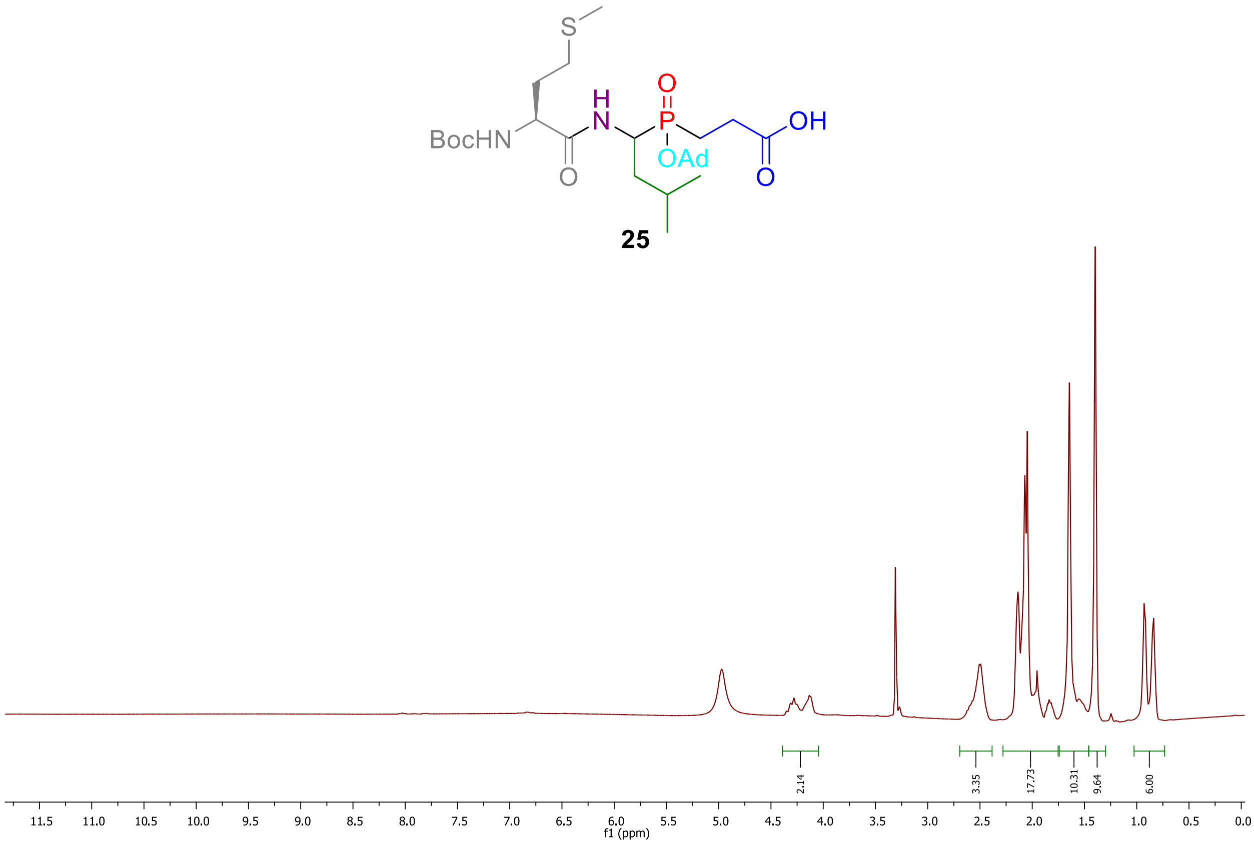

Figure S16. Proton – 1H NMR spectra for compound **25**

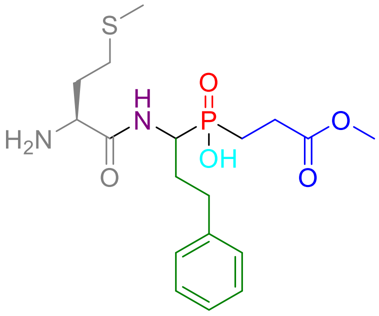

1

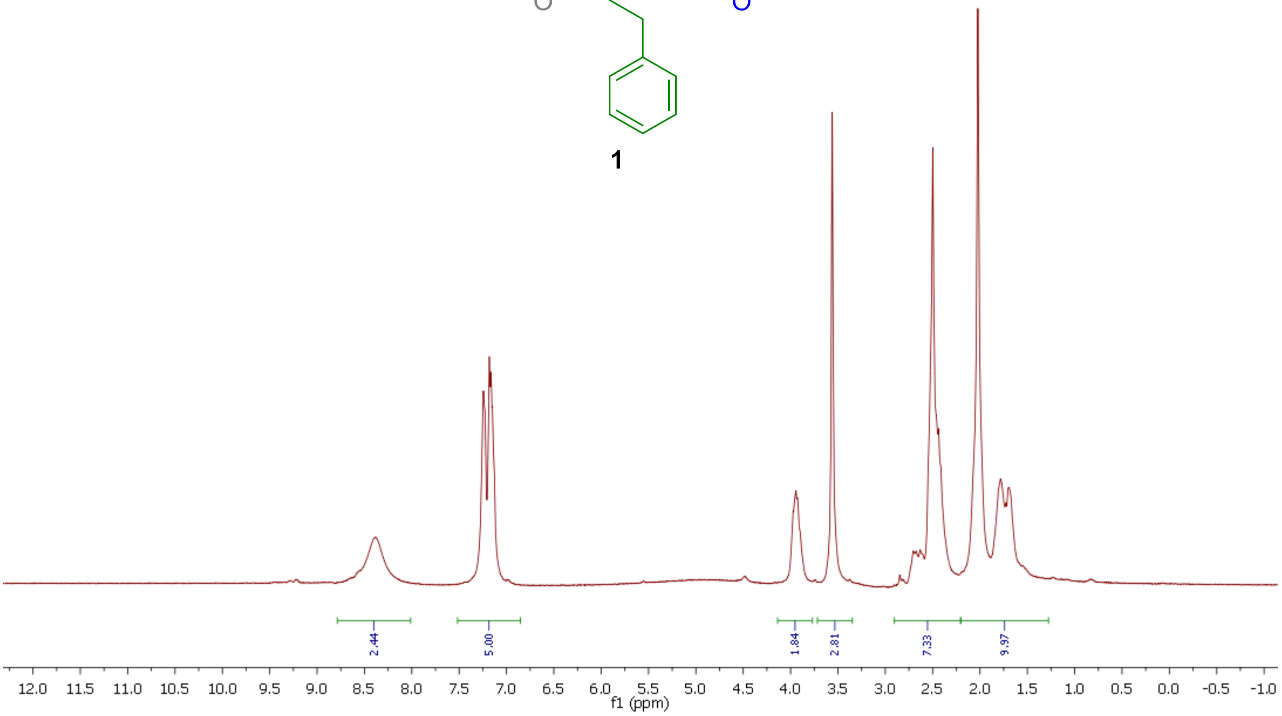

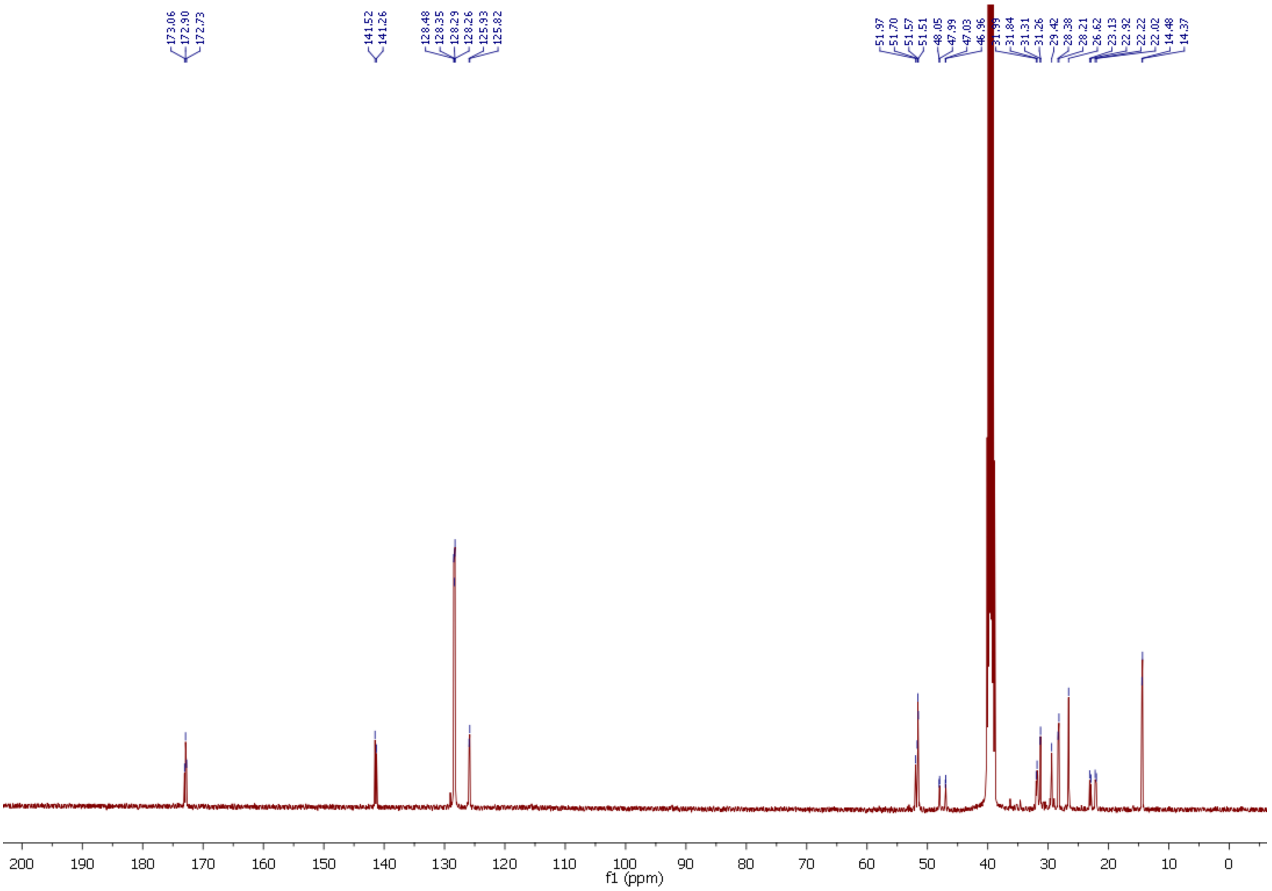

Figure S17. Proton – 1H (top) and carbon – 13C (bottom) NMR spectra for compound **1**

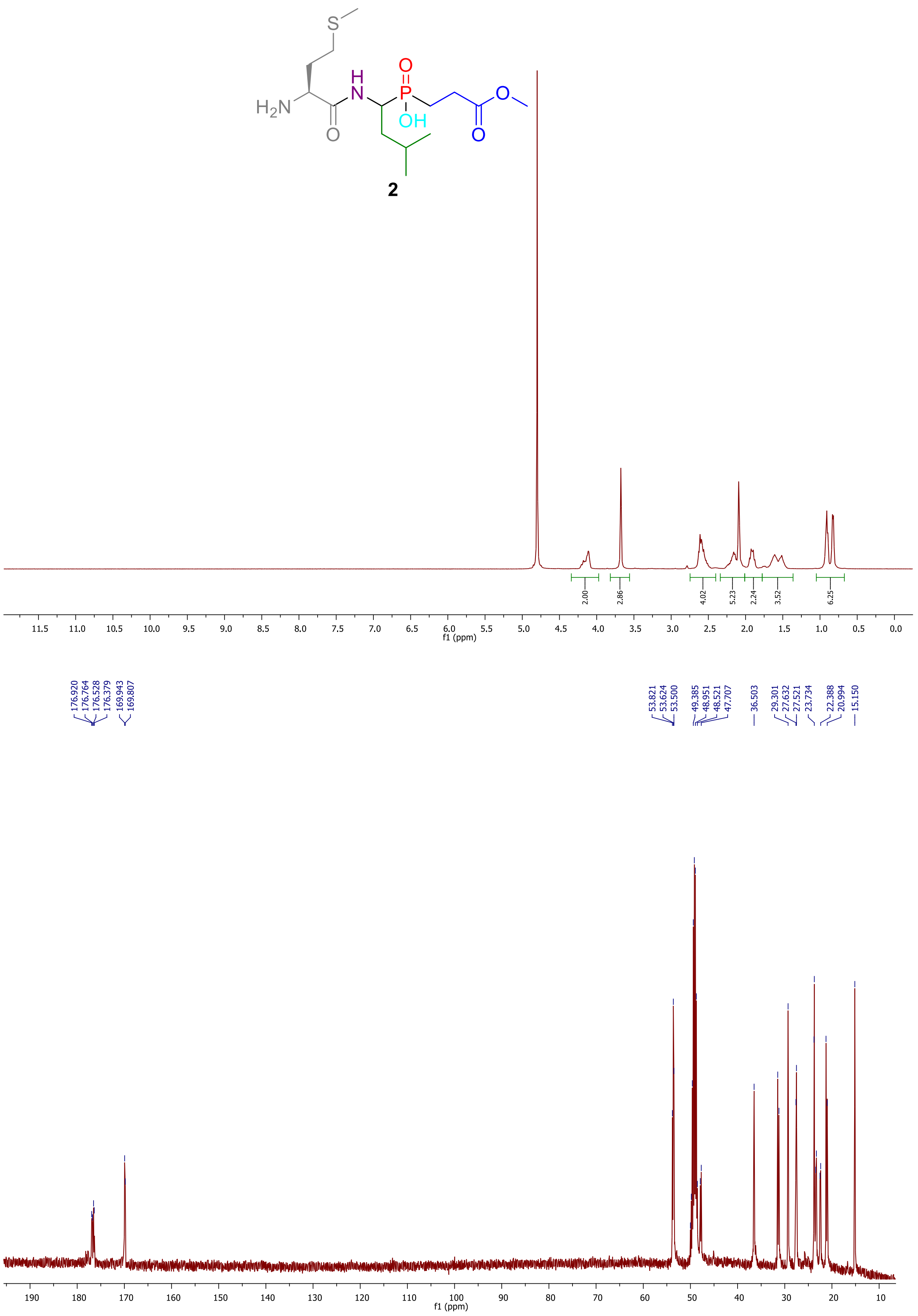

Figure S18. Proton – 1H (top) and carbon – 13C (bottom) NMR spectra for compound **2**

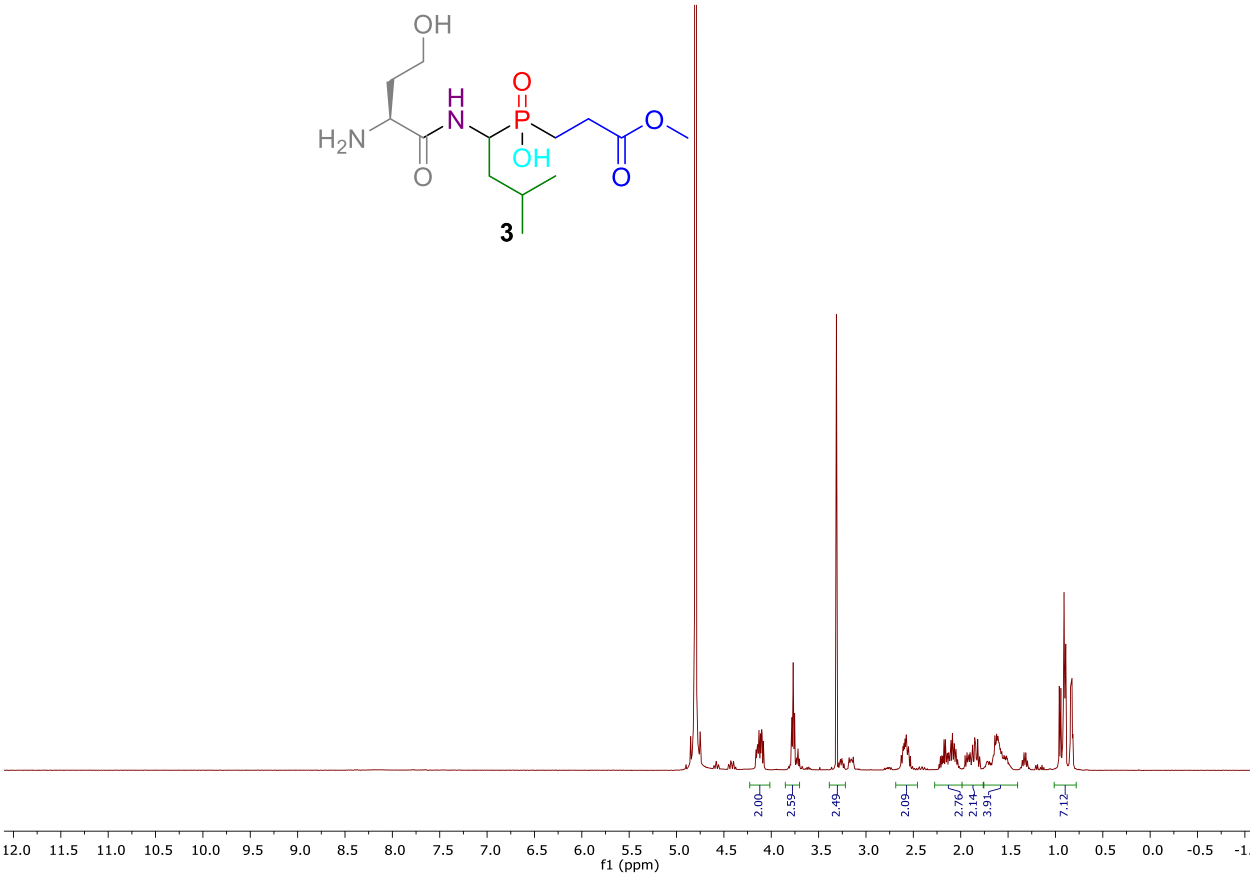

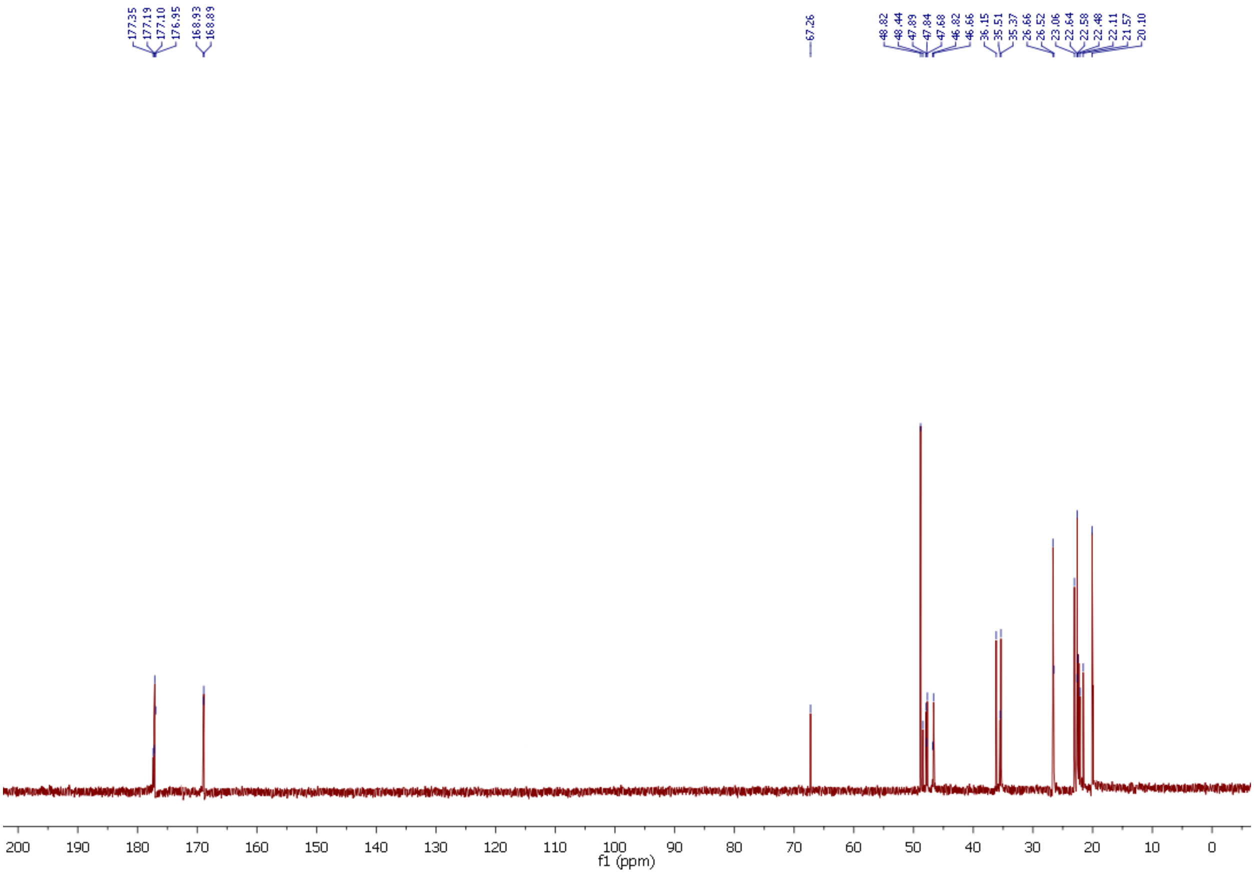

Figure S19. Proton – 1H (top) and carbon – 13C (bottom) NMR spectra for compound **3**

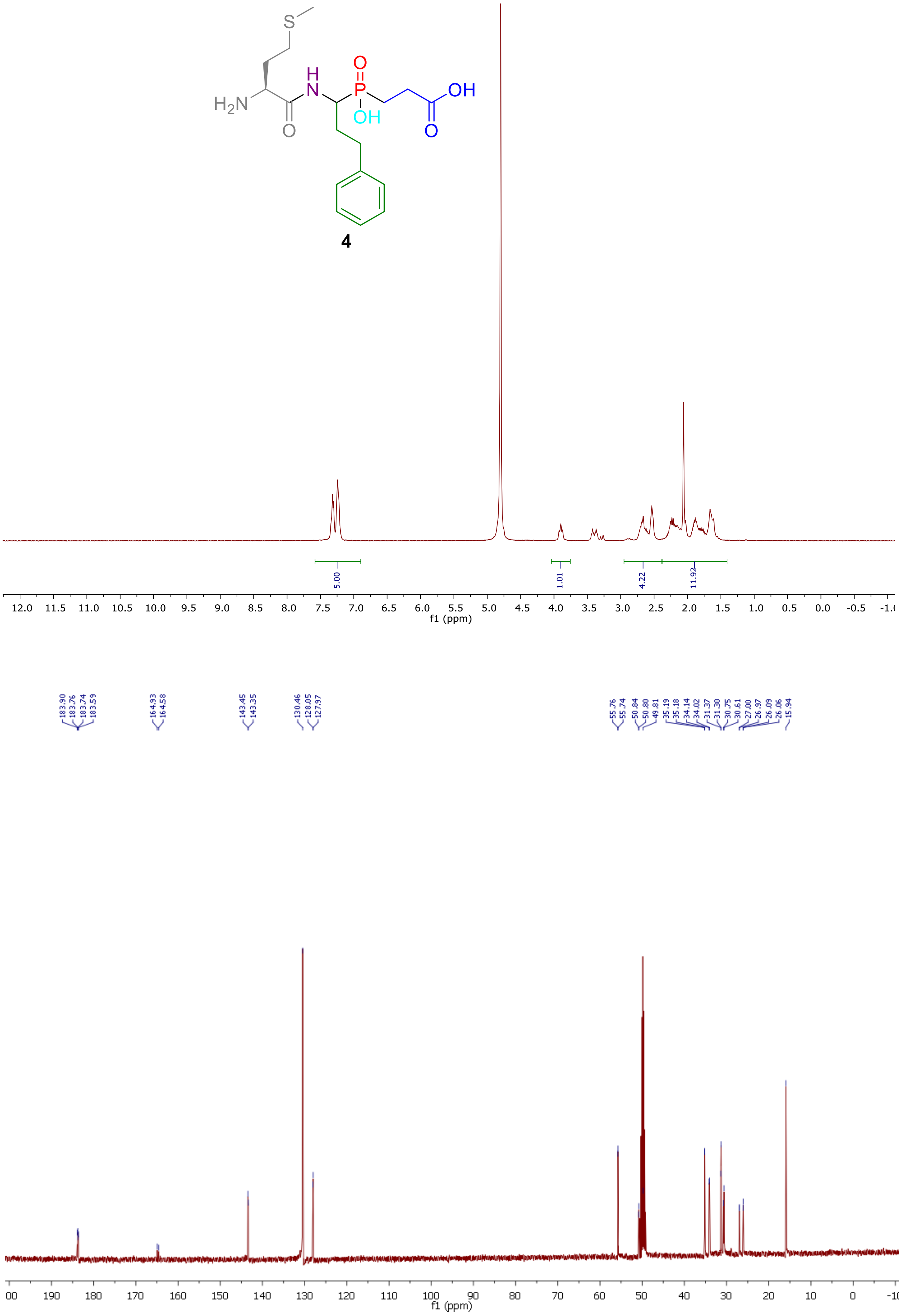

Figure S20. Proton – 1H (top) and carbon – 13C (bottom) NMR spectra for compound **4**

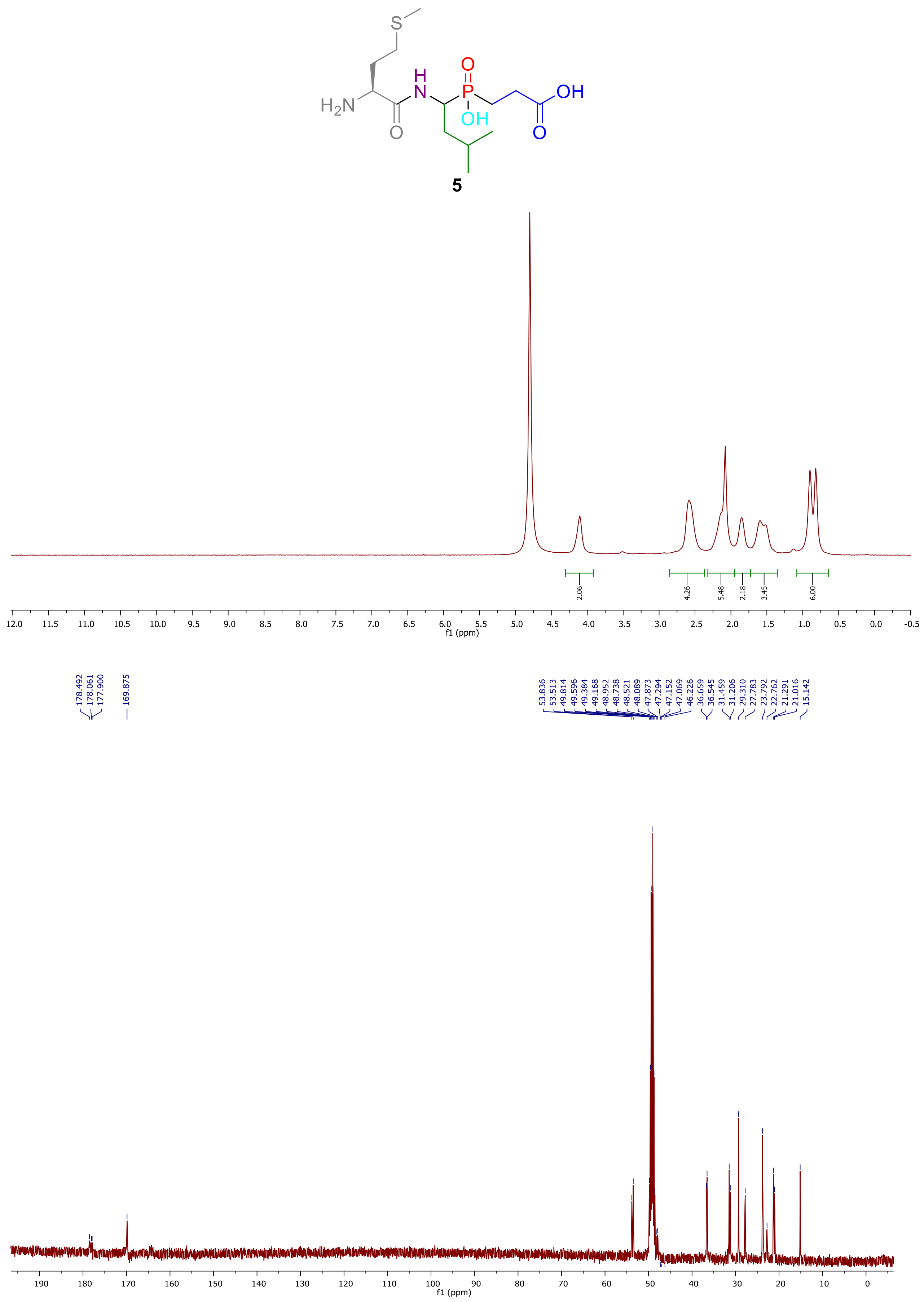

Figure S21. Proton – 1H (top) and carbon – 13C (bottom) NMR spectra for compound **5**

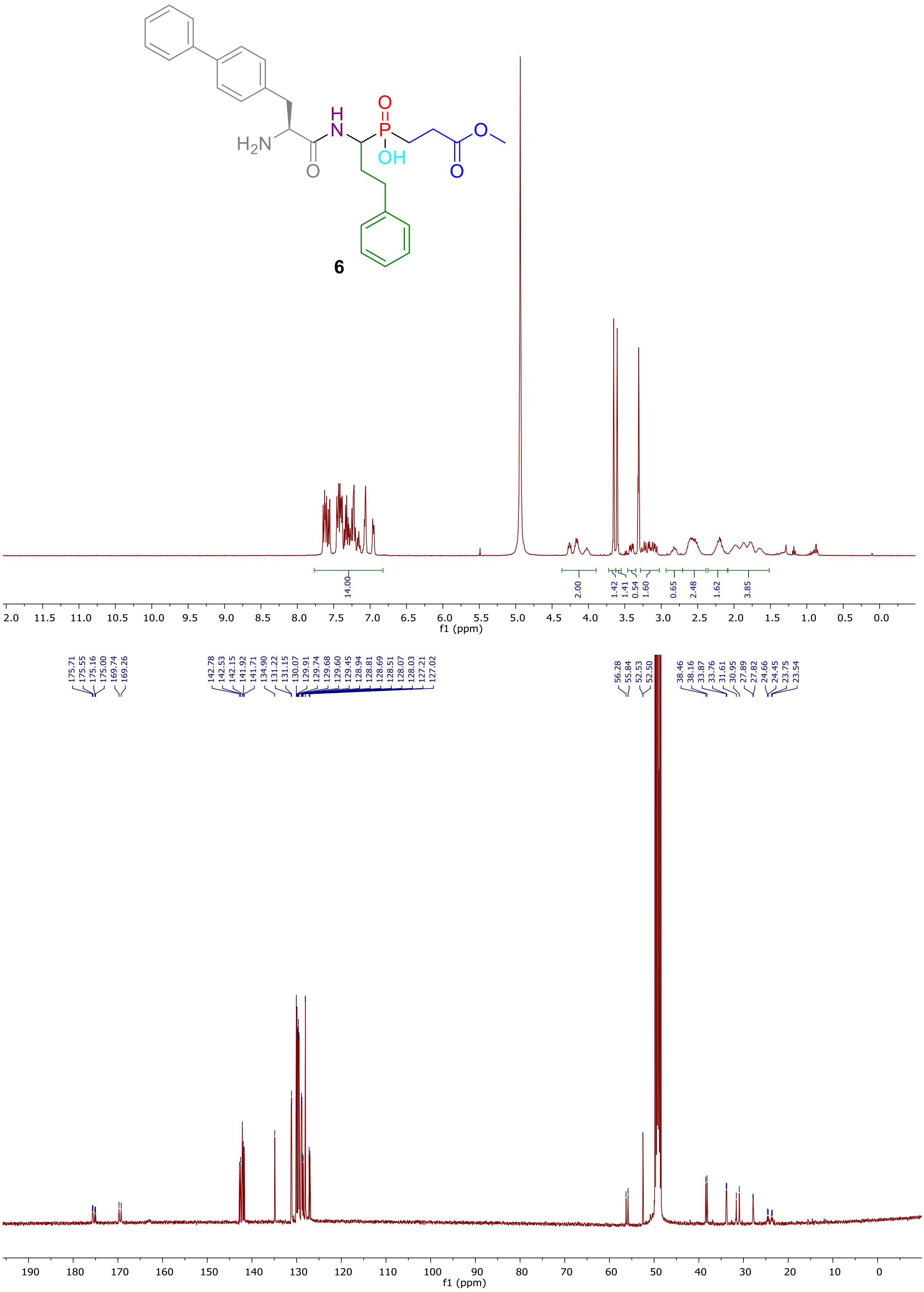

Figure S22. Proton – 1H (top) and carbon – 13C (bottom) NMR spectra for compound **6**

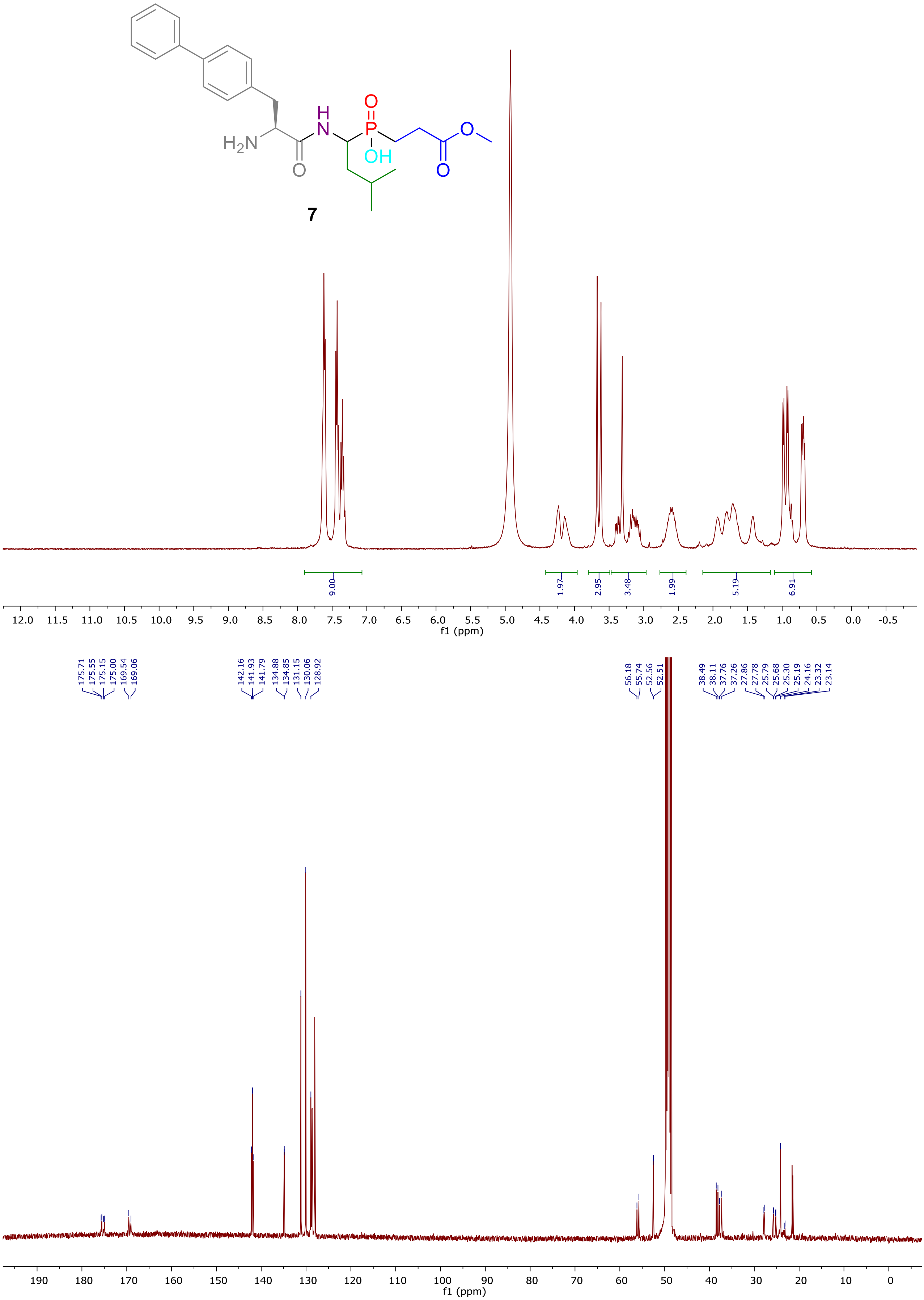

Figure S23. Proton – 1H (top) and carbon – 13C (bottom) NMR spectra for compound **7**

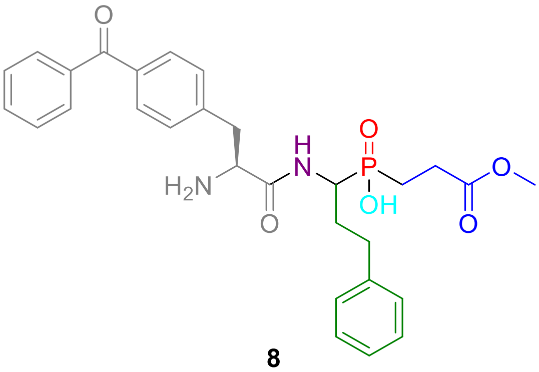

8

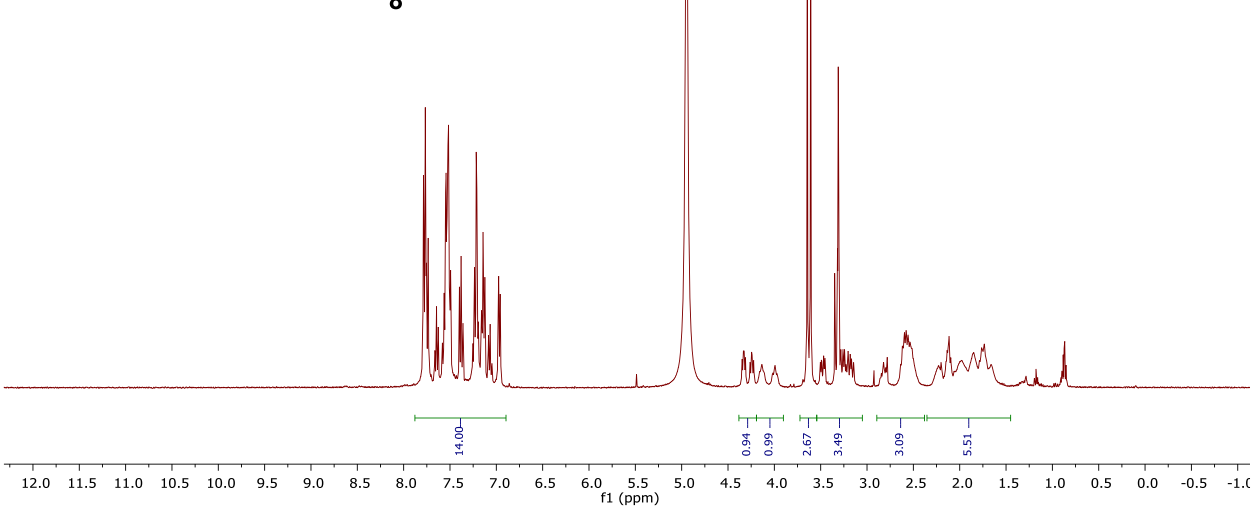

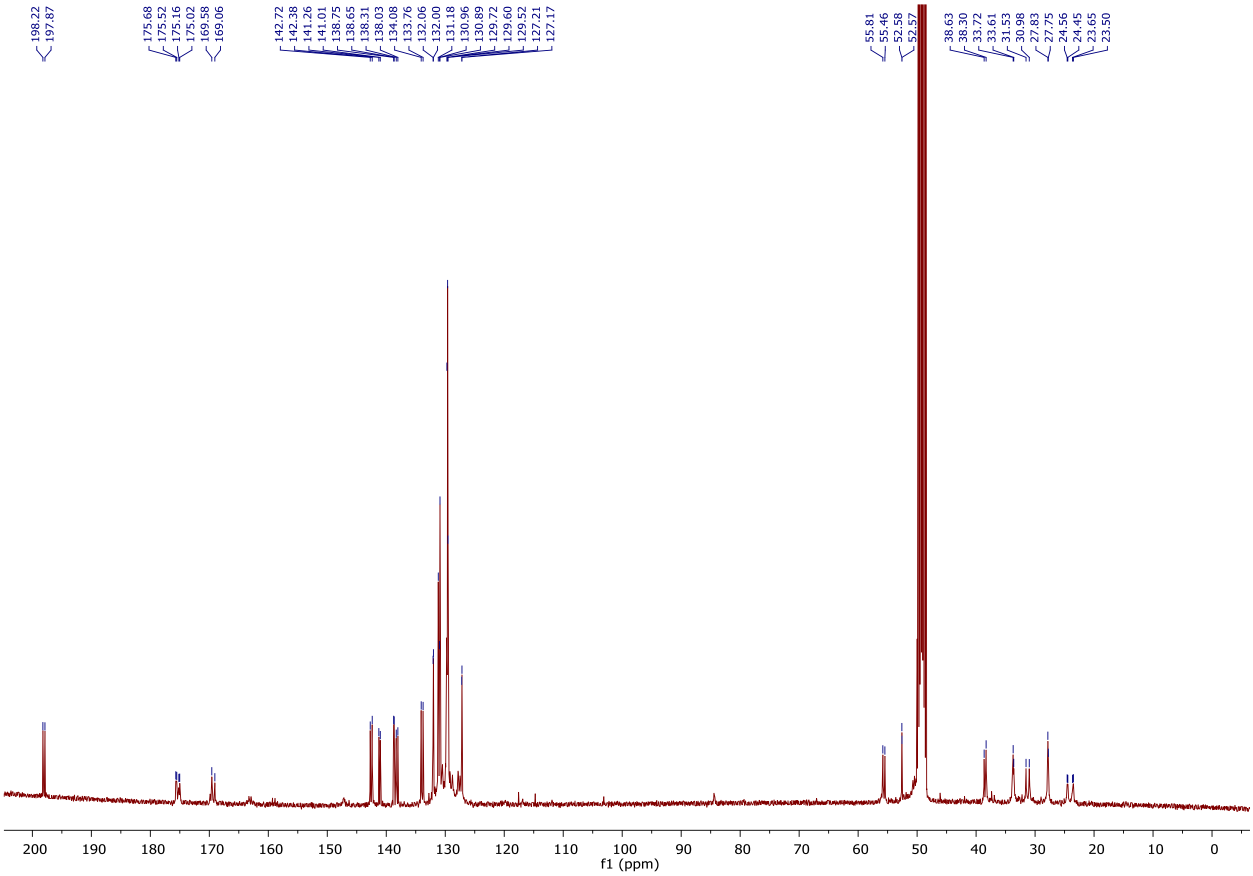

Figure S24. Proton – 1H (top) and carbon – 13C (bottom) NMR spectra for compound **8**

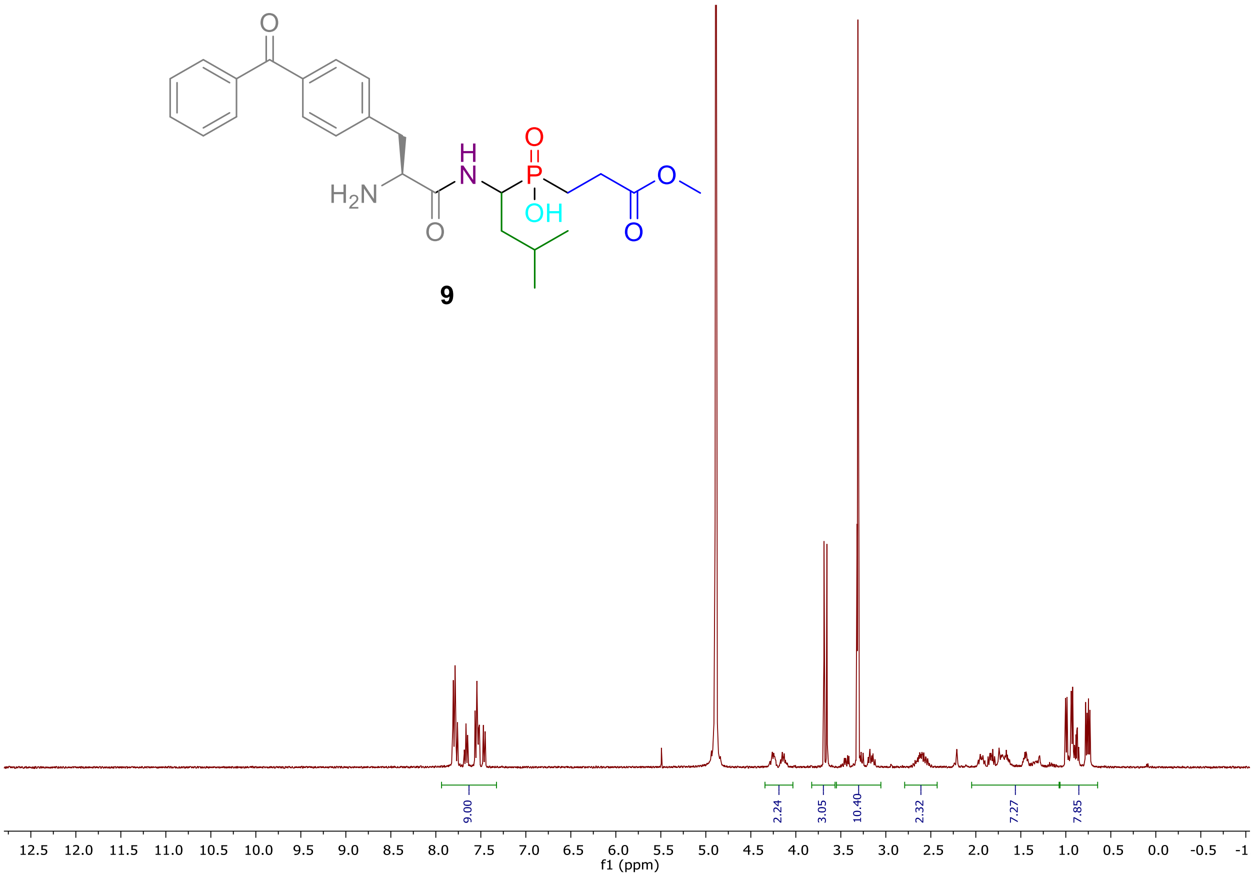

9

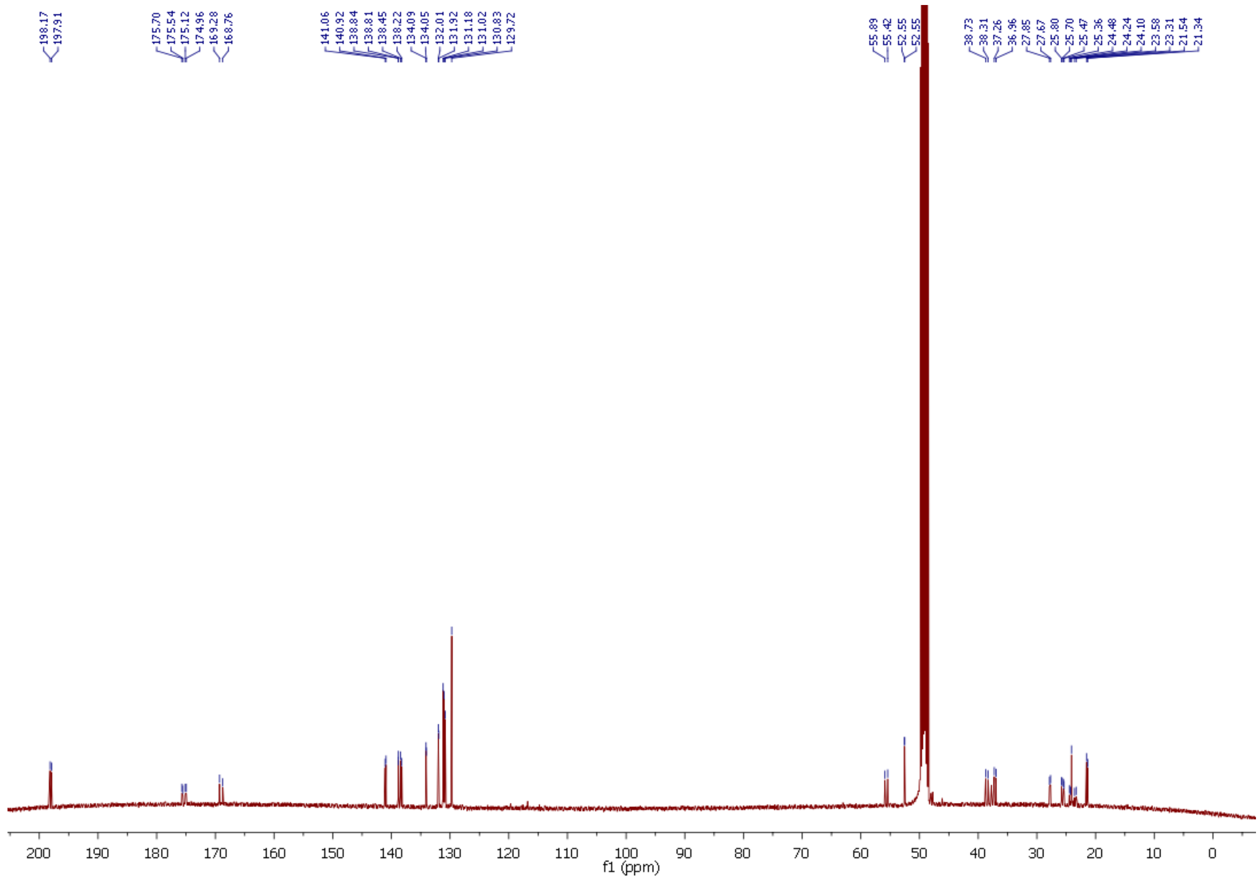

Figure S25. Proton – 1H (top) and carbon – 13C (bottom) NMR spectra for compound **9**

Table S1. Dixon plots for investigated compounds in the presence of *SsLAP*

Entry	Structure	DIXON PLOT
1	Met-hPheP[CH_2]Gly-OMe	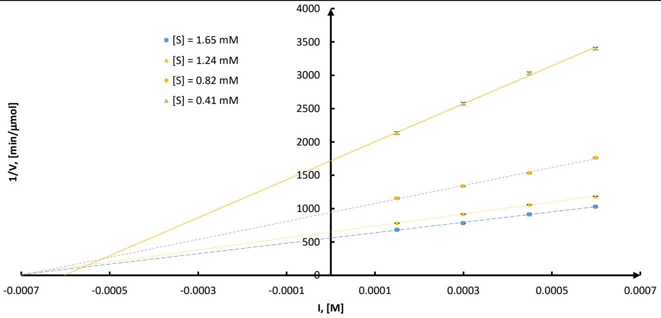
2	Met-LeuP[CH_2]Gly-OMe	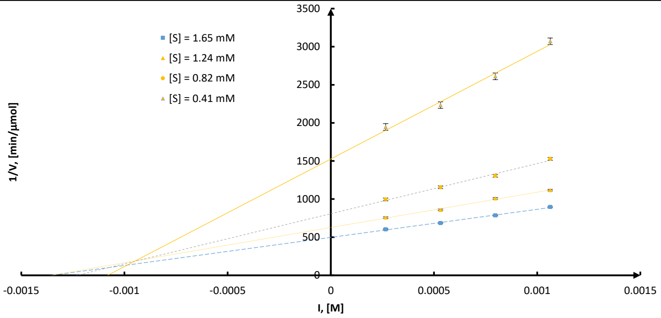
4	Met-hPheP[CH_2]Gly	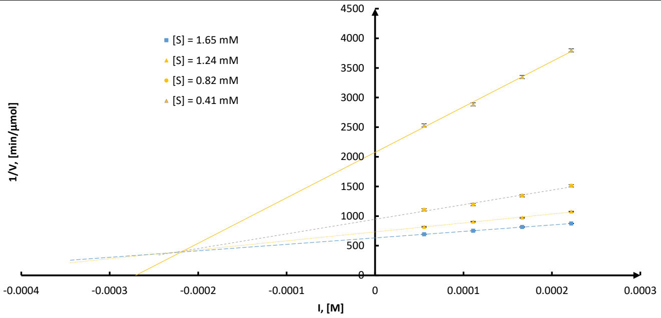
5	Met-LeuP[CH_2]Gly	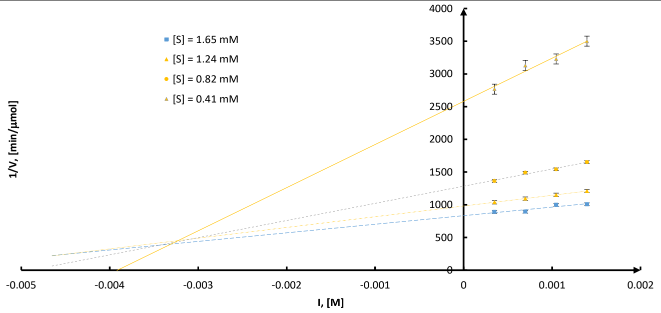

6 Bip-hPheP[CH_2]Gly-OMe

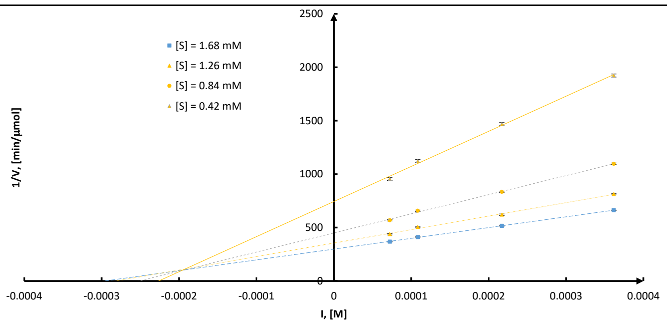

7 Bip-LeuP[CH_2]Gly-OMe

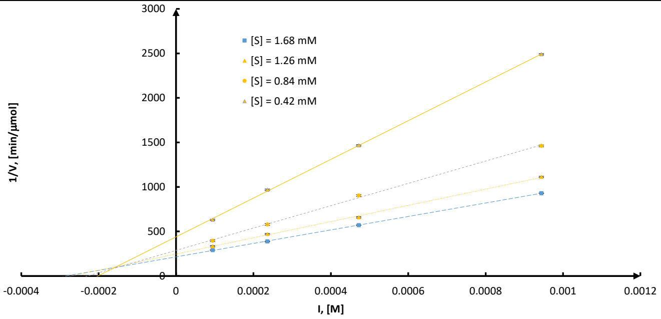

8 Bpa-hPheP[CH_2]Gly-OMe

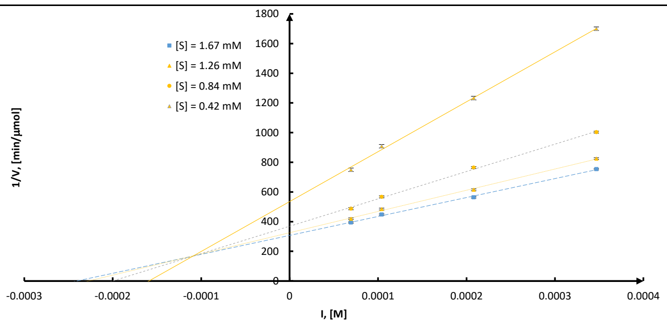

9 Bpa-LeuP[CH_2]Gly-OMe

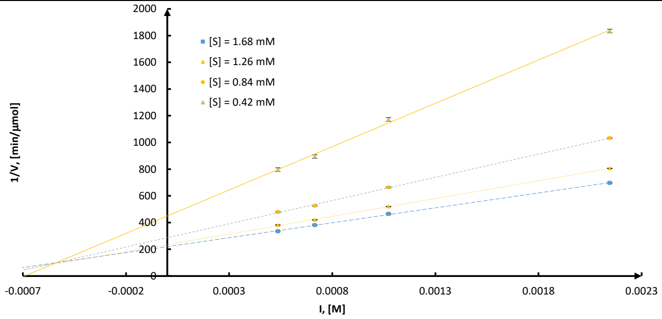

Table S2. Dixon plots for investigated compounds in the presence of *HvLAP*

Entry		DIXON PLOT
2	Met-LeuP[CH_2]Gly-OMe	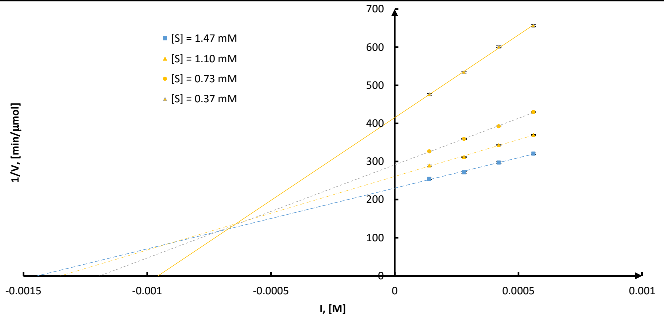
3	Ser-LeuP[CH_2]Gly-OMe	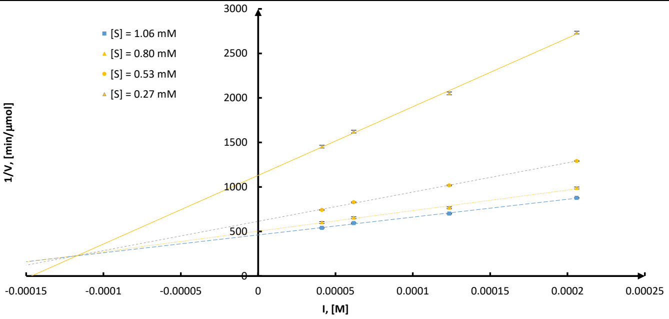
4	Met-hPheP[CH_2]Gly	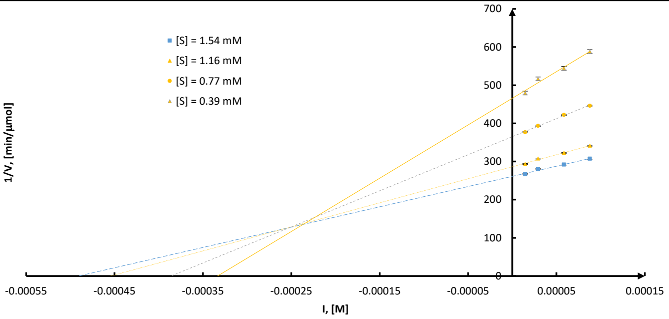
5	Met-LeuP[CH_2]Gly	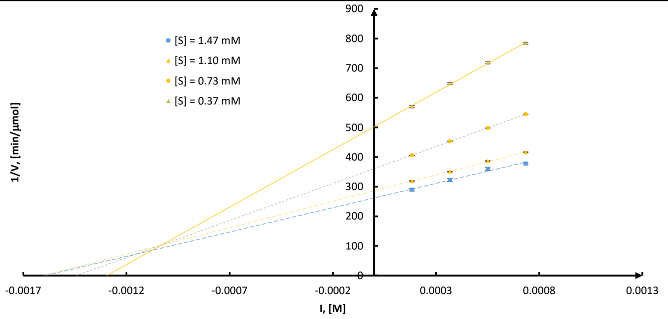

6 Bip-hPheP[CH_2]Gly-OMe

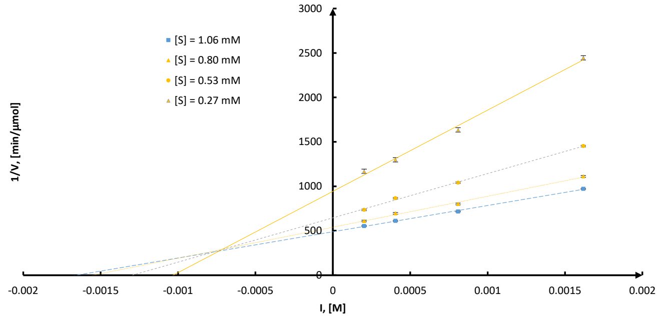

7 Bip-LeuP[CH_2]Gly-OMe

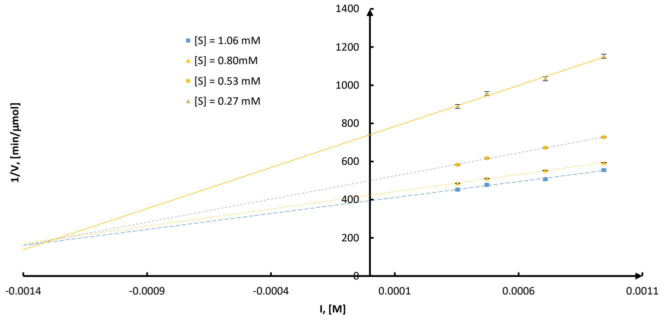

8 Bpa-hPheP[CH_2]Gly-OMe

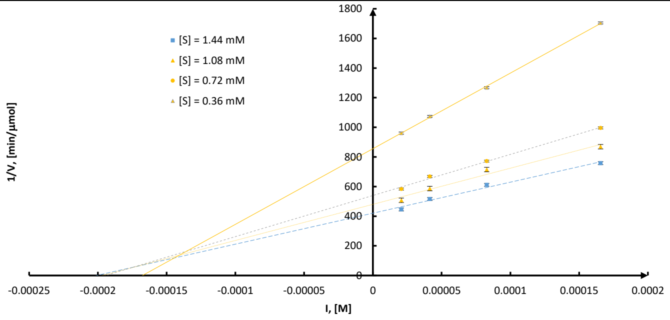

9 Bpa-LeuP[CH_2]Gly-OMe

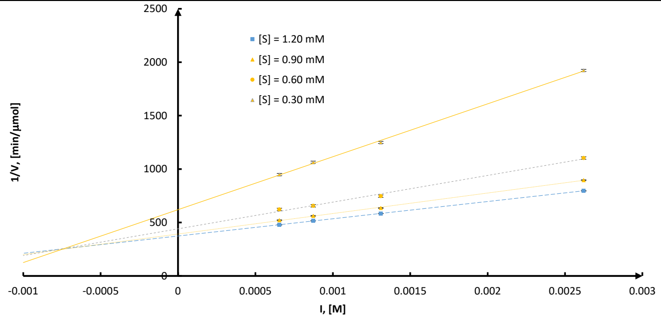

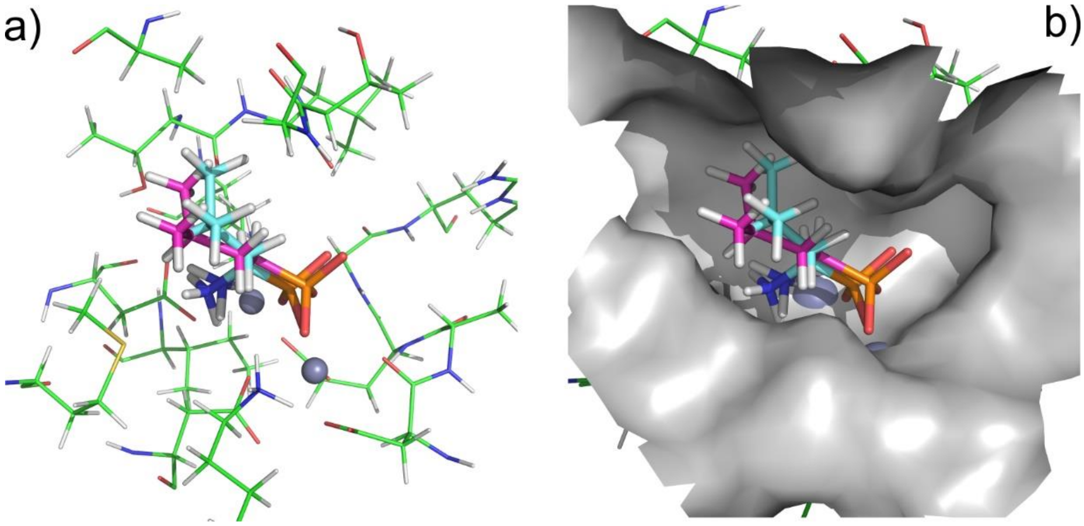

Figure S26 Comparison of alignments of $LeuPO_3H_2$ in the active site of bovine lens LAP in crystal structure (magenta) (PDB ID: 1LCP) and docked with GOLD software (cyan). Zinc ions shown as gray spheres. RMSD value between docked and experimental structures is 0.199.

Table S3. GoldScore Fitness values for the investigated compounds 1-9 and mammalian and plant aminopeptidases.

Entry		**Structure**	**Isomers**	**GoldScore Fitness**	
				BtLAP	*SlAP*
1	Met-hPheP[CH_2]Gly-OMe	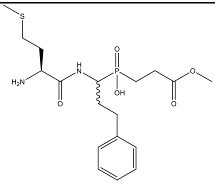	*SR*	79.5645	82.4937
			SS	87.4958	87.7622
2	Met-LeuP[CH_2]Gly-OMe	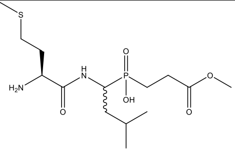	*SR*	78.6926	83.5358
			SS	75.8903	82.1290
3	Ser-LeuP[CH_2]Gly-OMe	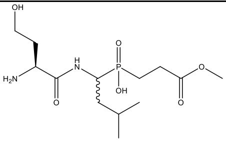	*SR*	68.6116	87.1260
			SS	77.7755	75.2536
4	Met-hPheP[CH_2]Gly	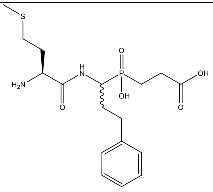	*SR*	101.6310	105.2040
			SS	100.4380	108.3880
5	Met-LeuP[CH_2]Gly	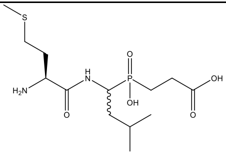	*SR*	93.8243	102.2040
			SS	100.1070	105.6350
6	Bip-hPheP[CH_2]Gly-OMe	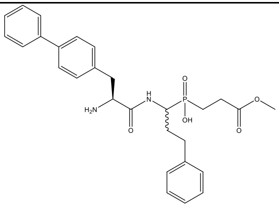	*SR*	73.7767	83.6079
			SS	86.0585	81.2882
7	Bip-LeuP[CH_2]Gly-OMe	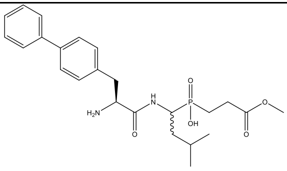	*SR*	73.9310	90.4322
			SS	70.3871	81.1540
8	Bpa-hPheP[CH_2]Gly-OMe	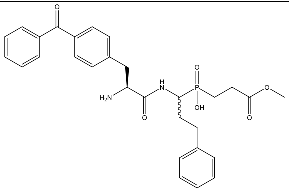	*SR*	89.0527	94.5523
			SS	83.6289	83.6797
9	Bpa-LeuP[CH_2]Gly-OMe	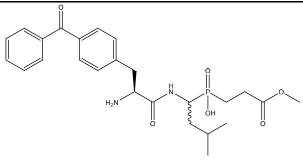	*SR*	85.7678	80.6959
			SS	87.8818	80.7119

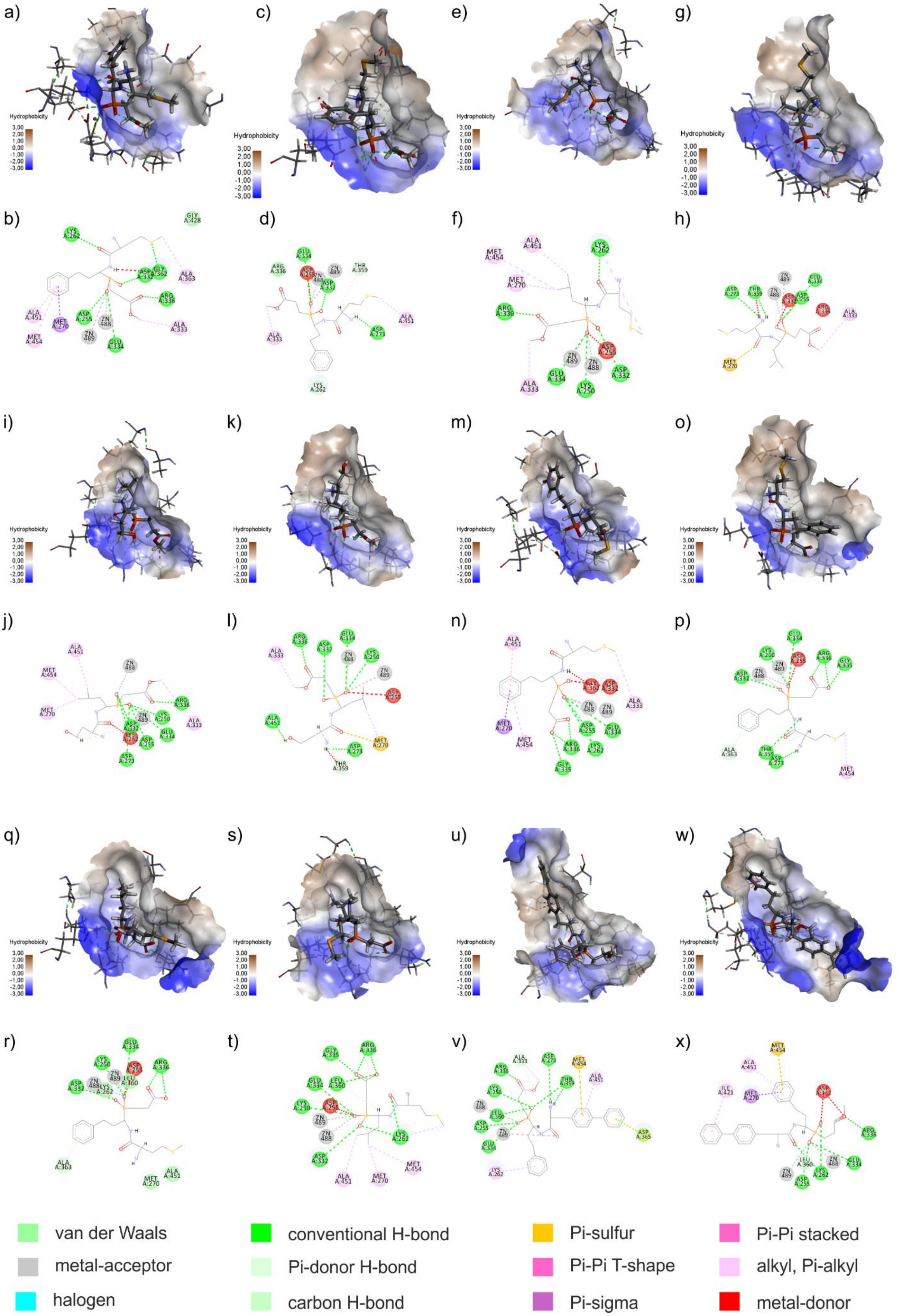

Figure S27. Modes of binding of all the studied phosphinic pseudotripeptides by bovine lens leucyl aminopeptidase [PDB ID: 1LCP] with showed specific intermolecular interactions: a),b) **1**-*SR* isomer; c),d) -**1**-SS isomer; e),f) **2**-*SR* isomer; g),h) **2**-SS *isomer;* i),j) **3**-*SR* isomer*;* k),l) **3** – *SS* isomer; m),n) **4** -*SR* isomer; o),p) **4** – *SS* isomer; q),r) **5** -*SR* isomer; s),t) **5** -*SS* isomer; u),v) **6** – *SR* isomer; w),x) **6** – *SS* isomer.

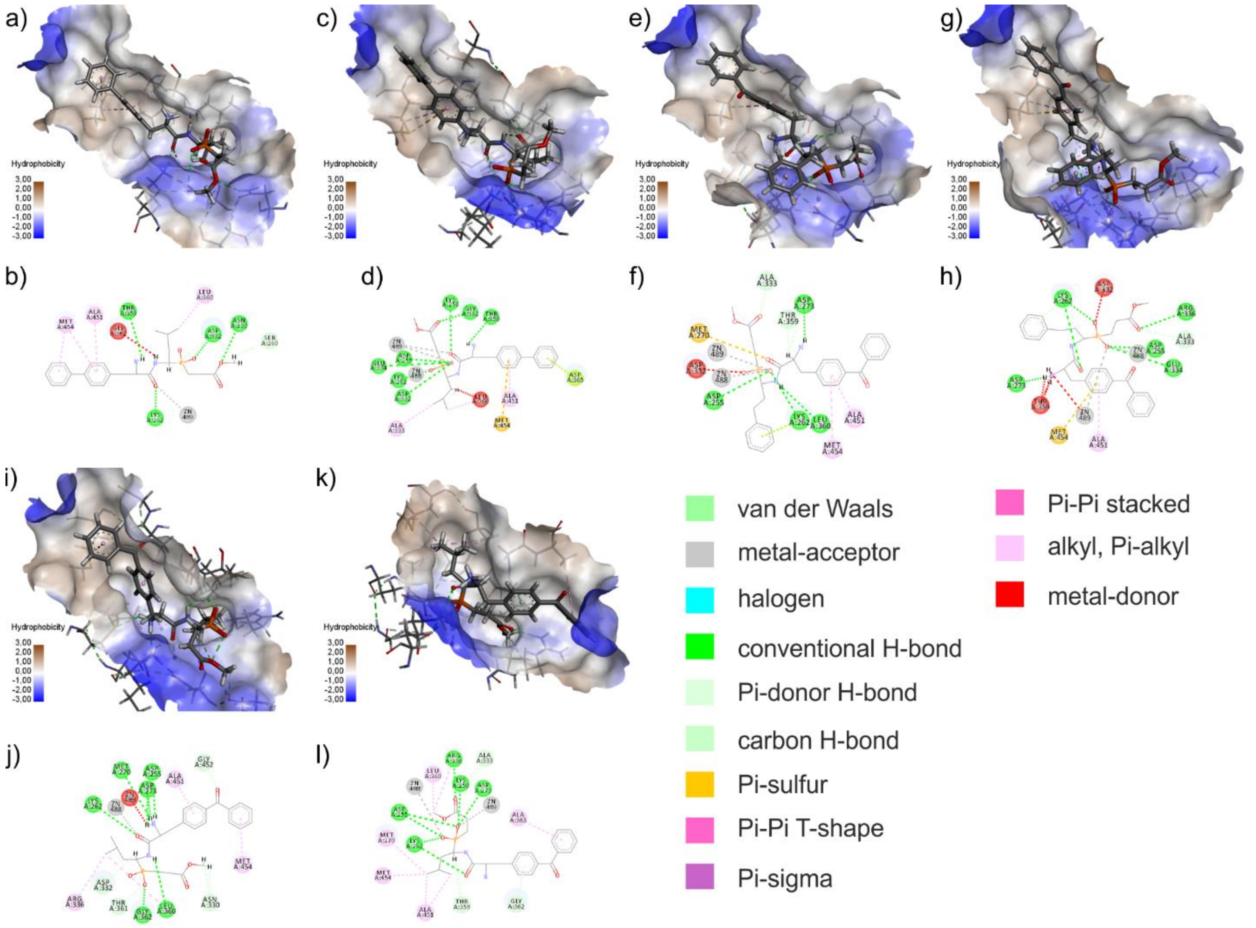

Figure S28. Modes of binding of all the studied phosphinic pseudotripeptides by bovine lens leucyl aminopeptidase [PDB ID: 1LCP] with showed specific intermolecular interactions: a),b) **7**-*SR* isomer; c),d) **7**-SS isomer; e),f) **8**-*SR* isomer; g),h) **8**-SS *isomer;* i),j) **9**-*SR* isomer*;* k),l) **9** – *SS* isomer**.**

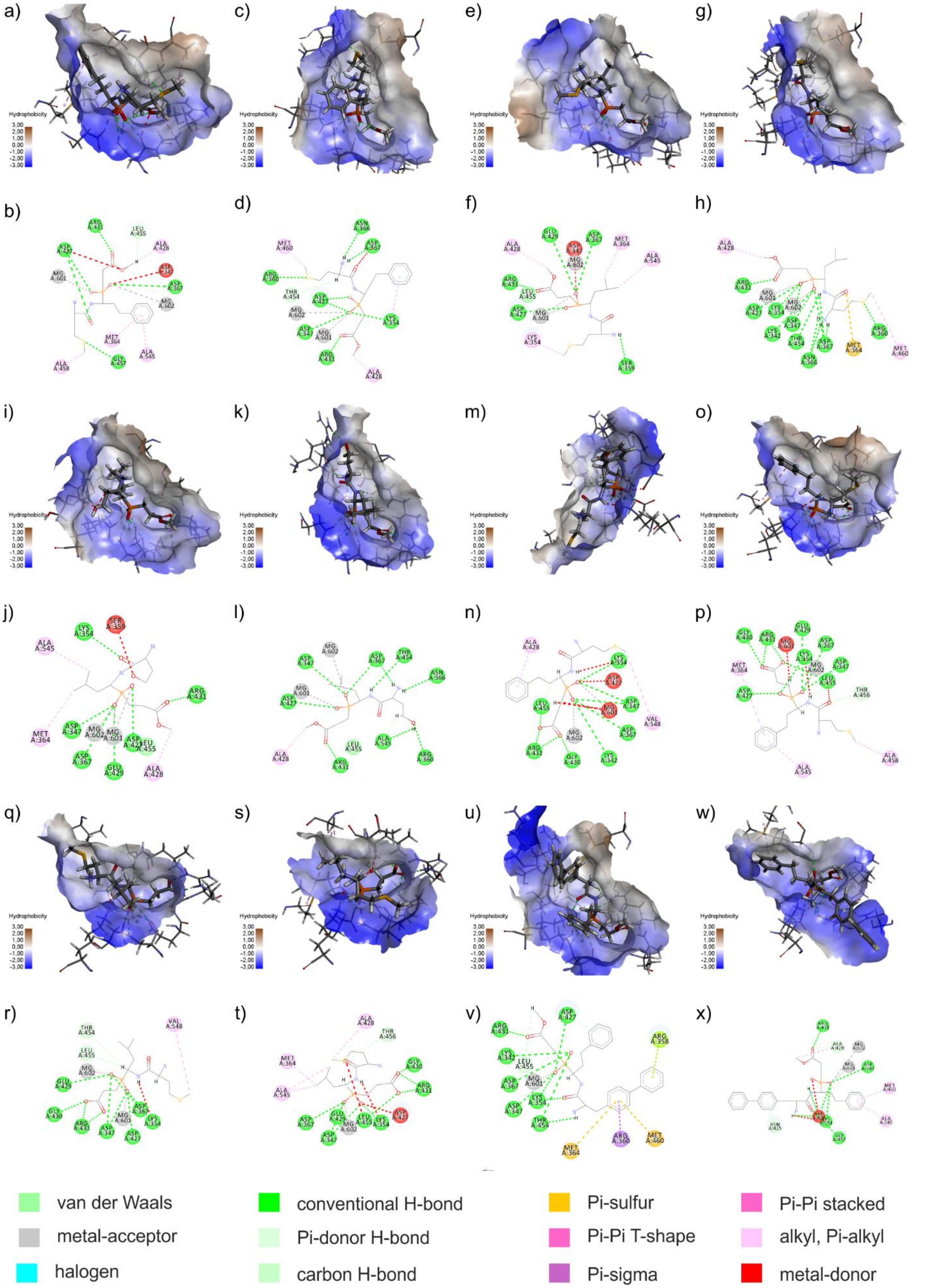

Figure S29. Modes of binding of all the studied phosphinic pseudotripeptides by tomato amino peptidase [PDB ID: 4KSI] with showed specific intermolecular interactions: a),b) **1**-*SR* isomer; c),d) -**1**-SS isomer; e),f) **2**-*SR* isomer; g),h) **2**-SS *isomer;* i),j) **3**-*SR* isomer*;* k),l) **3** – *SS* isomer; m),n) **4** -*SR* isomer; o),p) **4** – *SS* isomer; q),r) **5** -*SR* isomer; s),t) **5** -*SS* isomer; u),v) **6** – *SR* isomer; w),x) **6** – *SS* isomer.

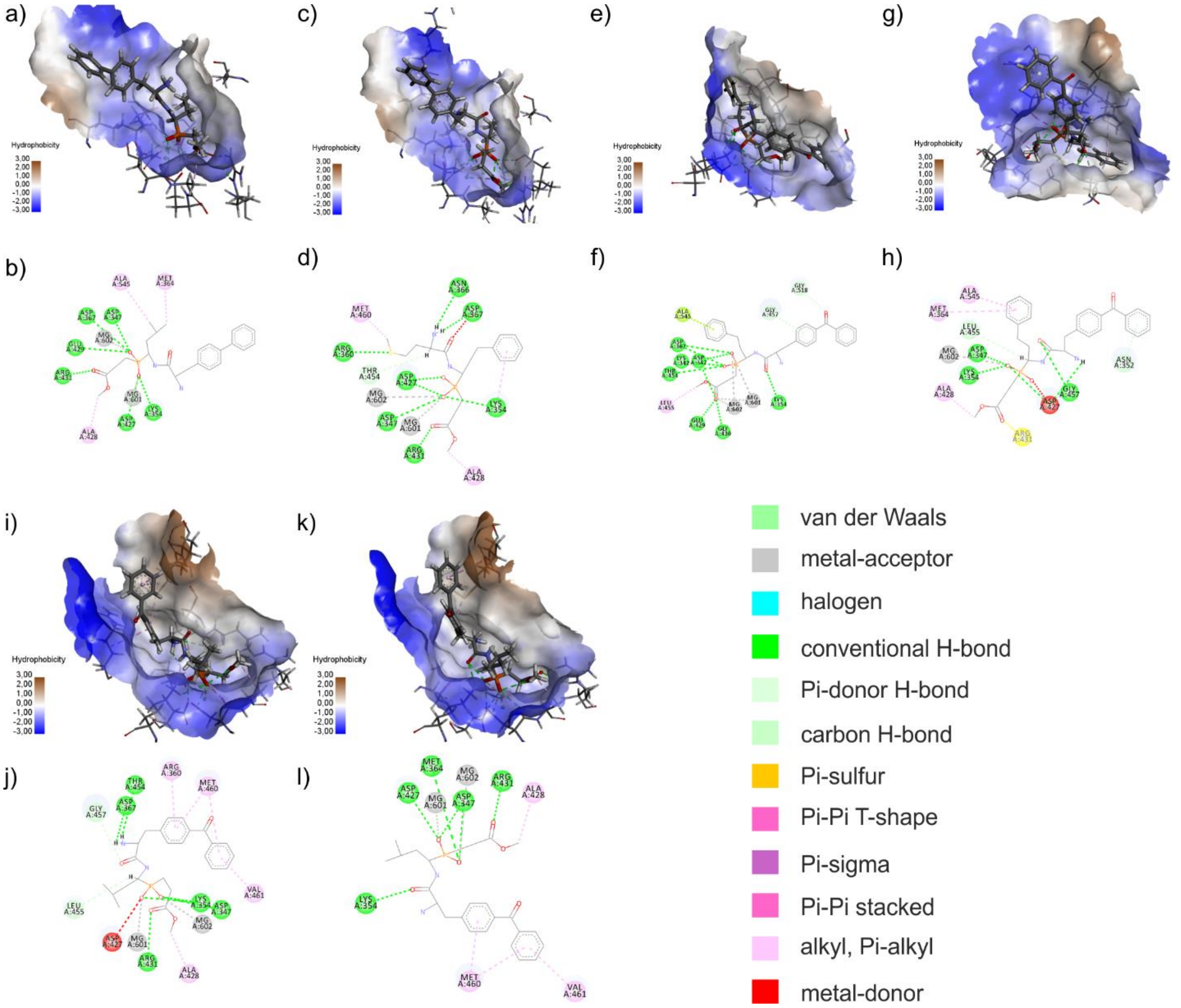

Figure S30. Modes of binding of all the studied phosphinic pseudotripeptides by tomato aminopeptidase [PDB ID: 4KSI] with showed specific intermolecular interactions: a),b) **7**-*SR* isomer; c),d) **7**-SS isomer; e),f) **8**-*SR* isomer; g),h) **8**-*SS* isomer; i),j) **9**-*SR* isomer; k),l) **9**-*SS* isomer.
